# A scoping review of the globally available tools for assessing health research partnership outcomes and impacts

**DOI:** 10.1186/s12961-023-00958-y

**Published:** 2023-12-22

**Authors:** Kelly J. Mrklas, Jamie M. Boyd, Sumair Shergill, Sera Merali, Masood Khan, Cheryl Moser, Lorelli Nowell, Amelia Goertzen, Liam Swain, Lisa M. Pfadenhauer, Kathryn M. Sibley, Mathew Vis-Dunbar, Michael D. Hill, Shelley Raffin-Bouchal, Marcello Tonelli, Ian D. Graham

**Affiliations:** 1 Department of Community Health Sciences, Cumming School of Medicine, University of Calgary, 3D10-3280 Hospital Drive NW, Calgary, AB T2N 4Z6 Canada; 2 Strategic Clinical Networks™, Provincial Clinical Excellence, Alberta Health Services, Calgary, AB Canada; 3 Knowledge Translation Program, St Michael’s Hospital, Li Ka Shing Knowledge Institute, Unity Health Toronto, Toronto, ON Canada; 4 Cumming School of Medicine, University of Calgary, Calgary, AB Canada; 5 Faculty of Kinesiology, University of Calgary, Calgary, AB Canada; 6 Department of Community Health Sciences, University of Manitoba, Winnipeg, MB Canada; 7 Faculty of Nursing, University of Calgary, Calgary, AB Canada; 8 Faculty of Science, University of Alberta, Edmonton, AB Canada; 9 Institute for Medical Information Processing, and Epidemiology-IBE, Ludwig-Maximilians Universität Munich, Munich, Germany; 10 Pettenkofer School of Public Health, Munich, Germany; 11 George & Fay Yee Centre for Healthcare Innovation, University of Manitoba, Winnipeg, MB Canada; 12 University of British Columbia-Okanagan, Kelowna, BC Canada; 13 Departments of Clinical Neurosciences, Medicine and Radiology, Cumming School of Medicine, University of Calgary, Calgary, AB Canada; 14 Hotchkiss Brain Institute, Cumming School of Medicine, University of Calgary, Calgary, AB Canada; 15 Department of Medicine, Cumming School of Medicine, University of Calgary, Calgary, AB Canada; 16 Office of the Vice-President (Research), University of Calgary, Calgary, AB Canada; 17 Clinical Epidemiology Program, Ottawa Hospital Research Institute, Ottawa, ON Canada; 18 Schools of Epidemiology and Public Health and Nursing, University of Ottawa, Ottawa, ON Canada

**Keywords:** Health research partnerships, Outcomes, Impacts, Evaluation tools, Scoping review, Integrated knowledge translation, Community-based participatory research

## Abstract

**Background:**

Health research partnership approaches have grown in popularity over the past decade, but the systematic evaluation of their outcomes and impacts has not kept equal pace. Identifying partnership assessment tools and key partnership characteristics is needed to advance partnerships, partnership measurement, and the assessment of their outcomes and impacts through systematic study.

**Objective:**

To locate and identify globally available tools for assessing the outcomes and impacts of health research partnerships.

**Methods:**

We searched four electronic databases (Ovid MEDLINE, Embase, CINAHL + , PsychINFO) with an a priori strategy from inception to June 2021, without limits. We screened studies independently and in duplicate, keeping only those involving a health research partnership and the development, use and/or assessment of tools to evaluate partnership outcomes and impacts. Reviewer disagreements were resolved by consensus. Study, tool and partnership characteristics, and emerging research questions, gaps and key recommendations were synthesized using descriptive statistics and thematic analysis.

**Results:**

We screened 36 027 de-duplicated citations, reviewed 2784 papers in full text, and kept 166 studies and three companion reports. Most studies originated in North America and were published in English after 2015. Most of the 205 tools we identified were questionnaires and surveys targeting researchers, patients and public/community members. While tools were comprehensive and usable, most were designed for single use and lacked validity or reliability evidence. Challenges associated with the interchange and definition of terms (i.e., outcomes, impacts, tool type) were common and may obscure partnership measurement and comparison. Very few of the tools identified in this study overlapped with tools identified by other, similar reviews. Partnership tool development, refinement and evaluation, including tool measurement and optimization, are key areas for future tools-related research.

**Conclusion:**

This large scoping review identified numerous, single-use tools that require further development and testing to improve their psychometric and scientific qualities. The review also confirmed that the health partnership research domain and its measurement tools are still nascent and actively evolving. Dedicated efforts and resources are required to better understand health research partnerships, partnership optimization and partnership measurement and evaluation using valid, reliable and practical tools that meet partners’ needs.

**Supplementary Information:**

The online version contains supplementary material available at 10.1186/s12961-023-00958-y.

## Background

Health research partnerships involve researchers engaging with diverse partners, including patients, decision or policy makers, health care administrators and healthcare or community agencies, among others, in any or all parts of the research process [1, 2]. Numerous health research partnership approaches or traditions have independently evolved over the past half century, including participatory research, co-production, mode 2 research, engaged scholarship and integrated knowledge translation, among others [3]. The increasing popularity of partnership approaches is promising [4] because partnerships are known to help enhance our understanding of key ‘factors that facilitate and hinder the development and sharing of knowledge in healthcare systems’ (p. 2) [5] and to increase the relevance, use, sustainability and impact of research [6–8]. For partners themselves [9], the increased popularity of research partnerships creates new opportunities for greater equity [7], shared power, trust, synergy, capacities and sustainability in health research and for generating non-traditional benefits for partners and researchers alike [7, 9–14].

However, while the qualitative and anecdotal value of these approaches is well established [1, 7, 13, 15–25], their systematic, causal and quantified measurement is not. Partnership measurement has lagged behind [26, 27], despite increasing demand for tangible evidence of the resulting outcomes and impacts [28–31]. With increasing fiscal constraints in health and health research sectors, the need to understand and link health research partnerships to real-world outcomes and impacts is paramount. However, tangible examples of studies assessing the causal influences of health research partnerships on outcomes and impacts are few [7, 8, 24, 32–34]. Findings generated by researchers at the Center for Participatory Research at the University of New Mexico [35] and their collaborating teams provide strong examples of theorized, quantified partnership outcomes and impacts [36–39]. Similarly, King and colleagues [27, 40] also provide a strong example of partnership impact measurement.

In this review, we refer to outcomes as measurable factors that change as a result of intervention(s) and that are not futuristic, including process and summative outcomes (adapted from University of Waterloo, 2018 and Hoekstra et al., 2018) [1, 41] and impacts as effects, influences or changes to the economy, society, public policy or services, individuals, teams, organizations, health, the environment or quality of life beyond academia (adapted from the Higher Education Funding Council of England, 2014 and Hoekstra et al., 2018) [1, 42] (Table 1).

**Table 1:** Key terms and definitions

Key term	Definition
Health research partnership [1, 2]	‘Partnerships involving individuals, groups, or organizations engaged in collaborative health research activity involving at least one researcher (e.g., an individual affiliated with an academic department, hospital or medical centre), and any partner actively engaged in any part of the research process (e.g., decision or policy maker, health care administrator or leader, community agency, charities, network, patients, industry partner, etc.).’ A health research partnership may encompass a diverse set of research activities, including (but not limited to) integrated knowledge translation (IKT), community-based participatory research (CBPR), action research or participatory action research (PAR), collaborative research, co-design and academic-community partnerships
Tool [1, 3]	‘An instrument (survey, measures, assessments, questionnaire, inventory, checklist, list of factors, subscales or similar) that can be used to assess the outcome or impact elements or domains of a health research partnership.’
Outcome (adapted from University of Waterloo, 2018) [1, 4]	‘…factor(s) described in the study methods used to determine a change in status as a result of interventions, can be measured or assessed as component(s) of the study, and are not futuristic’; including both process and summative outcomes
Impact [1, 5]	‘…effects, influences, or changes to the economy, society, public policy or services, individuals, teams, organizations, health, the environment, or quality of life, beyond academia.’
Context [1, 6]	‘The physical, organizational, institutional, and legislative structures that enable and constrain, and resource and realize, people and procedures.’

There are many documented challenges for measurement in this field, with multiple contributing causes, including the sheer diversity of partnership approaches [43], the type and maturity of evaluative designs and an historical inclination towards qualitative designs and methods [31, 32]. This context makes cross-partnership comparisons and transferability of findings challenging [7, 11–13]. Other reported measurement complexities pertain to a lack of measurement neutrality, a lack of clarity around outcome and impact terms, definitions and their inconsistent application [31], and the positioning of health research partnership outcomes and impacts as secondary objectives or incidental findings in research reports. These factors hinder measurement advancements and the ability to draw causal links between the influence of partnerships and their outcomes and impacts [24, 31].

Furthermore, researchers report a lack of theoretical foundations, validated, psychometrically-tested and pragmatic assessment tools [23, 24, 29], and objective (instead of proxy or self-reported measures) [32, 33] among their key measurement concerns [7, 13, 23, 32]. For the last 20 years, there have been recurrent calls to develop more quantitative, pragmatic, generalizable and flexible tools to better understand partnership establishment, processes, outcomes and impacts [12, 16, 28, 29, 44–47]. There is increasing demand for valid, reliable and pragmatic measures to assess the nature, type, and dose of health research partnership activities necessary to optimize outcomes and impacts, while minimizing costs and harms [13, 23, 24, 28, 31, 48]. Optimizing health research partnership design, execution and evaluation in the future is predicated on the extent to which partnership outcomes and impacts measures and measurement evolves [23, 27].

Finally, multiple, pre-existing reviews exist in this research domain. However, many of these reviews are narrowly focussed on research partnership evaluation tools for specific populations [24, 28, 48], specific partnership traditions or health-inclusive domains [7, 10, 13, 29, 44, 49–51], or on the quality and outcomes of research collaborations [23]. This review adds a unique perspective in attempting to locate and describe globally available tools for health research partnership outcome and impact assessment without restriction on population, tradition, domain, partnership elements or specific types of outcomes and impacts. The review is pragmatic by design and motivated by the need to offer researchers and stakeholders alike ready access to tools for assessing research partnership outcomes and impacts.

## Research questions

The primary research question is: what are the globally available tools for assessing the outcomes and impacts of health research partnerships in the published literature? Our secondary research questions are: what is the nature and scope of the literature, including relevant terminology, study characteristics, tool, tool evaluation; and partnership characteristics, emergent gaps, future research questions, and what is the feasibility for conducting a systematic review of the identified tools?

## Methods

This scoping review was designed to identify and describe tools for assessing the outcomes and impacts of health research partnerships, and is guided by a collaboratively built conceptual framework [1]. The detailed scoping review protocol [52] outlining the objectives, inclusion criteria and methods was specified a priori and posted to the Open Science Framework [53], prior to full text abstraction. Protocol deviations and rationale are detailed in the supplementary file (Additional file 1: Appendix 2). Expanded methods are provided in the supplementary file (Additional file 1: Appendix 3).

## Search strategy and data sources

An a priori search strategy was developed from relevant keywords, publication indexing and Medical Subject Headings (MeSH) in consultation with a medical research librarian (MVD) (Additional file 1: Appendix 4). Four electronic health research databases [MEDLINE (OVID), EMBASE, CINAHL Plus, PsychINFO] were searched from inception to 21 October 2018 with two updates (31 December 2019 and 2 June 2021). The search yielded 36 027 unique citations.

We defined a health research partnership as ‘…individuals, groups or organizations engaged in collaborative, health research activity involving at least one researcher (e.g., individual affiliated with an academic department, hospital or medical centre), and any partner actively engaged in any part of the research process (e.g., decision or policy maker, health care administrator or leader, community agency, charities, network, patients, industry partner, etc.)’ [1, 2]. Tools were defined as ‘instruments (e.g., survey, measures, assessments, questionnaire, inventory, checklist, questionnaires, checklists, list of factors, subscales or similar) that can be used to assess the outcome or impact elements or domains of a health research partnership’ [1, 54]. An outcome was defined as ‘factor(s) described in the study methods used to determine a change in status as a result of interventions, can be measured or assessed as component(s) of the study, and are not futuristic’; including both process and summative outcomes (adapted from Hoekstra et al., 2018; University of Waterloo, 2018) [1, 41]. Impact was defined as ‘any effect, influence on, or change to the economy, society, public policy or services, individuals, teams, organizations, health, the environment, quality of life or academia’ (adapted from Hoekstra et al., 2018; Higher Education Funding Council for England) [1, 42] (Table 1). Remaining operational terms and definitions are provided in Additional file 1: Appendix 2 and online [1, 52].

### Eligibility and screening

We retained studies describing a health research partnership and the development, use and/or assessment of a health research partnership outcome or impact assessment tool (or element of, or at least one health research partnership outcome or impact measurement property [49, 55] of a tool), as an aim of the study (Table 2).

**Table 2:** Study inclusion and exclusion criteria

Inclusion criteria	Exclusion criteria
Include studies: (a) pertaining to, describing or involving a health research partnership; (b) involving the development, use and/or assessment of a health research partnership outcome or impact assessment tool (or element/property of a tool), as an aim of the study (and inclusive of multi-tool or toolkit studies and studies involving frameworks/models when accompanied by a tool); (c) that are accessible and amenable to full text review; (d) reporting primary research findings drawn from empirical evidence; (e) reporting relevant abstractable data; (f) of any design type, that meet eligibility criteria	Exclude studies that: (a) do not meet the definition of a health research partnership; (b) involve researcher–researcher or interprofessional (non-researcher inclusive) healthcare team partnerships; (c) do not involve the development, use and/or assessment of a health research partnership tool (or element/property of a tool), as an aim of the study; (d) are not available or amenable to full text review; (e) report head-to-head tool comparisons without separately reporting tool-specific findings; (f) do not report primary research findings drawn from empirical evidence; (g) lack adequate or relevant abstractable data

All title, abstract and full text screening was undertaken independently and in duplicate. We used a hybrid strategy involving independent abstraction (K.J.M) and independent validation by a second, trained investigator (M.K., S.S., S.M.) in the data abstraction phase [56], with all discrepancies resolved with consensus by dual review, discussion at weekly meetings and guided by a pilot-tested tool and coding manual [57–59]. Variables pertaining to study characteristics, tool characteristics, partnership characteristics and tool evaluation characteristics, were abstracted according to the protocol [52]; and Additional file 1: Appendix 2.

## Tool evaluation criteria

We adapted consensus-built criteria developed by Boivin and colleagues to arrive at a final set of 20 criteria and companion scoring rubric [28, 60] (Additional file 1: Appendix 5).

### Analysis

We synthesized key study, tool, tool evaluation and partnership characteristics (Additional file 1: Appendix 2) using basic descriptive statistics (mean/standard deviation, frequency counts) for tabular presentation using MS Excel [61] and Stata v13.1 [62]. We analysed qualitative data in NVivo v12.7 [63] using an inductive thematic approach [64] and a descriptive-analytical process for reviews [65] and reported findings according to guidelines [66–68].

## Results

**Fig. 1:**
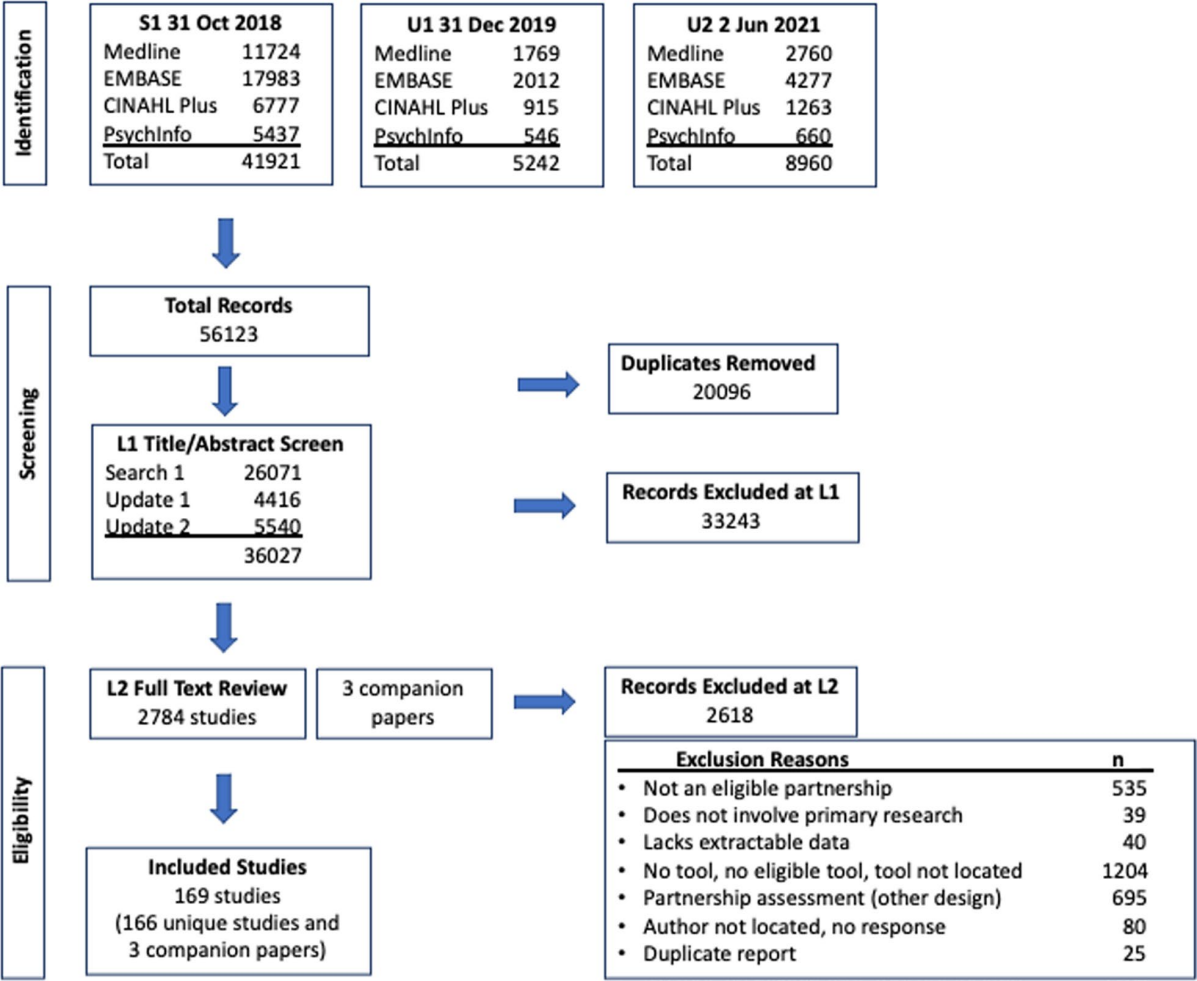
Scoping review PRISMA study flow diagram

The initial search (31 Oct 2018) and updates (31 December 2019 and 2 June 2021) generated 36 027 de-duplicated citations, and of these, 2784 full text reports were retrieved for evaluation, ultimately yielding 169 studies (166 unique studies with three companion reports). Companion reports comprised published protocols and a tool language translation study. Study citation flow is provided in the Preferred Reporting Items for Systematic Reviews and Meta-Analyses (PRISMA) diagram (Fig. 1).

The most common reasons for exclusion were studies lacking tools or lacking tools that assessed partnership outcomes/impacts (*n* = 1204), followed by studies involving outcomes and impacts assessment by another method that did not match the study definition of a tool (e.g., involved other modalities or methods of assessment, such as focus groups, interviews, evaluative approaches such as social network analysis, etc.) (*n* = 695). ‘Substantial’ inter-rater agreement [69, 70] was achieved at L1 title/abstract [Cohen’s *κ*: 0.66 95% confidence interval (CI) (0.64–0.67)] and L2 full text [Cohen’s *κ*: 0.74 95% CI (0.72–0.76)] review stages.

### Study characteristics

Included studies were distributed across a broad scope of peer-reviewed journals. Just under half of included studies (45%, 75) were clustered in 10 journals and several smaller clusters located in three others (5%, 9). The remainder (82) was widely dispersed across 72 other journals and a single government report.

In total, 24 countries were represented by eligible studies; most studies were located in minority countries. Minority countries refer to locations where the minority of the global populace resides and replaces the outdated term ‘developed’ nations (Additional file 1: Appendix 2). We found 157 single-site and nine multi-site studies in the data set. Of the single-site studies, 109 originated in North America (69%); 86 studies from the United States and 23 from Canada (79% and 21%, respectively). A further 36 studies originated from Europe (23%), including the United Kingdom (21), Ireland (5), The Netherlands (4), Germany (2), Spain (2), Sweden (1) and Denmark (1). A smaller number of studies originated from Australasia (12, 8%) [Australia (10), New Zealand (1), Taiwan (1)]; we also located one eligible single-site study in the Middle East (1, 1%). Of the nine multi-site studies identified (5%), four involved minority countries (Canada, Australia, New Zealand, United States, Mexico), leaving a very small proportion of the literature originating from majority countries, including South America (Argentina, Bolivia, Brazil, Chile, Columbia, Peru), African nations (South Africa, Uganda, Ghana) and a single site in the Caribbean (Saint Lucia). With only one exception, no studies originated from majority countries alone, and where majority countries were involved, all were partnered with minority country partners. Majority countries refer to locations where the majority of the global populace resides and replaces the outdated term ‘developing’ nations (Additional file 1: Appendix 2).

Additional file 2: Table S1 reports key characteristics of included studies. More than half of included studies were published after 2015 (91, 55%); there was a steady increase in the eligible health research partnership literature over the last 30 years (Additional file 1: Appendix 6).

All but one eligible study was published in the English language (99%, 165); however, we also identified six studies containing English–French (2) [71–73] and English–Spanish (4) [36, 74–76] bilingual tools, respectively, and four other studies with German [77], French [78], Spanish [79] and Dutch [80] language tools.

**Fig. 2:**
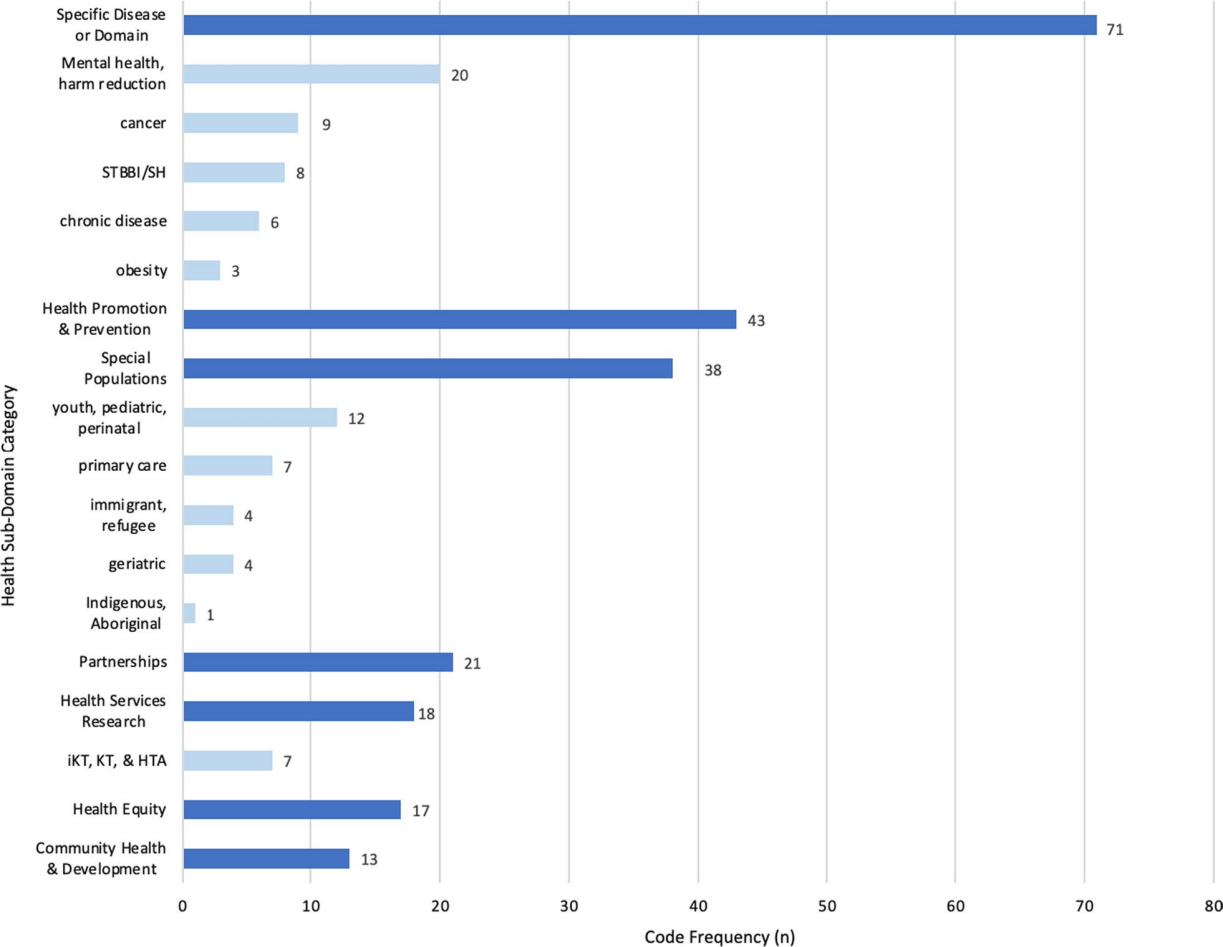
Health sub-domains and key sub-domain cluster. *where necessary, ≧ 1 sub-domain code per study was allowed, resulting in 221 sub-domain codes *n* = 166 studies. *STBBI* sexually transmitted and blood borne infections, *KT* knowledge translation, *IKT* integrated knowledge translation, *HTA* health technology assessment

Diverse health sub-domains were represented by included studies (Fig. 2). We coded 221 health sub-domains, organized into seven themes, including disease-specific (71, 32%), health promotion and prevention (43, 22%), special populations (38, 17%), partnerships (21, 10%), health services research (18, 8%), health equity (17, 8%), and community health and development (13, 6%) studies. The most frequently occurring study designs were mixed methods designs (79, 48%), cross-sectional (58, 35%) and case or multiple case study designs (16, 10%). The remaining study designs comprised nested, descriptive, pre-post or post-test, Delphi and qualitative surveys (13, 9%). The methods employed in these studies were primarily mixed (122, 73%), followed by quantitative (38, 23%) and qualitative (6, 4%) methods. Of the mixed methods utilized, 88% (106) were mixed quantitative–qualitative, 10% (12) were multi-qualitative methods and 3% (4) were multi-quantitative methods.

Most studies described multiple activities pertaining to one or more aspects of tool development (101, 61%), modification (52, 31%), use (142, 86%), evaluation (26, 16%) and validation (49, 30%). Conceptually, 119 (72%) studies cited an underlying framework or model, 12 (7%) generated a new framework or model during the study, and nine studies (5%) were both based on and generated a new framework or model. Most studies reported an evaluation of both outcomes and impacts (94, 57%), followed by outcomes (61, 37%), and impacts alone (11, 6%); however, we note these terms were frequently interchanged within and among study reports.

The sex of individuals filling out partnership assessment tools was reported in 33% of studies (54), and in 7% (11) reporting was incomplete. In a further 4% of studies (6), sex was requested but not reported. When sex was reported, the overall crude mean proportion of female participants across 54 studies was 67.1% [standard deviation (SD) 0.15]. A weighted mean average could not be calculated due to the frequent absence of denominator data. Other key social variables were not consistently available for reporting.

### Tool characteristics

Additional file 2: Table S2 summarizes key characteristics of the tools in included studies. Overall, 205 tools were identified, and of these, surveys and questionnaires were the most frequently reported tool type (100, 49% and 66, 32%, respectively). We noted that the terms survey and questionnaire were frequently interchanged within study reports; when this occurred, we elected the term most frequently associated with the methodological description of the tool. Scales were the third most frequent type of tool (15, 7%) and the remaining tools comprised indices, checklists, rubrics, criteria, and logs (11, 5%). We also identified a number of studies that employed toolkits (multiple tools in combination or as part of a process) (13, 6%), to assess health research partnership outcomes and impacts (Table 4). More than two thirds of tools were underpinned by a conceptual framework or model (144, 70%), but very few cited a review (e.g., synthesis or other review, or informed by a search of > 1 electronic databases with reported time frame) as underlying evidence informing the tool (35, 17%). In slightly more than a third of studies, we were able to find explicit reference to tool validity (63, 38%) and reliability evidence (59, 36%), but most involved self-reported measures of perception (161, 97%).

There was a high degree of shared provenance among the tools. Many tools referred to the adoption or modification of components from one or more pre-existing tools. From the studies that reported tool provenance, we were able to identify several distinct clusters of tools comprising derivations, modifications, or applications of a single tool. There were eight clusters (70 studies) linked to early tools and related research conducted by Israel, Lantz, Schulz and colleagues (17) [15, 81–85], Wallerstein and colleagues (13) [19, 86–90], Butterfoss, Goodman, Wandersman and colleagues (10) [46, 91–98], Weiss, Lasker and colleagues, (8) [99–103], Feinberg, Brown, Chilenski and colleagues (6) [104–109], Abelson and colleagues (6) [110–113], Forsythe and colleagues, the Patient-Centered Outcomes Institute (PCORI) (5) [114–116], and Jones and Barry and colleagues (5) [117–119]. We also noted significant cross-referencing among the clusters.

In more than a third of studies, the specific partner group affiliation for those filling out tools was not provided (61, 37%). Where partners were defined, we sorted these 222 reported targets into different 13 partnering groups. The most frequently described partner groups targeted by tools were researchers (68, 31%), followed by patients and the public (54, 24%), community members (24, 11%), health care systems stakeholders (21, 9%), coalition staff (15, 7%), partner organizations (15, 7%) and research staff (14, 6%). The remaining stakeholders comprised government (3), policymakers, education sector staff, research funders and reviewers (2, respectively), decision makers and industry partners (1, respectively). In 75% of eligible studies, two or more partner groups were targeted by health research partnership outcomes and impacts tools; few studies targeted only a single partner group for health research partnership outcomes and impacts assessment.

### Partnership characteristics

As anticipated, we were able to identify an array of research partnership approaches from authors’ partnership descriptions (Table 3). Community-based participatory research approaches arose most frequently in the data set, and included both CBPR (47, 23%) and organizational-based participatory research (OBPR) (3, 1%). General partnership approaches were the next most frequent category (32, 16%), followed by patient and public involvement (PPI) (26, 13%) and coalitions (22, 11%).

**Table 3:** Key partnership approaches and partnership terms (*n*=166)

Approach domain	Code frequency (*n* = 206)	Partnership approach	Terms used to describe the partnership	# Unique terms
Community-based participatory research (CBPR)	47	• Community-based participatory research (CBPR)	• Coalition, community coalitions, community-based coalition, coalition community intervention, alliance, community capacity, networks of partners, prevention initiative, substance abuse prevention coalitions, partnership, physical activity coalition, collaborative, collaboration, collaborative network, community engagement, stakeholder engagement	16
	3	• Organizational-based participatory research (OBPR), organizational collaboration		
Partnership	32	• Partnership, public–private partnership, community health partnership, health partnership, health promotion partnership, community/community-based partnership, collaborative research partnership, intersectoral partnership, multistakeholder partnership, global health research partnership, research partnership, patient partners, social capital • Community–academic or academic–community partnership, academic–public health agency partnership, community–university research partnership, researcher–community partnership, community–researcher partnership, academic–practice partnerships, academic–practitioner collaboration, researcher–stakeholder partnerships	• Partnership, community health partnerships; community partnership, local health partnership; community partnership for health, community-based partnership, community care network, community-based alliances, public private partnerships, coalition, health promotion partnership, community coalition, community health improvement interventions, clinic–community partnership, intersectoral action, intersectoral partnership, healthcare organization–research partnership, healthcare partnership, organization–university-based research partnership, patient engagement, patient partners, engaged research partners, patient advisory council, advisory committee–stakeholder collaboration, stakeholder engagement • Community–academic partnership, community partnership, partnership scheme; health and social partnership schemes, academic–agency partnership, community–university research partnership, academic–community collaboration, community engagement, collaborative research partnership, collaboration, impact partnership, multidisciplinary community–university research partnerships, community–researcher collaborations, academic practice partnership; academic service partnership, global health research partnership, research partnership, collaborations for leadership in applied health research and care, research team–stakeholder partnership	45
Patient and public involvement, patient involvement (PPI, PI)	26	• Patient and public involvement, patient involvement, public involvement • Patient-powered research network (PPRN), patient-centred outcomes research (PCOR), patient governance, patient-centred research	• PPI, engagement, involvement, patient/public/participant involvement, patient research partner involvement, young people with a chronic condition (YPCC) involvement, patient working group (PWG), patient engagement, engagement in research, community engagement, patient-centred health research; collaboration, patient and public involvement initiatives, co-working; public advisory group, PPI strategy, co-design, patient participation, health services research, participatory research, research partnership, translational and lab-based research, participatory research design, participatory research team	24
Coalition	22	• Coalition, community coalition, prevention and health promotion coalition, community-based health promotion coalition, health promotion coalition, community health coalition, community-based coalition, substance abuse prevention coalitions, ecological approach	• CBPR, CBPR partnership, CBPR-guided academic-community partnership, community-based participatory partnership, research partnership, coalition, community coalition, steering committees, networks of partners, prevention initiative, partnerships for quality, participatory research, partnership, collaboration, collaborative, collaborative partnership, collaborative effort, collaborative network, participatory research, community health partnerships, outreach, community partnership, collaborative research partnership, impact partnership, community capacity, community networks, occupational health partnerships, capacity building coalition, technical assistance, community advisory board • Multidisciplinary community–university research partnerships, community–academic–government partnership, community institutional relations, community–academic partnership, community–academic research partnership, researcher–community partnership, health research partnership, partnership engagement, transdisciplinary collaborative centres, transdisciplinary collaborations, patient engagement, stakeholder engagement, community engagement and research, stakeholder-engaged research, community engaged research process, academic–community collaboration, academic and community partners, community engagement, multinational partnership, collaborative KE workshop, stakeholder engagement	50
Participatory research	13 2 2	• Participatory research, action research (AR), participatory action research (PAR), participatory action, community-based participatory action research (CBPAR), participatory research design, patient engagement in participatory research • Collaborative or participatory evaluation • Coordinated action, collective action	• Participatory research, participatory action research, participatory research team, community-based participatory research, action research partnership, collaborative partnership, partnership, healthcare organization–research partnership, multi-stakeholder partnership, healthcare organisation–university-based research partnership, research partnership, community partnership, participatory intervention, participatory programmes, patient and other stakeholder engagement or involvement; engagement; innovation teams, collaboration, both-way learning (reciprocal knowledge co-creation), PPI, participatory research design, co-design, participant involvement, stakeholder engagement • Action research, collaboration, evaluation, community health promotion, partnership, intersectoral action	30
Patient/patient and public engagement (PE, PPE)	13	• Patient engagement, patient and public engagement, public engagement, engagement in health research	• PAG-investigator partnership; patient engagement, research practice collaborative, partnership, community engagement, patient involvement, young people’s advisory group (YPAG), partners, patient partners, patient advisory councils, public and patient engagement, research partnership, patient and family caregiver engagement in research, patient input, patient experience, stakeholder engagement	16
Community engaged research (CEnR or CER)	10	• Community engaged research	• Coalition, community health coalition, partnership, collaborative research partnership, community partnership, impact partnership, multidisciplinary community–university research partnerships, community–academic partnership, academic-community collaboration, research partnership, community/stakeholder engagement	11
Consumer involvement in research	9	• Consumer involvement, consumer involvement in research, user involvement in research, service user involvement, service user research, consumer and community participation, user-led research, consumer-led research, service user-led research	• Consumer involvement in research, evaluation, user involvement in research, service user involvement, consumer and community reference group, partners, service user leadership, service user research, user led research, co-production	10
Community engagement (CE)	8	• Community empowerment, community participation in research, stakeholder engagement, stakeholder engaged research	• Collaboration, partnership, public involvement, co-working, public advisory group, stakeholder participation, patient–provider partnership, community/patient/stakeholder engagement, community–academic partnership, community engagement and research, stakeholder-engaged research, advisory committee–study stakeholder collaboration, research collaboration, research team–stakeholder partnerships	14
Co-research	8	• Co-research, patient co-research, co-working, knowledge co-production, research co-production, co-produced research, co-production, pediatric co-research, participatory theme elicitation	• PPI, engagement, involvement, patient coinvestigator, healthcare organization–research partnership, partnership, healthcare organisation–university-based research partnership, young people’s advisory group (YPAG), PPI strategy, co-design, co-research, collaboration, patient involvement	13
Integrated knowledge translation (IKT)	7	• Integrated knowledge translation, knowledge transfer and exchange	• Collaborations; partnerships; engagement between researchers and research users, IKT, partnership between knowledge users and researchers, IKT partnership, research partnership, advisory council, family engagement, researcher–clinician partnership, engagement, knowledge user engagement, partnership	13
Other approaches	2 1 1	• Participatory implementation, embedded implementation research • Practice-based research network (PBRN) • Inclusive research	• Partnership, co-production, embedded research • Practice-based network	4

We identified several smaller approach clusters pertaining to participatory research [participatory action research (PAR), action research (AR), community-based participatory action research (CBPAR), and participatory evaluation] (17, 8%); patient and public engagement (13, 6%), community engaged research (CEnR or CER) (10, 5%), consumer involvement in research (9, 4%), community engagement (8, 4%), co-research (8, 4%), integrated knowledge translation (IKT) (7, 3%), and others [participatory and embedded implementation, practice-based research network (PBRN) and inclusive research] (4, 2%). The diversity of partnership approach descriptors further reveals a rich and broad set of approaches in the included literature (Table 3).

The complexity of and overlap in partnership approaches was further revealed when we examined key terms used to describe partnerships (Table 3). We collated unique key terms used by authors to describe health research partnerships and synthesized these by approach. As depicted in the unique terms column, there were 256 total terms used, with high overlap of terms between the 12 different approach domains. The coalition and partnerships domains contained the highest number of terms (50, 20% and 45, 18%, respectively), followed by participatory research (30, 12%) and patient and public involvement (24, 9%).

In almost half of included studies the initiating partner was researchers (74, 45%), followed by multi-stakeholder partnerships (16, 10%), and government departments, ministries and agencies (13, 8%) (Additional file 1: Appendix 7). The remaining partnerships were initiated by funders (6, 4%), not-for-profit organizations (4, 2%), foundations (3, 2%), community members and service users (2, 1% each), and clinicians and academic institutions (1, 1% each). In almost a third of included studies, the initiating partner was not reported (44, 27%). Of 260 reported partnership funding sources, government (including ministries, funding agencies, and departments) was by far the most frequent funder of health research partnerships (161, 62%), followed by non-profit organizations (25, 9%), foundations (22, 8%) and academic institutions (20, 8%). The remainder (16, 6%) were funded by endowments and healthcare organizations (5 each), industry (4), and regulatory bodies (2) (Table 3).

Importantly, 124 studies (75%) reported some level of co-production between researchers and partners in one or more phases of the research process.

### Tool evaluation criteria for included studies

**Fig. 3:**
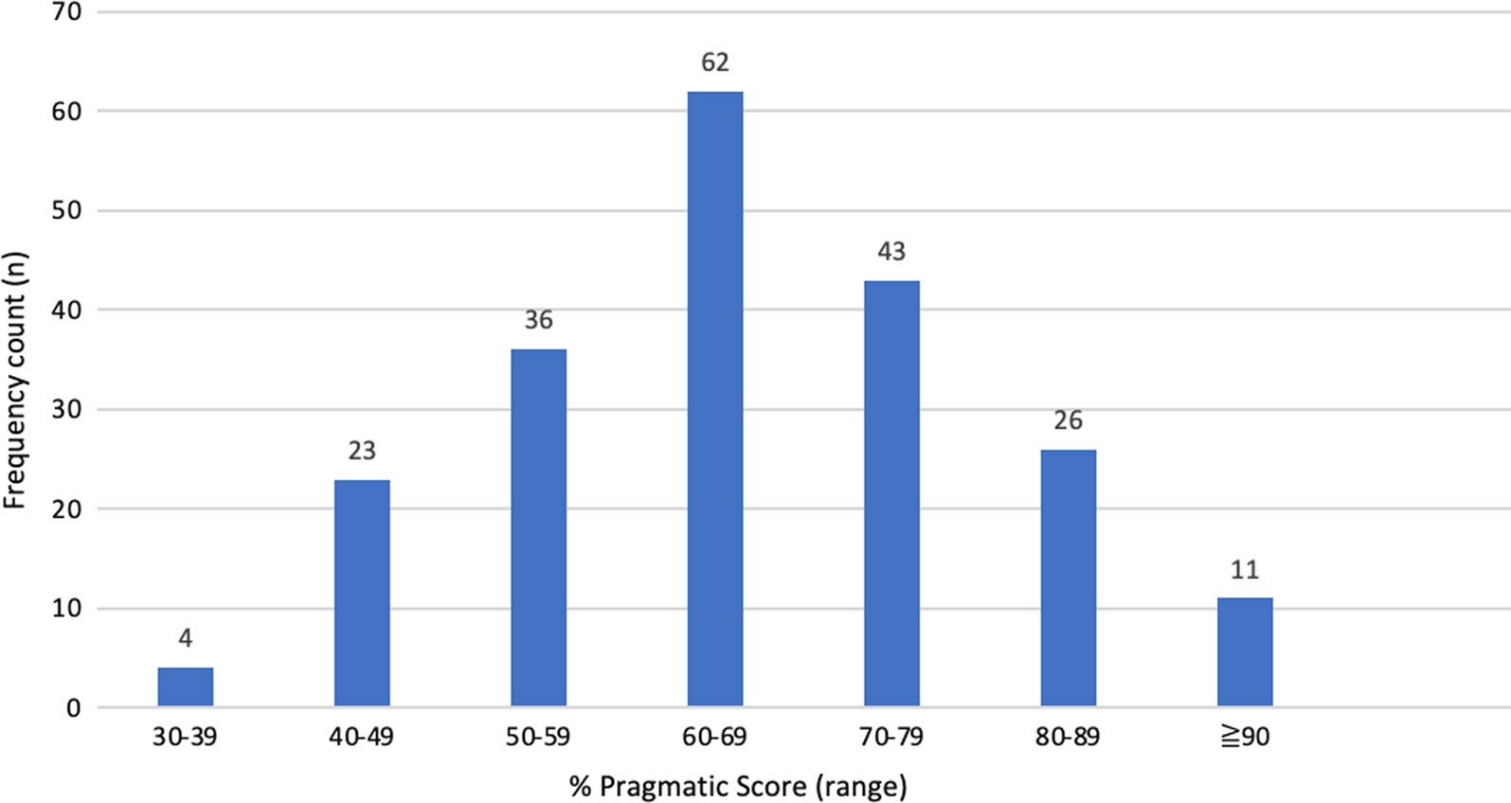
Health research partnership tool evaluation criteria scores (*n* = 205* tool scores). *Studies reporting multi-tools intended for simultaneous use were captured as toolkits and given a single, combined score

**Fig. 4:**
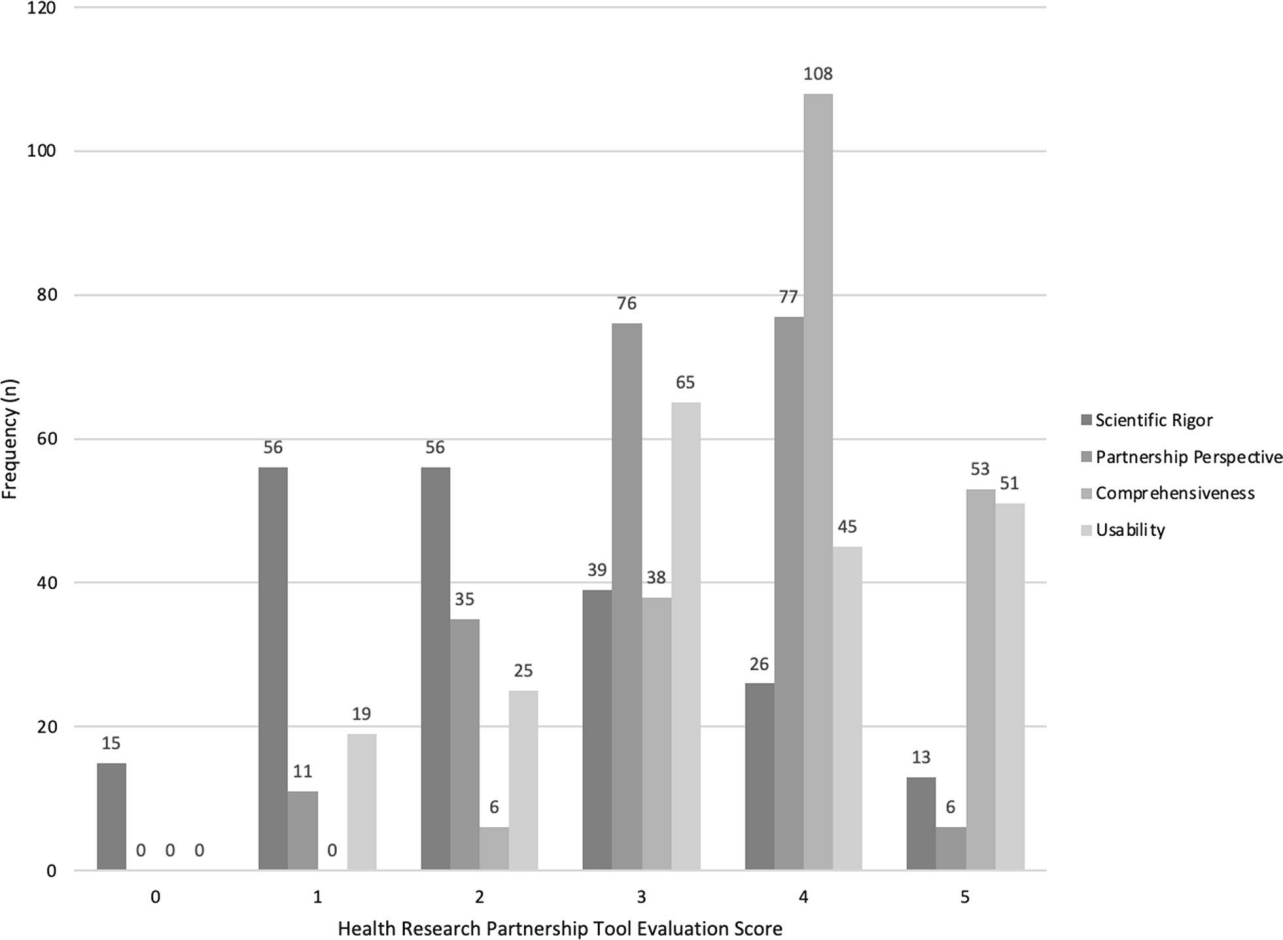
Health research partnership tool evaluation criteria scores, by domain

An inventory of tools and their domain and overall percentage scores is appended (Additional file 1: Appendix 8) on the basis of the modified, pragmatic health research partnership tool evaluation criteria (Additional file 1: Appendix 5). In total, we scored 205 tools, including 13 toolkits; the distribution of overall percentage pragmatic and of domain-specific scores is shown in Figs. 3 and 4. Mean domain scores were highest for tool comprehensiveness (4.01, SD 0.75), followed by tool usability (3.40, SD 1.25) and inclusion of the partner perspective (3.16, SD 0.93). The lowest mean domain score was for scientific rigor (2.21, SD 1.34). The mean overall tool score across all four domains, for the entire set of tools was 63.98% (SD 14.04).

### Synthesis of documented future research questions, evidence gaps and key recommendations

Most studies posed questions for future research, described evidence gaps and/or provided key recommendations related to outcomes and impacts assessment in their reports. We synthesized these, noting a high degree of overlap between future questions, evidence gaps and key recommendations, and hence, these findings were tabulated to facilitate their cross-referencing (i.e., study authors provided key recommendations that may help address some of the reported research questions and gaps). This aspect of the synthesis provides a rich series of research questions to guide the next steps in health research partnership assessment, tool development and partnership research in general (Additional file 1: Appendix 9).

Of the total number of reported research questions identified (325), a large number pertained to the further development and evolution of tools (80), including psychometric testing (30), tool testing (35) and tool and assessment process refinements and adaptations (11). The next most frequent type of research question pertained to partnership measurement and methods (46). A series of other research questions were identified, including the role of partnership in supporting sustainment (14), comparative effectiveness of partnership approaches (12), the use of theory (i.e., to guide evaluation, understand the influence of partnerships, expand and test conceptual frameworks and principles)(8), and questions pertaining to the evolution of partnerships over time, the role of leadership in partnerships (7, respectively), the role of context (6) and optimizing implementation and addressing priority population needs and concerns through partnership approaches (5, respectively). In sum, there is a significant overall call to address ‘how, how much, in whom, why (or why not) and under which circumstances’ questions for research partnerships to better understand how they develop, operate, achieve success and are best sustained.

Reported research gaps (Additional file 1: Appendix 10) were fewer in number but were closely aligned to the identified future research questions. The gaps comprised the need for objective metrics and for establishing conceptual underpinnings and structures supporting public and patient involvement. There was a single, sentinel reference regarding the need for advancing partnership research as a field (i.e., uncovering the contexts and mechanisms of engagement as a gateway to understanding impact), and one reference to health systems strengthening (i.e., the need to build capacity for systems thinking). Both questions align well with the general trend of using partnership to aid evidence uptake and use.

We also identified 54 key recommendations for the field of health research partnership outcomes and impacts assessment that may be helpful to investigators seeking direction for research questions and addressing gaps (Additional file 1: Appendix 11). Key recommendations included structural and other supports for research partnerships (26), sustainability planning (5), terminology (4), and for rigorous evaluation of partnerships (1).

Overall, we were able to identify multiple studies containing tools for the assessment of health research partnership outcomes and impacts in this scoping review [56]; a subset of these reported psychometric and pragmatic characteristics, hence we anticipate that a future systematic review on these tools and tool properties is feasible.

## Discussion

A synopsis of key findings from this large volume scoping review are outlined in Table 4. Briefly, we identified 166 unique papers and three companion reports containing 205 partnership assessment tools. Most studies were English language, originated in North America, were published after 2015 and were widely dispersed in the literature. Most studies were multi-purpose, featuring mainly mixed methods designs and the use of mixed methods. There were four main partnership approaches, and partnerships were primarily initiated by researchers and funded by government-funded departments, ministries, and funding agencies. Key terms were often interchanged and inconsistently defined and applied. Overall, identified tools were moderately comprehensive and usable, with lesser integration of partner perspectives. The scientific rigour of tools was low and few had evidence of psychometric testing. The focus of emerging research questions and recommendations was on tool evolvement and better understanding partnership measurement.

**Table 4:** Synopsis of key findings

Results section	Key findings
Study characteristics	• 166 studies, three companion reports • Widely dispersed literature, originating from North America, and published in English language after 2015 • Most studies were multi-purpose, with mixed methods designs and methods, and were guided by conceptual frameworks, models, theories • Challenges associated with terminology, definitions and their consistent application were observed • Few studies focussed on the evaluation or validation of tools
Tool characteristics	• 205 tools, most were surveys/questionnaires of self-reported perceptions; many tools with shared provenance • Most tools guided by conceptual frameworks, models, theories; few reported evidence of validity or reliability • Researchers, patients, and the public and community members were most common targets for tools
Partnership characteristics	• Most common partnership approaches were community based participatory research (CBPR), general partnership, patient and public involvement (PPI) and coalitions • Almost half were initiated by researchers, most involved some level of co-production in one or more study phases, and most were funded by government (ministries, research funders, departments)
Tool evaluation criteria findings	• Tools scored highest on comprehensive and usable domains, but scored lower on involving the partner perspective and lowest on scientific rigour • Overall, tool evaluation criteria scores were moderate
Future research questions	• Future research questions focussed on developing tools (psychometric and tool testing or refinement), partnership measurement and methods, engagement, revealing factors influencing partnership optimization and the optimization of partnership outcomes and impacts
Reported gaps	• Gaps comprised: Knowledge about engagement levels and timing Supporting research teams using partnership approaches Objective measures of partnership Structures to support patient and public involvement Some mention of gaps in the advancement of partnership research and health system strengthening
Reported recommendations	• Authors provided recommendations on: Structural and other supports for partnerships Engagement level and timing Sustainability planning Advancement of primary research for partnership approaches, terminology and rigorous evaluation

Overall, the findings suggest that the nature of this research domain and its tools are still nascent and actively evolving, as evidenced by high variation in terminology, concept definitions and their application. Numerous terms were frequently interchanged and mixed, obscuring the measurement and comparison of key concepts.

Our findings aligned well with other authors noting a lack of quantitative study designs and methods [28–31, 120] across multiple partnership approaches and populations. The number and diversity of solely quantitative designs and methods in our study was also low. However, as compared with earlier reviews [44, 49], mixed methods were more common. It is unclear whether the increased use of mixed methods designs and methods over earlier reviews [44, 49] reflects deliberate efforts to move beyond more traditional, qualitative evaluation approaches by integrating elements of quantitative partnership measurement (e.g., mixed methods approaches) in this field, or simply reflects a greater societal trend towards quantitative assessments and the pursuit of demonstrable, measurable impacts from research investments [121].

Our findings were also consistent with recommendations encouraging the development and use of objective measures (rather than proxy or self-reported measures) to assess partnership outcomes and impacts [28, 32, 33] to facilitate comparisons. Almost all included studies in this review involved self-reported measures of perception.

The location and language of the literature is explained by the geographic origins of partnership traditions and methods. High literature dispersion can be traced back to the independent evolvement of multiple health research partnership approaches over the past half century [3], and the lack of consolidation across partnership traditions [3].

The developmental state of partnership research and measurement is at least partly explained by studies’ purpose statements; most focussed on understanding and improving individual partnerships using fit-for-purpose tools. Only a small subset of studies had high scientific rigour domain scores, and few focussed specifically on tool development, testing, or evaluation. While these factors are at least partly a function of the complexities of partnership assessment, the challenges associated with tool development cannot be understated [122].

The development of high quality, psychometrically and pragmatically robust tools is a function of unique resource, time and expertise demands of tool development [122]. These requirements are often underestimated, and lack of attention to tool development requirements can slow scientific measurement and innovation [122]. Based on our synthesis of future research questions, existing knowledge gaps and recommendations, a focus on measurement, methods and tool development, testing and refinement is considered a necessary next step in advancing the field.

Despite differences in review scope (e.g., populations, partnership traditions, databases, search terminology, effects), our findings were similar to other reviews on broad issues related to diverse terminology, location, accessibility of tools and publication dispersion in the health research partnership domain [13, 28, 29, 33, 49, 123]. However, more detailed comparisons with these and other existing reviews directly related to partnership assessment tools and their characteristics revealed complexities. We found only a 5%–50% overlap of identified tools when we compared our findings with pre-existing reviews pertaining to: (a) patient and public involvement evaluation tools (6 of 27 tools overlapped with our study, 22%) [28], (b) an overview of reviews pertaining to research co-production impact assessment tools (4 of 75 tools overlapped with our study, 5%) [29], (c) a review of CBPR process and outcome measurement tools (14 of 46 tools overlapped with our study, 30%) [49], (d) a review of success in long-standing CBPR partnerships (tools in 3 of 16 relevant partnerships overlapped with our review, 19%) [51] and (e) a review of the organizational participatory research (OPR) health partnerships (three of six tools overlapped with our review, 50%) [50]. In the tools we identified in our review, only 30 (of a possible 170, 18%) overlapped with these other reviews.

In each case, the lack of overlap can be accounted for by fundamental differences in the partnership concept with linked search terms and scope (e.g., breadth of literature, search time frame, inclusion of research domains beyond health, and different measured effects).

More specifically, Boivin and colleagues’ review [28] differed in its limitation to patient/public-focussed evaluation tools for assessing engagement in health system decision making and health research. It employed narrower search terms over a shorter frame (1980–2016), but accessed an additional database (Cochrane Database of Systematic Reviews) and grey literature (Google) sources [28]. The MacGregor overview of reviews examined impacts, but also differed by time frame, key partnership terminology and domain scope. Seven of eight included reviews were published since 2015, four of these were out of scope, and only 17.2% of the primary studies were published since 2010 (in our review, 55% of the primary literature was published after 2015). Sandoval and colleagues’ review used a broader database set and grey literature (PubMed, SciSearch, SocioFile, Business Source Premier, PsycINFO, Communication and Mass Media Complete and a Google key term search). Brush and colleagues’ review [51] identified studies and tools used to evaluate partnerships on a more limited time span (2007–2017) and was limited to CBPR terms and used different databases (PubMed, Scopus, CINAHL). Finally, Hamzeh and colleagues’ review [50] identified three (of 6, 50%) overlapping tools using comprehensive OPR search terms, a broader database scope and multiple bibliographic and grey literature sources.

In each case, subtle differences in partnership terminology and scope generated very different results—and very little overlap with the tools we identified in our review. Nonetheless, comparisons with these other reviews revealed a multitude of partnership assessment tools, albeit variably defined, in this research domain. It was noteworthy that despite these clear differences in terminology and scope, several key, overarching messages were recurrent and similar: (a) there is a need to advance quantitative measurement, tool development and psychometric and pragmatic tool testing, and (b) there is a need to better understand partnerships, and how to monitor, measure and optimize them and their outcomes and impacts. In our review, these priorities were further evidenced in the partnership tool development and measurement and partnership themes gleaned from our synthesis of reported research questions, evidence gaps and key recommendations, combined (Additional file 1: Appendices 9–11). Authors of studies included in our review identified the need to raise awareness, develop knowledge and competency in partnership working, establish clear terminology and definitions, and to advance specific roles for researchers, funders and partnership stakeholders to support partnership establishment, maintenance, measurement and sustainment. These priorities align well with calls for dedicated investment to systematically and rigorously measure partnership outcomes and impacts [12, 124–127].

In sum, there is increased use and prominence of partnership approaches as a mechanism to achieve more user-relevant outcomes and impacts. In this way, partnership approaches are particularly relevant in the field of knowledge translation and implementation sciences [1, 7, 24, 25, 33, 125, 128–131]. Addressing the aforementioned and fundamental issues related to partnership conceptualization, measurement and optimization will be required for the overall advancement of the field of partnership research and its application.

### Strengths and limitations

This review is unique in its attempt to locate literature and health research partnership outcomes and impacts assessment tools spanning multiple health research partnership approaches and partners, in varied contexts, within the health domain. To our knowledge, this is the largest review of its kind, traversing multiple traditions and partner groups in the health research partnerships domain. Uniquely, our review strategy employed terms spanning multiple research partnership approaches and partner types, from database inception, and without restrictions (e.g., by study design, language, research domain or time frame). We followed strict methodological protocols at each review stage and generated detailed assessments of tool and partnership characteristics that can assist researchers in choosing, applying and considering testing and refining tools.

The location and retrieval of relevant literature and tools in this review was limited by documented challenges relating to locating literature in multiple research partnership traditions, diverse and inconsistent terminology, literature dispersion and journal limits (e.g., space limits, lack of open access and appendices for tools). We attempted to mitigate these challenges by using a pre-tested and inclusive terminology catchment for key search terms, by searching four key databases from inception, and by making at least two attempts to reach investigators and locate tools. A significant number of inquiries went unanswered or bounced back; tools were generally unavailable from publication files, there was high non-response to emails, and many tools were unavailable, even upon researcher contact. As other authors attest, tool accessibility remains problematic [28] and may preclude tool use in this research domain.

Another limitation of this review was the lack of detail pertaining to the assessment of the health research partnerships present in published abstracts and full text reports. We purposefully retained studies for full text review if their eligibility was uncertain due to ambiguity in the title/abstract screening phase but note the burden of this approach in a large evidence review. Despite this effort, a general lack of evaluative detail regarding health research partnerships persisted in the full text articles. Furthermore, when health research partnership and tool assessment outcomes occurred as secondary (or as inexplicit) research objectives in published reports, reporting detail was frequently lacking, exacerbating abstraction challenges. Also, studies were often multi-purpose, mixing multiple methods. While beneficial for research purposes, this posed challenges for data abstraction because the degree to which mixed methods were integrated in the results varied greatly. At times, this made differentiating partnership, tool and tool assessment findings challenging.

### Future research

There is a need for research into both the measurement and the partnership approach facets of this growing research field. First, it is important to recognize that measurement is a key precursor to advancing partnership research and partnership measurement research. The combined complexity of partnership assessment and tool development will require dedicated resources, time spans and researcher expertise that will need to be built [122]. Given the number of existing tools, future research should focus on both the psychometric and pragmatic testing of fit-for-purpose and other tools and/or their components in different contexts. The diversity of approaches, and the volume and variable quality of tools in this literature offers significant potential to consolidate, share, apply, test and compare knowledge of partnerships and partnership measurement across traditions. Consensus building and ongoing dialogue to compare and contrast the different approaches, terminologies and definitions will be important next steps, as reflected by our synopses (Additional file 1: Appendices 9–11). It is unclear whether partnerships vary in distinct ways (e.g., by partner, partnership type, context and/or partnership tradition) that necessitate different (and/or fit-for-purpose) tools or tool components or whether standardized tools can be feasibly developed and applied; this is a key area of future research. Finally, our understanding of the effects of health research partnerships is nascent and will require focussed measurement and adequate evaluation time spans to optimize health research partnerships, assessment measures and their outcomes and impacts.

## Conclusions

This large volume scoping review extends our understanding of the characteristics, types and accessibility of tools to assess the outcomes and impacts of health research partnerships. Not many of the identified tools overlapped with those identified in previous reviews, but their characteristics were similar in that most were tailored for specific partnerships and lacked scientific rigour and evidence of psychometric testing. Our synthesis of tool, tool evaluation and partnership characteristics confirmed the need for dedicated efforts and resources to study health research partnerships and their systematic evaluation using valid, reliable and pragmatic tools that meet partner needs. Investing in research to better understand research partnership outcomes and impacts measurement remains a key priority for this field.

### Scoping review and coordinated multicentre team protocol registrations

1. Open Science Framework (Scoping Review Protocol): https://osf.io/j7cxd/
2. Open Science Framework (Coordinated Multicentre Team Protocol): https://osf.io/gvr7y/
3. Coordinated Multicenter Team Protocol Publication: https://systematicreviewsjournal.biomedcentral.com/articles/10.1186/s13643-018-0879-2

### Supplementary Information

**Additional file 1: Appendix 1.** Scoping review data map. **Appendix 2.** Protocol deviations and rationale. **Appendix 3.** Expanded methods. **Appendix 4.** Search strategy. **Appendix 5.** Health research partnership tool evaluation criteria. **Appendix 6.** Year of publication for included studies. **Appendix 7.** Partnership characteristics. **Appendix 8.** Pragmatic health research partnership criteria assessments. **Appendix 9.** Synthesis of future research questions. **Appendix 10.** Synthesis of evidence gaps. **Appendix 11.** Synthesis of recommendations. **Appendix 12.** Bibliography of included studies. **Appendix 13.** PRISMA-Scoping Reviewschecklist, references.

**Additional file 2: Table S1.** Characteristics of included studies, **Table S2.** Tool characteristics

## Data Availability

The study search strategy, abstraction tools and bibliographic tool index will be available through the Open Science Framework upon completion of the research and publication of findings. Data generated and/or analysed during the current study will be made available upon reasonable request from the author, after completion of the dissertation research and publication of findings.
