# Supplementary material for: A scoping review of the globally available tools for assessing health research partnership outcomes and impacts
Source: Health Res Policy Syst. 2023 Dec 22;21:139. doi: 10.1186/s12961-023-00958-y (PMC10740226; doi:10.1186/s12961-023-00958-y)
Supplement: Supplementary file 1 — Additional file 1: Appendix 1. Scoping review data map. Appendix 2. Protocol deviations and rationale. Appendix 3. Expanded methods. Appendix 4. Search strategy. Appendix 5. Health research partnership tool evaluation criteria. Appendix 6. Year of publication for included studies. Appendix 7. Partnership characteristics. Appendix 8. Pragmatic health research partnership criteria assessments. Appendix 9. Synthesis of future research questions. Appendix 10. Synthesis of evidence gaps. Appendix 11. Synthesis of recommendations. Appendix 12. Bibliography of included studies. Appendix 13. PRISMA-Scoping Reviewschecklist, references. [file 12961_2023_958_MOESM1_ESM.docx]

Additional File 1

[**Appendix 1** Scoping Review Data Map 2](#_Toc108650508)

[**Appendix 2** Protocol Deviations and Rationale 3](#_Toc108650509)

[**Appendix 3** Expanded Methods 6](#_Toc108650510)

[**Appendix 4** Search Strategy (Ovid - MEDLINE) 13](#_Toc108650511)

[**Appendix 5** Health Research Partnership Tool Evaluation Criteria 18](#_Toc108650512)

[**Appendix 6** Year of Publication for Included Studies 21](#_Toc108650513)

[**Appendix 7** Partnership Characteristics 22](#_Toc108650514)

[**Appendix 8** Pragmatic Health Research Partnership Criteria Assessments 50](#_Toc108650515)

[**Appendix 9** Synthesis of Future Research Questions 74](#_Toc108650516)

[**Appendix 10** Synthesis of Evidence Gaps 84](#_Toc108650517)

[**Appendix 11** Synthesis of Recommendations 84](#_Toc108650518)

[**Appendix 12** Bibliography of included studies 87](#_Toc108650519)

[**Appendix 13** PRISMA- ScR Checklist 98](#_Toc108650520)

[**References** 101](#_Toc108650521)

# **Appendix 1** Scoping Review Data Map

| **Key Resources** | **Description** | **Location** |
| --- | --- | --- |
| Figure 1 | Scoping review PRISMA study flow diagram | Main manuscript |
| Figure 2 | Health sub-domains and key sub-domain clusters | Main manuscript |
| Figure 3 | Health research partnership tool evaluation criteria scores | Main manuscript |
| Figure 4 | Health research partnership tool evaluation criteria scores, by domain | Main manuscript |
| Table 1 | Key terms and definitions | Main manuscript |
| Table 2 | Inclusion-exclusion criteria | Main manuscript |
| Table 3 | Characteristics of included studies | Main manuscript |
| Table 4 | Tool Characteristics | Main manuscript |
| Table 5 | Key partnership approaches and partnership terms | Main manuscript |
| Table 6 | Synopsis of key findings | Main manuscript |
| Appendix 1 | Scoping Review Data Map | Additional File |
| Appendix 2 | Protocol deviations and rationale | Additional File |
| Appendix 3 | Expanded methods | Additional File |
| Appendix 4 | Search strategy (OVID – Medline) | Additional File |
| Appendix 5 | Health research partnership tool evaluation criteria | Additional File |
| Appendix 6 | Year of publication of included studies | Additional File |
| Appendix 7 | Partnership characteristics | Additional File |
| Appendix 8 | Pragmatic health research partnership criteria assessments, by domain and total score | Additional File |
| Appendix 9 | Synthesis of future research questions | Additional File |
| Appendix 10 | Synthesis of evidence gaps | Additional File |
| Appendix 11 | Synthesis of recommendations | Additional File |
| Appendix 12 | Bibliography of included studies | Additional File |
| Appendix 13 | PRISMA-ScR checklist | Additional File |

# **Appendix 2** Protocol Deviations and Rationale

| **Protocol Deviation** | **Rationale** |
| --- | --- |
| **Electronic databases (scope cost-benefit)** | The initial search strategy included both Scopus and Web of Science databases, however, we chose to exclude both databases after conducting the scout search to narrow the strategy further and focus on health- (versus bench science) related citations. Both databases were excluded prior to PRESS review[1, 2]. |
| **Grey literature and referral literature (scope cost-benefit)** | Retrieval of grey literature and referral literature were removed. High quality tools identified from grey literature and referrals were often replicated by peer-reviewed publications and on balance, the benefits to costs in terms of literature volume and feasibility were low, hence these Additionalal strategies were discarded. |
| **Duplicate, Independent Abstraction (feasibility, efficiency)** | After pilot testing full text abstraction on a data subset, we ascertained that duplicate, independent abstraction was not feasible due to a) the complexity of the data (highly variable levels of detail), 2) type of abstractable data (qualitative), requiring a more flexible abstraction strategy. The independent duplicate abstraction strategy was replaced with independent abstraction with secondary, independent validation, and all arising discrepancies resolved to consensus. This approach was a better, more efficient fit for the type of data abstraction and nature of the data involved. |
| **Key Terms and Definitions**  **(Recognition of past harms and reconciliation, alignment with source data, inclusivity)** | Historic harms are propagated through language[3]. In the spirit of recognizing and reconciling such harms, we have replaced the word *stakeholder(s)* with the word *partner(s)* throughout the manuscript.  The terms Majority and Minority country were used to indicate locations where the respective majority or minority of the global populace resides (historically, the terms ‘developing’ and ‘developed’ nations were used, but these terms have negative connotations and are outdated)[4].  Definitions for the term *outcome* was altered to be more inclusive (e.g., “factor(s) described in the study methods used to determine a change in status as a result of interventions, can be measured or assessed as component(s) of the study, and are not futuristic”; including both process and summative outcomes.) (Adapted from Hoekstra et al, 2018 and University of Waterloo, 2018)[5, 6].  We also modified the definition of *impact* to capture broader positive and negative research partnership implications (“effects, influences or changes to the economy, society, public policy or services, individuals, teams, organizations, health, the environment, or quality of life, beyond academia”) (Adapted from Hoekstra et al, 2018 and Higher Education Funding Council for England)[5, 7]  The definition of *tool* was altered to reflect the distinct content of citations related to indicators and metrics. *Indicators* and *metrics* were removed from the definition and the papers excluded. These citations were flagged and retained as a separate data subset so that further examination and synthesis could be undertaken in a separate publication. |
| **Tool Evaluation Criteria**  **(Response to source data)** | After pilot-testing the PAPERS psychometric and pragmatic (partner facing and objective) criteria[8, 9] with a subset of eligible studies, it was clear that the level and type of detail required to apply them comprehensively across the sample was lacking, as evidenced by the high number of incomplete or missing data cells and challenges finding relevant data in reports.  Thus, we replaced both the PAPERS psychometric and pragmatic criteria [8] and the Global Assessment/Referral Criteria[10] with a modified version of consensus-built scientific and pragmatic criteria developed by the Centre of Excellence on Partnership with Patients and Public (CEPPP)[11], and used by Boivin and colleagues in a systematic review[12].  These pragmatic, user-friendly criteria were developed and written by- and for a diverse set of interested parties (e.g., patients, public, clinicians, decision makers, community members, researchers, among others), as part of the Patient and Public Engagement Evaluation Toolkit Project[11]. In consultation with the CEPPP (A L’Esperance), our team crafted, successfully pilot-tested and then systematically applied a modified version of the CEPPP criteria to eligible studies. |
| **Abstracted Variables**  **(Response to source data, reporting scope)** | Study Characteristics Variables: [author, year, title, language, country, health sub-domain, focus (discipline), population targeted by tool, study type, study design, study method(s), sex (tool targets), research questions, research/evidence gaps, recommendations].  Variables reported elsewhere: [lessons, challenges, benefits, antagony, advantages, disadvantages, sustainability, ethics, key conclusions].  Unavailable: [number study participants: this variable was not reliably reported to enable systematic abstraction]  Tool Characteristics: [tool name, tool type, tool format, tool purpose, number of tool items, target user type/target user(s), intended respondent type, underlying theory/model/framework].  Reported elsewhere: [tool constructs/categories, definitions]  Partnership Characteristics:  [partnership term(s) and definitions, form of partnership, partnership funding, initiating partner, partnership members, partnership purpose, co-production (presence)].  Reported elsewhere: [co-production definition]; Unavailable: [co-production value: not systematically reported] |

# **Appendix 3** Expanded Methods

*Approach*

This research is part of an ongoing Integrated Knowledge Translation Research Network[13] effort to consolidate the health research partnership evidence base and improve our understanding of the characteristics associated with health research partnerships[5, 13-17]. Where possible, our review strategy was pragmatic to balance and accommodate the documented challenges of literature dispersion, terminology, accessibility, and feasibility limitations[5, 14, 18-20], while: a) remaining focused on consolidating evidence across research partnership traditions, b) balancing search breadth against citation volume and review feasibility, and c) optimizing the identification and retrieval of accessible tools and their key characteristics[5, 14, 15, 20, 21]. To do this, we followed the six-step, iterative method outlined by Arksey and O’Malley[22] and its subsequent refinements [23-25], and followed scoping review guidelines, methods and review reporting standards [26-31]. A skeleton protocol, itemized PRISMA-P checklist, and operational terms and definitions were published *a priori* as part of the broader network approach[5].

*Search Strategy and Data Sources Methods*

An *a priori* search strategy was developed using pre-selected source papers that described collaborative health research partnership approaches and employed tools to assess partnership outcomes and impacts. The strategy was assembled from relevant keywords, publication indexing, and Medical Subject Headings (MeSH). In consultation with a medical research librarian (MVD), the draft strategy was structured and iteratively audited[32] to refine its feasibility and sensitivity in capturing pre-selected and related works. These health research partnership term clusters and search strategy development methods underlie several parallel health research partnership syntheses[5, 14, 15]. We used MEDLINE (OVID) to test search strategy feasibility, and the degree to which it successfully identified pre-selected papers[33]. This process confirmed previously reported complexities, terminology, and dispersion issues in the health research partnerships outcomes and impacts assessment literature. Ultimately, terms within three search themes (partnership, impact, and measurement properties) were combined using Boolean syntax (“OR”) and subsequently combined (“AND”). Adjacency parameters (e.g., researcher adj2 partnership*; community adj3 research*, and participatory adj2 research*) and wildcard symbols (*) were utilized for several keywords to refine the strategy and increase relevant hits. We subjected the partnerships theme to the Peer Review of Electronic Search Strategies (PRESS) checklist[1, 2] review by an academic librarian (CN) and the overall search strategy was later subjected to PRESS review to improve quality, comprehensiveness, and further optimize the balance between search sensitivity and scope feasibility. Suggestions arising from each review involved new terms; however, each had previously been tested and discarded during the search preparatory phase. We finalized the MEDLINE (Ovid) strategy with a single term spelling correction (MeSH term ‘benchmarking’)[34]. No restrictions on date, study design, data type or language were applied. Grey literature was initially proposed, but later disregarded after surveying the available literature during piloting of the strategy and its subsequent refinement. We undertook systematic de-duplication of the total citations retrieved [28, 56] using the Bramer protocol[35] in Endnote**™** X7.8.

*Expanded Definitions and Operationalized Terms*

To best reflect study geographic origins, we categorized studies using the terms majority or minority country to indicate locations where the respective majority or minority of the global populace resides (historically, these terms were referred to as ‘developing’ and ‘developed’ nations).

*Expanded Eligibility and Screening*

We independently screened titles/abstracts and eligible full text in duplicate (KJM with team members: JMB, LP, LN, SS, SM, CM, LS, AG, KA) using a citation database and screening spreadsheets in MS Excel[36]. We undertook pilot-testing and revision of screening tools[37, 38] applying a minimum limit (Cohen’s κ ≧0.60)[39] to calibrate screening at each review stage and prior to commencement, per large volume evidence review best practice guidelines[40]. Data was abstracted from eligible full text studies using a hybrid approach. The hybrid abstraction approach struck an acceptable balance[23] between literature volume, data complexity, variability in content arising from the qualitative data cells[41] and efficient resource use. We retained studies describing a health research partnership and the development, use, and/or assessment of a health research partnership outcome or impact assessment tool (or element of-, or at least one health research partnership outcome or impact measurement property[21, 42] of a tool), as an aim of the study.

Studies describing researcher partnerships involving evaluative, process evaluative, technical assistance, and facilitated implementation-type research activity or roles were systematically retained. While these studies likely reflected a wide range of researcher partnerships, they lacked the level of detail required to discern the exact degree of partnership and were therefore retained because they met our review inclusion-exclusion criteria. Feasibility challenges (e.g., incorrect author contacts, inability to locate and access tools, time/resource demands) further precluded refined assessment of the degree to which researchers were partnered in such studies; this is a recognized limitation of both the reported literature and this review. We determined that studies reporting indicators and metrics were qualitatively distinct from other eligible tool studies; therefore, these studies were excluded, but flagged and retained as a discrete data subset for future synthesis. Health research partnerships in which the main aim was the recruitment/retention of research participants alone were also systematically excluded.

We considered, but did not retain any conference abstracts arising from full text review, after confirming their status as preliminary or duplicate reports, or lacking in sufficient data for abstraction[43]. We encountered difficulties locating and accessing tools arising from abstracts. Protocols (n=2) were retained as companion reports to Additional a superseding, primary research report. Reviews of any type were flagged at title/abstract and full text screening; these reports were retained separately for the purposes of informing the overall study.

Finally, there exists high fluidity in the way outcomes and impacts are distinguished, defined, and reported in the literature[14, 20]; therefore, the review included studies referring to outcomes and impacts assessment using multiple terms (e.g., including but not limited to process, proximal, immediate, intermediate, summative, long-term outcomes and/or proximal, immediate, intermediate, distal, or long-term impacts). Studies that met all inclusion criteria were retrieved for full text review.

*Expanded Abstraction Methods*

All title, abstract and full text screening was undertaken independently and in duplicate by KJM with team members (JB, LP, LN, SS, SM, CM, LS, AG) using a citation database and screening spreadsheets in MS Excel[36]. WE undertook pilot-testing and revision of screening tools [37, 38], and applied a minimum calibration limit (Cohen’s κ ≧ 0.60)[39] at each review phase, prior to commencement, per large-evidence review best practice guidelines[40]. This helped ensure reviewers’ understanding of the study aims and eligibility criteria[44]. At each review phase, discrepancies were resolved by consensus before passing citations on. We used a hybrid strategy involving independent abstraction (KJM) and independent validation by a second, trained investigator (MK, SS, SM) in the data abstraction phase[26], with all discrepancies resolved with consensus by dual review and discussion at weekly meetings, and guided by a pilot-tested tool and coding manual[22, 37, 38]. This hybrid abstraction approach struck an acceptable balance[23] between literature volume, data complexity, variability in content arising from the qualitative data cells[41] and efficient resource use. To minimize the effects of tool and author inaccessibility[5, 14, 21], we made at least two attempts to locate authors and/or tools for each study, including web searches to update or locate incorrect corresponding author emails, contacting co-authors, library and web searches, and organizational and/or funder requests to locate investigators and tools. Feasibility pressures precluded our ability to contact authors for missing information, however, we successfully contacted all authors to clarify queries regarding any apparent duplicate and/or overlapping reports[40]. When companion reports were identified, data was combined with the superseding or pre-existing tool study to create a single, comprehensive abstraction. Authors were contacted only to locate tools, or to clarify duplicate and/or overlapping reports[40].

All foreign language studies and tools were translated prior to abstraction. We abstracted variables pertaining to study characteristics, tool characteristics, partnership characteristics and tool evaluation characteristics, as outlined in the aforementioned detailed protocol[45]. Protocol modifications and rationale are itemized in the Abstracted Variables section (Appendix 1, ADDITIONAL).

*Expanded Tool Evaluation Criteria*

During protocol development, we reviewed several sets of tool assessment criteria to examine psychometric and pragmatic criteria (e.g., COSMIN Criteria[42, 46], The Standards for Educational and Psychological Testing[47]). We chose the evidence-based, consensus built pragmatic PAPERS criteria[8, 9] comprising both psychometric and pragmatic (stakeholder facing and objective) components. As other authors have reported[48], we encountered challenges during piloting related to relevance, overall fit with the level and quality of available detail amenable to scoring, and the comprehensibility of scores for a broad stakeholder audience. Ultimately, we adapted consensus-built criteria developed by Boivin and colleagues as part of the Patient and Public Engagement Evaluation Toolkit Project[11, 12]. The criteria were developed to facilitate an easily interpretable assessment of tools for evaluating patient and public engagement[11]. These criteria aligned more closely with the literature we were assessing because they: a) were comprehensive (covered similar scientific and pragmatic (stakeholder-facing and objective) domains at a less-detailed level that better matched reports); b) were the most pragmatic criteria we could locate in terms of tool characteristics and assessment in the health research domain; c) were consistently and easily applicable to pilot papers with minimal missing data points; and d) produced a comprehensible set of intuitive scores that were easy to understand and interpret by a diverse stakeholder audience. The criteria were developed to facilitate an easily interpretable assessment of tools for evaluating patient and public engagement[11], and were not intended for use as a formal quality assessment[12].

In consultation with the Centre on Patient and Public Participation (CEPPP, A L’Esperance), the team modified the assessment criteria in three rounds, piloting, modifying, and discussing the items iteratively, to consensus. The final set of 20 modified criteria and companion scoring rubric closely mirrored the original version (e.g., 5 questions in each of 4 domains: Scientific Rigor, Stakeholder Perspective, Comprehensiveness, Usability). In cases where > 1 tool was reported and intended for combined use, we categorized these as toolkits and scored them as a single tool. Guided by clinical audit conventions[49, 50], we used a minimum random sample to validate pragmatic criteria ratings and minimize reviewer bias and errors[51]. In 111 (67%) of included studies, we identified only 28 of 2220 total cells in 19 studies requiring amendment, a ~1.3% error rate. Corrections stemmed from reporting clarity and clustered around reported evidence use, use of frameworks/models, and 3 tool characteristics (tool design for reporting back to partners, monitoring, and inclusion of open-/closed questions). Discrepancies arising in this process were resolved to consensus.

Per convention, we did not undertake methodological quality appraisal and risk of bias for this scoping review[28, 52, 53].

*Expanded Analysis Methods*

For health sub-domains, we used key words and study purpose statements to code sub-domain(s), allowing for ≧ 1 code/study, where necessary. We took a similar approach to code health research approaches because studies frequently described mixed-approach activities (for example, a coalition using a community based participatory research (CBPR) approach).

# **Appendix 4** Search Strategy (Ovid - MEDLINE)

| Database: "Ovid MEDLINE(R) Epub Ahead of Print, In-Process & Other Non-Indexed Citations, Ovid MEDLINE(R) Daily and Ovid MEDLINE(R) 1946 to Present” | |
| --- | --- |
| Line | Search terms |
| 1 | ("community partners" adj2 (research* or engage*)).ab,ti |
| 2 | ("as partners" adj4 research).ab,ti |
| 3 | ("community engagement" AND research).ab,ti |
| 4 | ("community involvement" AND research).ab,ti |
| 5 | ("community participation" adj3 research).ab,ti |
| 6 | ("knowledge users" adj3 research).ab,ti |
| 7 | ("service user" AND (involvement OR engagement)).ab,ti |
| 8 | ((clinical or health) adj4 partnership*).ab,ti |
| 9 | (collaborat* adj3 (partner* or research*)).ab,ti |
| 10 | ((engag* or participation) adj2 stakeholder?).ab,ti |
| 11 | ((involvement or engagement) adj4 research).ab,ti |
| 12 | (partnership* adj4 research*).ab,ti |
| 13 | (action research).ab,ti |
| 14 | (coalition? adj3 health).ab,ti |
| 15 | (cocreation).ab,ti |
| 16 | (co-creation).ab,ti |
| 17 | (community coalition?).ab,ti |
| 18 | (community-based research).ab,ti |
| 19 | (community-engaged research).ab,ti |
| 20 | (consumer involvement).ab,ti |
| 21 | (coproduction AND research).ab,ti |
| 22 | (co-production AND research).ab,ti |
| 23 | (coresearch*).ab,ti |
| 24 | (co-research*).ab,ti |
| 25 | (disseminat* research).ab,ti |
| 26 | (emancipatory research).ab,ti |
| 27 | (engage* adj3 research*).ab,ti |
| 28 | (engaged scholarship).ab,ti |
| 29 | (inclusive adj2 research).ab,ti |
| 30 | ("knowledge transfer and exchange").ab,ti |
| 31 | (knowledge translation).ab,ti |
| 32 | (participatory design).ab,ti |
| 33 | (participatory evaluation).ab,ti |
| 34 | (participatory intervention?).ab,ti |
| 35 | (participatory research).ab,ti |
| 36 | ("patient and public involvement").ab,ti |
| 37 | (patient-centered adj2 research).ab,ti |
| 38 | (peer research).ab,ti |
| 39 | (research adj2 translation).ab,ti |
| 40 | (("mode 2" or "mode two" or "mode II") adj3 (knowledge or research)).ab,ti |
| 41 | ("linkage and exchange").ab,ti |
| 42 | (research and ("peer led" or "public led" or "patient led" or "stakeholder led")).ab,ti |
| 43 | 1 or 2 or 3 or 4 or 5 or 6 or 7 or 8 or 9 or 10 or 11 or 12 or 13 or 14 or 15 or 16 or 17 or 18 or 19 or 20 or 21 or 22 or 23 or 24 or 25 or 26 or 27 or 28 or 29 or 30 or 31 or 32 or 33 or 34 or 35 or 36 or 37 or 38 or 39 or 40 or 41 or 42 |
| 44 | (community-based participatory research).kw,kf |
| 45 | (participatory research).kw,kf |
| 46 | (integrated knowledge translation).kw,kf |
| 47 | (action research).kw,kf |
| 48 | (community involvement).kw,kf |
| 49 | (participatory action research).kw,kf |
| 50 | ("patient and public involvement").kw,kf |
| 51 | (cocreation).kw,kf |
| 52 | (co-creation).kw,kf |
| 53 | (Collaborative research).kw,kf |
| 54 | (Community engagement).kw,kf |
| 55 | (community-academic partnership).kw,kf |
| 56 | (community-based research).kw,kf |
| 57 | (coproduction).kw,kf |
| 58 | (co-production).kw,kf |
| 59 | (Inclusive research).kw,kf |
| 60 | (knowledge exchange).kw,kf |
| 61 | (participatory research partnership?).kw,kf |
| 62 | (partnership research).kw,kf |
| 63 | (Patient participation).kw,kf |
| 64 | (researcher-stakeholder collaboration).kw,kf |
| 65 | 44 or 45 or 46 or 47 or 48 or 49 or 50 or 51 or 52 or 53 or 54 or 55 or 56 or 57 or 58 or 59 or 60 or 61 or 62 or 63 or 64 |
| 66 | 43 or 65 |
| 67 | ((outcome? OR impact?) AND (measur* OR assess* OR evaluat*)).ab,ti |
| 68 | ((outcome? or impact?) AND (tool? OR instrument?)).ab,ti |
| 69 | ((measur* OR assess* OR evaluat*) AND (tool? OR instrument?)).ab,ti |
| 70 | 67 or 68 or 69 |
| 71 | Benchmarking/ |
| 72 | "Surveys and Questionnaires"/ |
| 73 | Program Evaluation/ |
| 74 | "Outcome Assessment (Health Care)"/ |
| 75 | "Outcome and Process Assessment (Health Care)"/ |
| 76 | "Process Assessment (Health Care)"/ |
| 77 | Health Impact Assessment/ |
| 78 | Reproducibility of Results/ |
| 79 | Psychometrics/ |
| 80 | 71 or 72 or 73 or 74 or 75 or 76 or 77 or 78 or 79 |
| 81 | 70 or 80 |
| 82 | 65 and 81 |

# **Appendix 5** Health Research Partnership Tool Evaluation Criteria

**Health Research Partnership Tool Pragmatic Assessment: Instructions, Criteria and Assessment Grid** **(***Modified from the Patient and Public Engagement Evaluation Toolkit Project)* *(Boivin et al, 2018)*

Four criteria domains are defined as follows with a five-star rating generated for each:

- **Scientific Rigor:** Was the development of the tool scientifically rigorous and based on existing evidence pertaining to health research partnership outcome and impact assessment?
- **Partner Perspective:** Does the tool take into account partner views in its development/modification, use, evaluation and/or validation?
- **Comprehensiveness:** Does the tool comprehensively assess the context, process, outcomes and/or impacts of health research partnerships?
- **Usability:** Is the tool easy to use?

Each criterion has 5 question items. Score 1 point per item if the answer is YES, score 0 if the answer is NO or CANNOT ANSWER.

|  | **Question** | **Notes** |
| --- | --- | --- |
| **Scientific Rigor (SR)** | | |
| SR1 | Is the tool based on a comprehensive literature review on the assessment of outcomes and/or impacts of health research partnerships? | To answer YES, authors must cite a synthesis study (e.g., scoping, systematic, narrative review) OR report at least two electronic sources were searched, including years and databases used (e.g., Central, EMBASE, and MEDLINE). |
| SR2 | Is the evaluation tool based on the experience/expertise of key partners? | To answer YES, the paper must explicitly state that key partners (e.g., researchers, patients, clinicians, policy makers etc.) were consulted during the process of development or modification, use, evaluation and/or validation of the tool. |
| SR3 | Is the tool based on a conceptual/theoretical framework involving health research partnership outcomes and/or impact assessment? | To answer YES, the framework must include or relate to the concept of health research partnership outcomes and/or impact assessment and describe how it is operationalized. |
| SR4 | Was the tool tested for validity (i.e., the tool evaluates what it is purported to evaluate)? | To answer YES, the tool must have evidence for validity (any source, including face and/or content validity). |
| SR5 | Was the tool tested for reliability (i.e., the tool produces stable and consistent results)? | To answer YES, the tool must have evidence for reliability (any source, including intra-rater reliability). |
| **Partner Perspective (PP)** | | |
| PP1 | Were partners involved as co-designers in the development or modification, use, evaluation and/or validation of the tool? | To answer YES, partners must have been co-designers during any stage of the development/modification, use, evaluation and/or validation of the tool. |
| PP2 | Is the tool designed to be self-administered by partners? | To answer YES, relevant partners must be explicitly identified as the target users of the tool. |
| PP3 | Does the tool explicitly state that partnership outcome and impact assessment results must be reported back to partners? | To answer YES, there must be an explicit statement that results will be communicated back to partners. |
| PP4 | Was the tool specifically designed to assess level of involvement among partners in health research partnerships? | To answer YES, there must be a clear statement that the tool assesses the level of partner involvement (on the IAP2 spectrum or other level of involvement scale). |
| PP5 | Does the tool capture the influence of partners? (e.g., the extent to which partners influenced the process, final decisions, etc.) | To answer YES, the tool must ask at least one question about partners’ perceptions of partnership member’s influence. |
| **Comprehensiveness (C)** | | |
| C1 | Does the tool document the context of the health research partnership? | To answer YES, the tool must ask at least one question about the internal and/or external partnership context (e.g., the nature of the issue). |
| C2 | Does the tool assess the partnership process? | To answer YES, the tool must have at least one question about the partnering process. |
| C3 | Does the tool document the outcome and/or impact of the health research partnership? | To answer YES, the tool must have at least one question about the perceived outcomes/impacts of health research partnership. |
| C4 | Does the tool monitor the partnering process at multiple moments? | To answer YES, the tool is explicitly designed to be used more than once during the project. |
| C5 | Does the tool consist of both open- and closed-ended questions? | To answer YES, the tool must consist of a combination of both types of questions (one of each at least). |
| **Usability (U)** | | |
| U1 | Is the purpose of the tool stated? | To answer YES, the purpose of the evaluation tool must be explicitly stated. |
| U2 | Is the evaluation tool freely accessible? | To answer YES, the evaluation tool should be freely accessible through an open access journal or publicly available on the Web. |
| U3 | Is the evaluation available in an applicable format? | To answer YES, the tool should be accessible in its complete form, and be ready to use. |
| U4 | Is the evaluation tool easy to read and understand? | To answer YES, the readability score must be reported in the paper (and be in readable score range), or the tool co-designed/piloted with relevant partners prior to use |
| U5 | Is the tool accompanied by instructions for use? | To answer YES, instructions must be provided about how to use the tool in the text or in the tool itself. |

**Reporting Scores:** A total criterion score for each of the four criteria will be calculated out of 5 points, for each study. These four criteria scores will be tallied and presented graphically as a percentage out of 100.

# **Appendix 6** Year of Publication for Included Studies

(n=166)


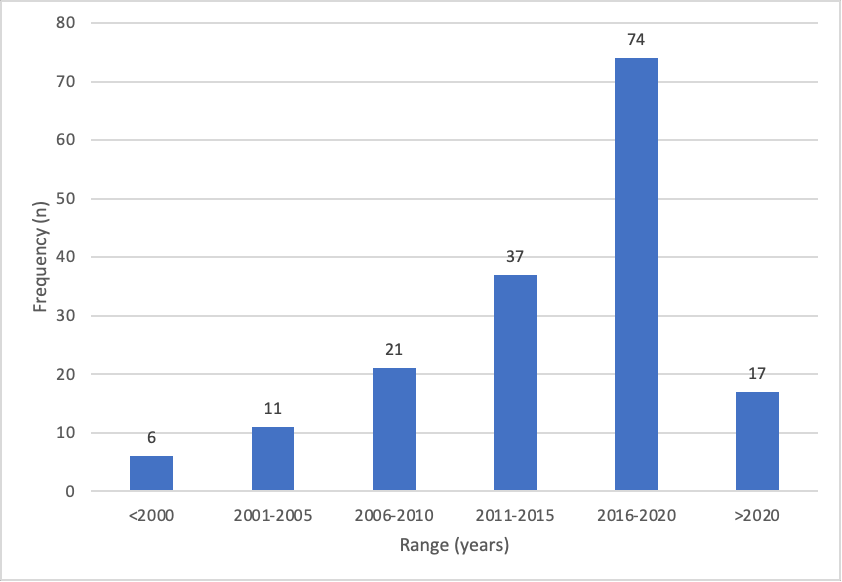


# **Appendix 7** Partnership Characteristics

(N=166 with 3 companion reports)

a companion report; b Initiating Partners (denotes those who commenced the partnership, including Researchers, Funders, Non-profit Organizations, Service User Researchers, Academic Institutions, Clinicians, Government, Foundations, Community Members, Partnerships)

| **First author, Year** | **Partnership purpose** | **Initiating partner(s)^b^** | **Funding Source (category)** |
| --- | --- | --- | --- |
| Butterfoss, 1996 | To plan and implement alcohol, tobacco, and other drug abuse prevention strategies in communities. | NR | US Government (Department of Health and Human Services) |
| Goodman, 1996 | To prevent alcohol, tobacco, and other drug abuse, and related risks. | NR | US Government (Department of Health and Human Services) |
| Goldstein, 1997 | To address focus areas of tobacco free lifestyles, appropriate dietary patterns, regular physical activity, and use of appropriate preventive health services. | State Health Department | State Health Department |
| Kegler, 1998 | To accelerate the decline in smoking in participating states. | Partnership (State Health Department, Foundation) | State Health Department, Foundation |
| Armbruster, 1999 | To develop new health, safety, and support services through a community partnership with older adults residing in a low-income urban neighborhood. | NR | NR |
| Chan, 2000 | To influence long-term health and human services practices in communities. | Partnership (Non-Profit Organizations, Foundations, Endowment, Industry) | Foundations, Endowment |
| Lantz, 2001 | To promote and support collaborative, community-based participatory research that improves family and community health by (1) working in partnership with communities to design, implement, and evaluate health-related interventions and programs to benefit and build capacity in the involved communities and (2) to increase the understanding and application of community-based participatory approaches to public health research. | Non-Profit Organization | US Government (Centers for Disease Control and Prevention (CDC)) |
| Shortell, 2002 | To increase the ability of public-private partnerships to address community health improvement issues that require cross-sector collaboration, including building partnership infrastructure and capacity, and funding specific activities or programs. | NR | Trust, Foundations, Non-Profit Organizations |
| Weiss, 2002 | NA (multi-partnership study) | Researcher | Foundation |
| Schulz, 2003 | To understand and reduce sources of occupational stress and strengthen psychosocial factors thought to mediate the relationship between these stressors and health in order to: (1) strengthen public health education and practice by linking academic and agency professionals with people from vulnerable neighborhoods, and (2) to promote the public’s health by enhancing the capacity of community members and community-based organizations and to conduct interdisciplinary, community-driven participatory research that improves family and community health, with a particular focus on addressing underlying social determinants of health. | NR | Foundation, Union, Industry, US Government (Centers for Disease Control and Prevention (CDC), National Institutes of Health (NIH)) |
| Cotter, 2003 | To increase training and individual support for home care aides in rural areas to serve persons with Alzheimer and related disorders. | NR | State Government |
| Butterfoss, 2004 | To provide training and technical assistance that enabled councils to function more effectively as community-based coalitions within a statewide network. To enhance and strengthen existing perinatal councils, to develop local coalitions in underserved areas to help coalitions focus on action and advocacy in addition to traditional consultation and training, to assist coalitions to plan interventions that are guided by data and evaluated. | NR | State Health Department |
| El Ansari, 2004 | To provide undergraduate medical, nursing, and allied health students the opportunity to learn and experience team-based, non-hospital primary health care in community settings, to provide an opportunity to initiate fundamental changes in curriculum design, community responsiveness, and educational philosophy in relation to research, service provision, policy, and leadership. | Foundation | Foundation |
| El Ansari, 2004 | To serve disadvantaged communities and address health, educational, and community development concerns. | Foundation | Foundation |
| Metzger, 2005 | To improve local health service delivery through (a) identification of community health needs, (b) delivery of a coordinated continuum of care services, (c) management within fixed financial resources, and (d) accountability to the community. | Partnership (Non-Profit Organizations, Foundations, Endowment, Industry) | Non-Profit Organizations, Foundations, Endowment, Industry |
| Kramer, 2005 | To assess whether coalition partners could mobilize resources to support community-wide, comprehensive, effective, and sustainable programs to prevent initial and repeat pregnancies. | US Government (Centers for Disease Control and Prevention (CDC) | US Government (Centers for Disease Control and Prevention (CDC)) |
| Kegler, 2005 | To demonstrate that community partners could mobilize and organize community resources in support of comprehensive, effective, and sustainable programs to prevent initial and repeat pregnancies. | US Government (Centers for Disease Control and Prevention (CDC) | US Government (Centers for Disease Control and Prevention (CDC)) |
| Cramer, 2006 | To encourage tobacco prevention among youth and reduce exposure to second-hand smoke in public places in metro areas. | NR | NR |
| Savitz, 2007 | To identify factors that facilitate or impede the adoption of quality improvements, to improve the quality of care for patients with adult-onset diabetes across the partnership, and to design, develop, implement, and evaluate a case study to help communities integrate resources to respond to health care–related homeland security needs; 2. To improve quality of care in 116 community- based primary care practices in 38 states using the same electronic medical record software and 3. to study implementation of evidence-based initiatives in quality improvement and bioterrorism preparedness. | Researcher | US Government (Agency for Health Care Research and Quality (AHRQ)) |
| Barber, 2007 | NA (multi-partnership study) | Researcher | UK Government (National Health Service (NHS)) |
| Feinberg, 2008 | To engage local leaders and citizens in a prevention coalition to reduce adolescent problem behaviors. | State Government | State Government |
| Feinberg, 2008b | To reduce adolescent problem behaviors through a set of well-planned stages, beginning with the formation of a coalition of community leaders or “prevention board.” | State Government | State Government |
| Wyatt, 2008 | To equip consumers, carers and their authorized representatives with the knowledge, skills, and confidence to participate in research according to their wishes, and ensure that health professionals involved in commissioning, managing, and conducting research understand and embrace consumer involvement so they can work in effective partnership with consumers. | Researcher | UK Government (NHSE), Spanish Government (Ministry of Health) |
| Lovell, 2008 | To understand and explore accessibility challenges facing others with a range of disabilities, the role of local government in facilitating access for persons with disabilities, and the implications new provincial legislation aimed at increasing accessibility might have at the local level. | Researcher | NR |
| Garner, 2008 | To join together in a common effort to build a healthy and safe community, reducing the risks of alcohol, tobacco, and other drugs while promoting the healthy attitudes and behaviors of youth in the region. | NR | Non-Profit Organization, State Government, Local Council |
| Orr Brawer, 2008 | NA (multi-partnership study) | NR | Foundation, Federal, State and Local Grants, Donors |
| Adily, 2009 | To provide funding for researchers and partner agencies to work together, define research questions, undertake research, and interpret the findings. | NR | Australian Government (National Health and Medical Research Council), Non-Profit Organization, Foundation |
| King, 2009 | NA (multi-partnership study) | NR | Canadian Government (Social Sciences and Humanities Research Council), Provincial Government (Ontario Ministry of Health), Non-Profit Organization. |
| Van Olphen, 2009 | To influence research projects designed to improve understanding of the role of environmental factors in pubertal development, as a window on breast cancer etiology. | Researcher | US Government (National Institutes of Health (NIH), National Institute of Environmental Health Sciences (NIEHS), National Cancer Institute (NCI), Department of Health and Human Services (DHHS)) |
| Sunderland, 2009 | To engage in: (1) joint planning around local chronic disease issues; (2) devising objectives and desired outcomes of the partnership; and (3) identifying and implementing at least one new chronic disease initiative to achieve these desired objectives and outcomes in the initial partnership period. | Non-Profit Organization | Australian Government |
| Tolma, 2009 | To identify health problems, prioritize them, and create a plan of action to address identified issues. | Community Leaders | Non-Profit Organization, Civic and County Government (Health Departments), Foundation |
| Barnidge, 2010 | To improve care and support for diabetes self-management more comprehensively and seamlessly than any partner could do alone. | Funder | Foundation |
| Blevins, 2010 | To support an empirical exploration of clinical interventions developed by frontline providers, with research and technical support provided by research mentors. | Clinicians | US Government (Department of Veterans Affairs) |
| Wagemakers, 2010 | NA (multi-partnership study) | Researcher | Dutch Government (Netherlands Institute for Health Promotion (NIGZ), Netherlands Organization for Health Research and Development (ZonMw)) |
| King, 2010 | NA (multi-partnership study) | Researcher | Canadian Government (Social Sciences and Humanities Research Council), Provincial Government (Ontario Ministry of Health), Non-Profit Organization, Foundation |
| Wright, 2010 | To engage in a collaborative effort and develop a tool to assess the quality and impact of user involvement in published research and funding applications. | NR | NR |
| Ziff, 2010 | To implement clinical, biological, and behavioral research with youth at risk for- or with HIV/AIDS through coalitions. The coalitions aim to decrease HIV rates among youth by catalyzing community structural change focused on creating new or modifying existing policies, programs, and practices in ways that are expected to curb the spread of the disease and ultimately have an overall impact on youth HIV rates. | US Government (National Institutes of Health) | US Government (National Institute of Health (NIH)) |
| Raine, 2010 | To develop sustainable collaborative partnerships that improve the health of the community; and to gather and use evidence to influence values, change environments and create a culture supportive of healthy living. Specific goals are to: (1) reduce prevalence of overweight and chronic disease risk, (2) increase community capacity to promote health, and (3) inform policy, practice, and research decisions. Ultimately, the community– academic–government partnership aims to create evidence for decisions regarding health promotion activities and resources which may eventually be incorporated into standard practice for communities. | Partnership (Government, Academics) | Canadian Government (Canadian Institutes of Health Research), Provincial Government (Alberta Health & Wellness), Non-Profit Organization |
| Jones, 2011 | NA (multi-partnership study) | NR | Republic of Ireland (Health Services) |
| Jones, 2011b | NA (multi-partnership study) | NR | NR |
| Payne, 2011 | To provide a forum for participation and consultation and convey community perspectives and guidance to enhance successful delivery of the project. | Researcher | National Government (National Health and Medical Research Council), State Government Health Promotion Foundation of Western Australia) |
| Perkins, 2011 | To achieve a set of intervention goals across a number of developmental phases and to implement evidence-based programs designed to support positive youth development and reduce early substance use and other problem behaviors. | Researcher | US Government (National Institutes of Health (NIH)) |
| Sanchez, 2011 | To develop a multistage, participatory evaluation design in conjunction with health council coordinators, including the development of a health council logic model, an “implementation” evaluation of the variability of council strategies and activities, and, ultimately, an outcome evaluation linking council actions to intermediate system change and longer-term outcomes. | Researcher | State Health Department |
| VanDevanter, 2011 | To evaluate the functioning of a community–academic partnership created to decrease hepatitis B health disparities among Asian Americans. | Researcher | US Government (Centers for Disease Control and Prevention (CDC)) |
| Bilodeau, 2011; Bilodeau, 2019^a^ | To reduce social inequalities (e.g., education, housing, food, urban environment) through intersectoral partnerships. | Researcher | Canadian Government (Canadian Institutes of Health Research), Non-Profit Organization (Fondation Canadienne de la recherche sur les services de santé (FCRSS)); Provincial Government (Québec – Fonds de recherche du Québec – Société et culture; Interactions Centre de recherche et de partage des savoirs) |
| Allen, 2011 | To identify effective means for training and developing community-engaged research, and to support and promote nascent CBPR research collaborations between university researchers and community members. | Researcher | Academic Institution |
| Curro, 2012 | To decrease the time required from initial scientific discovery to clinical application thereby improving the nation’s healthcare and eventually reducing costs. | US Government (National Institutes of Health) | US Government (National Institutes of Health (NIH)) |
| El Ansari, 2012 | To achieve interprofessional, community-sensitive health professions education in South African multi-stakeholder partnerships. | Foundation | Foundation |
| CIHR, 2013; McLean, 2012^a^ | NA (multi-partnership study) | NR | Canadian Government (Canadian Institutes of Health Research) |
| Vale, 2012 | NA (multi-partnership study) | Researcher | UK Government (Medical Research Council) |
| Martinez, 2012 | To examine the development processes of partnerships focused on promoting worker health and safety among the Latino population. | Researcher | US Government (National Institutes of Health (NIH)) |
| Woodland, 2012 | NR | NR | NR |
| Tataw, 2012 | To 1) deliver a coordinated continuum of health services to children and families; 2) To reduce low levels of health services utilization and improve preventive health techniques and disease self-management for families with the ultimate goal of attaching each child to a medical home, and 3) To create a road map for the positive development of youths and families. | NR | NR |
| Kagan, 2012 | The purpose of community-researcher collaboration was to 1) improve intra- and inter-network community input; 2) support training for local Community Advisory Boards; 3) increase representation of vulnerable populations and communities in resource-limited areas; 4) address challenges to trial participation, and 5) harmonize best practices for community participation. | US Government (National Institutes of Health) | US Government (National Institutes of Health (NIH)) |
| Brown, 2012 | To guide communities to adopt a science-based approach to community prevention planning and services. | State Government | State Government |
| Braun, 2012 | To reduce the unequal burden of cancer in minority and disadvantaged communities across the United States and American Samoa. | NR | US Government (National Institutes of Health (NIH)) |
| Stedman-Smith, 2012 | To collaborate with faculty researchers and work with communities on issues of pesticide use and exposure, especially as it affects pregnant and preconception women and small children who are at the greatest risk, but whose opinions were not captured in surveys. | Partnership (Academic Institution, community members) | Non-Profit Organization, Academic Institutions, US Government (National Institute for Occupational Safety and Health (NIOSH)), Endowment |
| Nargiso, 2013 | To offer a structured, sequential, data-driven approach that explicitly targets environmental conditions in the community and aims for change in substance use and problems at the population level. | Researcher | US Government (Department of Health and Human Services) |
| Watson-Thompson, 2013 | To use a 5-phase model for promoting change and improvement to enhance and support capacity building in community coalitions. The goal was to address and improve targeted public health outcomes at a population level through the Community Anti-Drug Coalitions of America. | Researcher | US Government (National Institute on Drug Abuse (NIDA)) |
| Khodyakov, 2013 | NA (multi-partnership study) | NR | US Government (National Institute of Mental Health (NIMH)), Academic Institution |
| Patterson, 2014 | NA (multi-partnership study) | Service User Researcher | UK Government (National Health Service (NHS)) |
| Rosella, 2018; Rosella, 2014^a^ | To partner with regional and provincial decision makers in order to facilitate use of DPoRT and evaluate i) the effectiveness of partnerships (research team + DPoRT users) ii) explore strategies that facilitate uptake and overcome barriers to DPoRT use; and iii) implement and evaluate the knowledge translation approach. | Researcher | Canadian Government (Canadian Institutes of Health Research, Public Health Agency of Canada) |
| Perkins, 2014 | To advance nursing practice and improve the quality of care. | NR | NR |
| Chang, 2014 | To enable teachers to understand the concept of Health Promoting Schools and share program implementation experiences of healthy weight, healthy diet, oral health, vision health, injury prevention, mental health, sex education, smoking prevention, and drug abuse prevention aspects of the program. | Taiwan Government | Taiwan Government (Ministry of Education) |
| Arroyo-Johnson, 2015 | As part of an effort to reduce disparities in cancer health, the goal of the two partnerships was to 1) improve self-reported colorectal cancer screening adherence rates and 2) prevent tobacco and other substance use intentions among Latino youth through parent-youth relationship building. | NR | US Government (National Institutes of Health (NIH)), Academic Institution, Non-Profit Organizations |
| Brown, 2015 | To guide communities to adopt a science-based approach to community prevention planning and services. | State Government | State Government |
| Gamble, 2015 | NA (multi-partnership study) | Researcher | UK Government (National Institute for Health Research), Non-Profit Organization |
| Murphy, 2015 | To promote better and more equitable health worldwide through the production and use of knowledge. | Researcher | Government of Canada (International Development Research Centre (IDRC)) |
| Soper, 2015 | To forge a mutually beneficial, forward-looking partnership between a university and the surrounding NHS organizations, focused on improving patient outcomes through the conduct and application of applied health research by 1) conducting high-quality applied health research; 2) supporting the ‘translation’ of research evidence into practice; and 3) increasing the capacity of NHS organizations to engage with and apply research. | UK Government (National Institute for Health Research (NIHR)) | UK Government (National Institute for Health Research (NIHR)) |
| Truiett-Theodorson, 2015 | To contribute to the reduction of infant mortality by providing opportunities for overweight and obese, low-income, postpartum women to lose weight and improve their eating habits for subsequent pregnancies, and those of their families. | Researcher | US Government (Department of Health and Human Services) |
| Wilson, 2015 | NA (multi-partnership study) | Researcher | UK Government (National Institute for Health Research (NIHR)) |
| Bornstein, 2015 | To increase population levels of physical activity in the United States. | NR | NR |
| Oetzel, 2015 | NA (multi-partnership study) | Researcher | US Government (Native American Research Centers for Health, National Institute of Drug Abuse, National Center for Research Resources, Office of Behavioral Social Sciences Research, National Cancer Institute, and Health Resources Services Administration) |
| Oetzel, 2015b | NA (multi-partnership study) | NR | US Government (Native American Research Centers for Health, National Institute of Drug Abuse, National Center for Research Resources, Office of Behavioral Social Sciences Research, National Cancer Institute, and Health Resources Services Administration) |
| Arora, 2015 | To create a culturally appropriate measure of the essential components of the relationships among the research team partners, which could be used at different points in the partnership process. | Partnership (Researchers and Community Leader) | US Government (Centers for Disease Control and Prevention (CDC), National Institutes of Health (NIH)) |
| Stocks, 2015 | To create a Research User Group (RUG) that fulfilled both a strategic governance role and contributed PPI to research activities addressing patient safety in primary care. | Researcher | UK Government (National Institutes of Health Research (NIHR)) |
| Brown, 2016 | To address drug and violence prevention in Mexican and US communities. | NR | US Government (Department of Anti-Narcotic Affairs), State Government |
| Gibbons, 2016 | To test the hypothesis that CBPR-based engagement processes can improve community–academic relationships among historically hyper-researched, academically resistant communities using a CBPR approach and purposeful collaboration from inception. | Academic Institution | NR |
| Larkan, 2016 | To conduct exploratory research with research partners and staff of the Center for Global Health to build an evidence-informed framework for successful global health research partnerships. | NR | NR |
| Merkel, 2015 | NA (multi-partner study) | Researcher | US Government (National Institutes of Health (NIH)) |
| Puyalto, 2016 | To seek the opinions and views of members of an Advisory Committee composed entirely of people with intellectual disabilities and those of researchers without disabilities, on the experience of participating in research on transition to adulthood. The Advisory Committee advised and worked together with the research team in different phases of the study. | Researcher | Spanish Government (Ministry of Economy and Competitiveness (MINECO)) |
| Robbins, 2015 | To collaborate with stakeholder co-investigators, recruited to provide important and ongoing contributions to the research design and implementation of the study. | Researcher | Non-Profit Organization |
| Finch, 2016 | To develop practical guidelines to help community sporting organizations adopt a sustainable approach to safety and to implement specific injury prevention interventions. Specifically, the larger project aimed to develop and evaluate the strategic implementation of an evidence-based sports safety programme and gather together a range of stakeholder groups to improve community sport safety. | Partnership (Researchers and Partners) | Australian Government (National Health and Medical Research Council (NHMRC)) |
| Goold, 2016 | To engage minority and underserved communities in deliberations about health research priorities to increase community voice in how limited health research resources are allocated. | Partnership (Community Member and Academic) | NR |
| Abelson, 2016 | To develop a common evaluation tool for use in a variety of health system organizations that would collectively contribute to improvements in the quality of public and patient engagement practice across the country. | Researcher | Canadian Government (Canadian Institutes of Health Research) |
| Dugan, 2016 | To deliver a participatory intervention research program aimed at improving corrections officer well-being. | Partnership [US Government (National Institute for Occupational Safety and Health (NIOSH) Center of Excellence) and Academic Institution] | US Government (National Institute for Occupational Safety and Health (NIOSH)) |
| Brutt, 2017 | To understand how patients could contribute to and prioritize clinically relevant outcomes of meta-cognitive interventions and provide their perceptions of research involvement after a workshop. | Researcher | German Government (Ministry of Education and Research) |
| Goodman, 2017 | To create a national model for eliminating disparities in cancer through community-based partnerships, to be a catalyst for change in the region by fostering healthy communities and break down barriers to quality cancer care. | Partnership (Researchers, Research Staff, Community Member) | US Government (National Institutes of Health (NIH)) |
| Littlecott, 2017 | To involve multisectoral stakeholders and (i) hold collaborative meetings; (ii) review evidence on effectiveness of physical activity promotion initiatives; (iii) to re-analyze and collate existing data; (iv) to compare approaches to physical activity promotion against evidence-based criteria; (v) to hold effective focus groups; (vi) to produce recommendations for physical activity promotion for older people; and (vii) prepare a research proposal. | Researcher | UK Government (Medical Research Council) |
| Scarinci, 2017 | To involve multiple disciplines and collaborations between researchers, government agencies, and community organizations to conduct research where findings translate into 'sustainable individual, community and systems level changes to improve population health'. The focus is to establish centers in health disparities science for three priority areas–social determinants of health, men’s health research, and health policy research. | Funder | US Government (National Institute on Minority Health and Health Disparities (NIMHHD)) |
| Okazaki, 2017 | To explore how university-based, coordinated strategic partnerships contributed to the process of culturally adapting and implementing evidence-based health programs. | Researcher | Academic Institution |
| Ray, 2017 | To examine the experience of families referred for subspecialty care and examine whether stakeholder engagement achieved specific process and outcome goals. | Researcher | US Government (Agency for Health Care Research and Quality (AHRQ), National Institutes of Health (NIH)), Healthcare Organization |
| Weeks, 2017 | NA (multi-partnership study) | NR | NR |
| Jose, 2017 | To improve researchers’ understanding of the needs of policymakers and improve policy-makers’ understanding of research methods and the interpretation of research findings, provide unique higher degree opportunities for training in public health research and research translation, to develop new collaborative projects and to improve the health and productivity of the State Service workforce. | NR | Academic Institution, State Health Department |
| Carroll, 2017 | NA (multi-partner study) | Researcher | Non-Profit Organization |
| Blackburn, 2018 | NA (multi-partnership study) | NR | UK Government (National Institute for Health Research (NIHR), Non-Profit Organizations |
| Burrows, 2018 | To talk to researchers about their work, discuss topics including approaching potential participants, designing future studies, and features of the technology being developed, to develop collaborative relationships between the research team and members of the public, and to establish partners in research and design activities. | Researcher | UK Government (UK Engineering and Physical Sciences Research Council (EPSRC)) |
| Forsythe, 2018 | NA (multi-partnership study) | Funder (Non-Profit Organization) | Non-Profit Organization |
| Jones, 2018 | Partnerships were focused on increasing involvement in physical activity, improving the health of children and families, and alleviating the effects of poverty. | NR | Republic of Ireland (Health Services) |
| Kazmerski, 2018 | To pursue a partnership to create a series of dedicated online health guides for adolescent and young adult women with cystic fibrosis pertaining to sexual and reproductive health. | Partnership (Researchers, stakeholders, adolescent, and young adult women with cystic fibrosis) | Non-Profit Organization |
| Korn, 2018 | NA (multi-partnership study) | NR | US Government (National Institutes of Health (NIH)), Non-Profit Organization |
| Mann, 2018 | To ensure maximum relevance of the trial intervention for people with multimorbidity, to enhance response to recruitment and follow-up, to partake in qualitative data analysis and help interpret results and to gain assistance with dissemination to interested groups and individuals. To establish PPI governance within the trial and to facilitate new primary care research idea generation. | Researcher | UK Government (National Institute for Health Research (NIHR)) |
| McIsaac, 2018 | To support the development of research questions and data collection methods, analysis of data, interpretation, and the contextualization of findings for policy and practice, and dissemination of results. | Partnership (Knowledge Users and Researchers) | Canadian Government (Canadian Institutes of Health Research), Non-Profit Organizations, Foundation |
| West, 2018 | To learn about perspectives on what trustworthiness means to individuals within the context of their experience in community-academic research partnerships, to learn about what enhances or hinders trust and trustworthiness, and how they have dealt with institutional barriers to researcher trustworthiness and to inform a tool for partnerships to self-evaluate community perceptions of the research partnership | NR | NR |
| Lobo, 2018 | To support and facilitate a collaboration between individuals and organizations from three stakeholder groups in the sexual health and blood-borne virus sector. | Partnership (policymakers (government organizations); service providers (health professionals and practitioners in government and non-government organizations [NGOs] who deliver health services and health promotion programs to the general public); and researchers (universities and national SHBBV research centres) | State Health Department |
| Mathie, 2018 | To initiate and steer the direction of the research, keep the study relevant, collect and interpret data and act as links to other PPI organizations. | NR | UK Government (National Institute for Health Research (NIHR)) |
| Oetzel, 2018 | NA (multi-partnership study) | NR | US Government (Native American Research Centers for Health, National Institute of Drug Abuse, National Center for Research Resources, Office of Behavioral Social Sciences Research, National Cancer Institute, and Health Resources Services Administration) |
| Nowell, 2018 | To support an arthritis patient partnership with comparative effectiveness researchers in the patient-centered outcomes research network. | Researcher | Non-Profit Organizations, Industry |
| Kendall, 2018 | NA (multi-partnership study) | Researcher | Canadian Government (Canadian Institutes of Health Research) |
| Walton, 2018 | To provide the study team insights from participants regarding best approaches for recruitment, perceptions of and suggestions for the assessment and intervention protocols and suggestions for knowledge dissemination of study findings (end-of-grant KT), as well as to increase participant/family engagement. | Researcher | Canadian Government (Canadian Institutes of Health Research), Academic Institution, Provincial Government (Ontario Ministry of Agriculture, Food and Rural Affairs (OMAFRA)) |
| Hamilton, 2018 | To develop and examine the content and face validity of a novel outcome measure for assessing the quality of patient engagement in research projects from a patient partner perspective. | Researcher | Canadian Government (Canadian Institutes of Health Research), Non-Profit Organization |
| Haynes, 2019 | To co-design, implement and evaluate a CBPAR project on rheumatic heart disease prevention in a remote Aboriginal community and to describe the processes, outcomes, and learnings that emerged through close adherence to the empowering principles embedded in CBPAR. | Community Leader | Professional Regulatory Body (Australia), Australian Government (National Health and Medical Research Council (NHMRC)), Non-Profit Organization, State Governments |
| Goodman, 2019 | NA (multi-partner study) | Researcher | Non-Profit Organization |
| Warner, 2019 | To pilot and methodologically appraise the Active Involvement of Users in Research Observation Schedule and Questionnaire as advisors in a case study about refugee involvement in the development of mental health intervention research. | Researcher | Non-Profit Organization |
| Ken-Opurum, 2019 | To initiate, Additional, and support public health activities involving current health priorities including obesity prevention, wellness, mental health, systems of care, child well-being/maternal health, tobacco control, and substance abuse reduction. | NR | US Government (Department of Agriculture) |
| Tabriz, 2019 | To design, develop and implement a practice-integrated decision support tool for patients with a physician recommendation for colorectal cancer screening using a healthcare organization-research partnership. | Researcher | US Government (National Institutes of Health (NIH)) |
| Duran, 2019 | NA (multi-partnership study) | NR | US Government (National Institutes of Health (NIH), Centers for Disease Control and Prevention (CDC)) |
| Lessard, 2019 | To ensure direct, equitable and continuous engagement of patients in decision making dissemination and in qualitative and quantitative data collection, throughout the study in order to optimize research outcomes of patient engagement. | Researcher | Canadian Government (Canadian Institutes of Health Research), Industry, Non-Profit Organizations |
| Pavarini, 2019 | To support every stage of research, from refining research questions, to designing materials and research tools (e.g., interview guides, digital resources), recruiting, analyzing results, and disseminating. | Researcher | UK Government (National Institutes for Health Research (NIHR)), Charitable Foundation |
| Rarere, 2019 | To promote transformational improvements in health service delivery for Indigenous communities with the aim of identifying what makes health interventions work for communities. | Researcher | New Zealand Government (Ministry of Business, Innovation and Employment) |
| Barger, 2019 | To inform each phase of the trial from planning and design to implementation and dissemination. | Researcher | US Government (National Institutes of Health (NIH)), Non-Profit Organization |
| Abelson, 2019 | NA (multi-partnership study) | NR | Canadian Government (Canadian Institutes for Health Research, SPOR), Provincial Government (Ontario Ministry of Health and Long-Term Care, SPOR) |
| Roberge-Dao, 2019 | To collaboratively bridge the research-practice gap and improve patient outcomes and quality of care. | NR | Registered Charity |
| Crocker, 2019 | NA (multi-partner study) | NR | UK Government (National Institutes for Health Research (NIHR), Medical Research Council) |
| Hemphill, 2019 | To engage patients, family caregivers and other health‐care stakeholders, including clinicians, payers and policymakers, as active partners in prioritizing, designing, conducting, and disseminating research as a key strategy to produce useful evidence for health‐care decision making. | NR | Non-Profit Organization |
| Langlois, 2019 | To engage policymakers and implementers as leaders of the research and their involvement in all phases of the empirical endeavor by placing policymakers and programme managers in the position of co-principal investigators, with the objective of stimulating demand-driven empirical work. Ultimately, the collaboration is designed to increase the policy-relevance of research questions being addressed and enhance policymakers' and implementers' ownership of the research. | NR | Non-Profit Organization, Norwegian Government (Norwegian Government Agency for Development Cooperation (NORAD)), Swedish Government (Swedish International Development Cooperation Agency (SIDA)), UK Government (Department for International Development (DfID)) |
| Faulkner, 2019 | To enable service users to find the voice and the freedom to talk about profoundly sensitive issues and enable us to reach practitioners and policymakers with a view to effecting change. | Service User Researcher | UK Government (National Institute for Health Research (NIHR), Department of Health) |
| Soobiah, 2019 | To engage knowledge users in the systematic review process to compare effectiveness of geriatrician models of care. | Researcher | Canadian Government (Canadian Institutes of Health Research) |
| Blank, 2019 | To jointly develop, implement, and evaluate a creative and sustainable community-academic partnership infrastructure to build capacity for community-based research. | Researcher | US Government (National Institutes of Health (NIH)) |
| Dickson, 2020 | NA (multi-partnership study) | Community partner initiated: n=6, 3%  Researcher initiated: n=84, 47% Both initiated: n=87, 49% Other n=2, 1% | US Government (National Institutes of Health (NIH)), Non-Profit Organization |
| Rodriguez Espinosa, 2020 | NA (multi-partnership study) | NR | Foundation, Academic Institution, US Government (National Institutes of Health (NIH)) |
| Coombe, 2020 | To draw upon cumulative knowledge and experience and serve as key informants, determining the validity and clarity of- and finalizing the Delphi questionnaire. | Non-Profit Organization | US Government (National Institutes of Health (NIH)), Non-Profit Organization |
| Snijder, 2010 | To reduce injuries and improve safety in Aboriginal communities; specifically, to (a) select the most appropriate activities; (b) oversee the implementation of the activities with a focus on optimizing their fidelity and sustainability; (c) problem-solve challenges as they arise; (d) ensure that Aboriginal ethical values were respected and incorporated throughout the CBPR project; and (e) to contribute to research papers and reports. | Researcher | Australian Government (Department of Health), State Government (Ministry of Health) |
| O’Donovan, 2020 | To identify key water, sanitation, and hygiene (WASH) issues in rural communities by engaging with a diverse range of participants through collaboration and community outreach. | Researcher | UK Government (National Institute for Health Research (NIHR), Economic and Social Research Council) |
| Lucero, 2020 | NA (multi-partnership study) | NR | US Government (National Institutes of Health (NIH)) |
| Spitzer-Shohat, 2020 | To help ascertain the health needs of the community, find research evidence to inform the proposed intervention, develop an evaluation framework and accompany the project during its pilot. | Researcher | Registered Charity |
| Knudsen, 2020 | NA (multi-partnership study) | Researcher | US Government (National Institutes of Health (NIH)), Academic Institutions |
| Aguirre, 2020 | To reduce health disparities related to chronic disease through community engagement. | US Government (Centers for Disease Control and Prevention (CDC) | US Government (National Institutes of Health (NIH), Academic Institution |
| Hughes, 2020 | To co-design a survey tool that investigates the willingness of inpatients to engage with anti-microbial stewardship in hospital. | NR | Healthcare Organization |
| Hinrichsen, 2020 | The overall aim of the partnership is to promote public mental health in Denmark by bridging the gap between international recommendations, research, policy, and practice. | Partnership (Local Government, Non-Governmental Organizations) | Non-Profit Organization |
| Toledo-Chávarri, 2020 | To include patient perspectives, preferences, and values, to elicit important health outcomes measures and to understand barriers, facilitators, or suggestions for implementation. | Researcher | Spanish Government (Spanish Network of Agencies for Assessing National Health System Technologies and Performance (RedETS)) |
| Luchtenberg, 2020 | To participate as pediatric co-researchers by helping adult researchers identify qualitative themes from original data, exploring these themes in detail and providing recommendations on how to improve children's involvement in research. | Researcher | Academic Institution |
| Gafos, 2020 | To represent the wider study population, provide perspectives of individuals who may potentially benefit from the research results, and provide unique, first-hand experience of using (or wanting to use) pre-exposure prophylaxis in the prevention of HIV for men who have sex with men and trans women. | Researcher | UK Government (Medical Research Council) |
| Drebit, 2020 | To utilize patient experiential knowledge to inform planning and implementation that will ultimately lead to benefits that matter to patients: improved health, quality of care, patient-relevant outcomes, patient experience, and improved cost effectiveness for the health care system. | Researcher | Non-Profit Organizations, Provincial Government (British Columbia Ministry of Health), Academic Institution |
| Bhati, 2020 | To support the authentic engagement of patients in primary care research and specifically to examine patients’ experience of research study engagement. | Researcher | Provincial Government (Ontario Ministry of Health and Long-Term Care), Academic Institutions |
| van Schelven, 2020 | NA (multi-partnership study) | Researcher | Registered Charity |
| Haesebaert, 2020 | To create an organizational level partnership according to the patient engagement frameworks underpinning the model and to co-design new services and improve service quality. | Researcher | Canadian Government (Canadian Institutes of Health Research), Provincial Government (Quebec Ministry of Health and Social Services), Professional Governing Body, Academic Institution |
| Vat, 2020 | NA (multi-partner study) | Researcher | Canadian Government (Canadian Institutes of Health Research), Provincial Government (Newfoundland & Labrador), Industry |
| Birch, 2020 | To ensure the quality and relevance of the research from inception, and to facilitate effective translation and dissemination of the research findings. | Researcher | European Commission, UK Government (National Institutes of Health Research (NIHR), Academic Institutions, Healthcare Organization |
| Seeralan, 2021 | To investigate the needs and preferences of patients with lived experiences of depression using a feedback intervention after depression screening. | Researcher | German Government (Federal Joint Committee - Gemeinsamer Bundesausschuss) |
| Alexander, 2021 | To provide insight on program improvements and the development of a maintenance phase for future families through the adaptation, implementation, and evaluation of a regional family-based childhood obesity treatment program. | NR | US Government (National Institute on Minority Health and Health Disparities (NIMHHD)) |
| den Houting, 2021 | NA (multi-partner study) | Funder (Non-Profit Organization) | Australian Government (Department of Industry, Science, Energy and Resources) |
| Scholz, 2021 | To undertake collaborative research with mental health consumer researchers. | Researcher | Academic Institution |
| van Schelven, 2021 | NA (multi-partnership study) | Funder | Registered Charity |
| Hamilton, 2021 | To engage in all phases of the current study, by contributing to the study protocol, recruiting participants by sharing information actively and passively through networks, and discussing the objectives as well as reviewing the expected and proposed study findings through in-person and virtual/teleconference meetings, including assistance with manuscript drafting, review and feedback. | Researcher | Canadian Government (Canadian Institutes of Health Research), Non-Profit Organization |
| Boursaw, 2021 | NA (multi-partnership study) | Researcher | US Government (National Institutes of Health (NIH)) |
| Loban, 2021 | NA (multi-partnership study) | Researcher | Canadian Government (Canadian Institutes of Health Research), Provincial Government (Quebec Fonds de recherche du Quebec - Sante), Academic Institution, Australian Government (Department of Health) |
| Martinez, 2021 | To develop and implement best practices for evaluating stakeholder engagement through the Stakeholder Centric Instrumentation Process (SCIP) and facilitate valid, reliable, scalable, tailored, and equitable evaluation. | Researcher | Non-Profit Organization |
| Moore de Peralta, 2021 | To build a stronger state by supporting Latinx communities through education, advocacy, and leadership development. The partnerships assist community members in navigating complex health and social service systems, provide feedback to partner organizations, provide culturally tailored education to promote health and well-being, partner with mainstream organizations to help provide more effective health and social services to the Latinx population; and conduct leadership development for grassroots Latinx leaders. | Researcher | Academic Institution |
| Livingstone, 2021 | NR | Funder | NR |
| March, 2021 | To garner input and encourage co-learning on aspects of methodology, recruitment strategy, interpretation of findings, and planning for future research from school nurses on the subject of diabetes devices in school environments. | Researcher | Healthcare Organization |
| Enard, 2021 | To address health disparities and improve breast and prostate cancer outcomes among underserved African American communities. | Partnership (Academics and Community Partners) | Industry, Registered Charity, Healthcare Organization |
| Rasburn, 2021 | To design a strategy to engage with patient stakeholders to review and improve PPI in health technology assessments through a Patient Working Group. | Funder | NR |
| Knowles, 2021 | To work with public contributors to design ways for patients, carers, and members of the public to be involved in a Learning Health System (LHS). | Researcher | UK Government (National Institute for Health Research (NIHR)) |
| Nunn, 2021 | To involve participants in the co-design of a proposed multi-generational research study, and to understand how this involvement impacted study design. | Researcher | Academic Institution |
| Chung, 2021 | To test an online health care platform that generates personal recommendations and a shareable plan for primary care providers, targeted to caregivers of individuals with Down syndrome. The overall goal is to improve adherence to the national Down syndrome health care guidelines. | Researcher | Non-Profit Organization |

# **Appendix 8** Pragmatic Health Research Partnership Criteria Assessments

(n=166; n=205 tool scores)

Notes: 1 companion report; a not available; b Domain scores from the Health Research Partnerships Tool Evaluation

Criteria (adapted from Boivin et al, 2018 – modified criteria in Appendix); SR Scientific Rigor PP Partner Perspective

C Comprehensiveness U Usability

| **First author,**  **Year** | **Tool Name** | **Reported Tool**  **Type** | **Domain Scores^b^** | | | | **Tool Score**  **(%)** |
| --- | --- | --- | --- | --- | --- | --- | --- |
|  |  |  | **SR** | **PP** | **C** | **U** |  |
| Butterfoss, 1996 | - The Committee Member Survey (CMS) - The Plan Quality Index (PQI) | Toolkit | 3 | 3 | 3 | 3 | 60% |
| Goodman, 1996 | Phase 1 Formation:   - Forecast^a^ - Meeting effectiveness inventory (MEI) - Project Insight Form^a^ - Committee Survey Needs Assessment Checklist^a^ - Plan Quality Index (PQI)   Phase 2: Plan implementation (bespoke tools): Monitoring of actions, Worksheets for planning goals, processes, outcomes, impacts, Policy analysis  Phase 3: Impact:   - Key Leader Survey^a^ - Community Survey^a^ - Trend Data^a^ (bespoke) - Level of institutionalization Scale (LOIN) | Toolkit | 2 | 3 | 4 | 3 | 60% |
| Goldstein, 1997 | - Coalition Self-Assessment Tool | Survey | 1 | 3 | 3 | 4 | 55% |
| Kegler, 1998 | - Questionnaire | Questionnaire | 3 | 3 | 4 | 3 | 65% |
| Armbruster, 1999 | - ElderCARE Community Ownership Scale (modified from the Community Ownership Scale, Flynn 1995) | Questionnaire | 3 | 2 | 4 | 3 | 60% |
| Chan, 2000 | - Social Capital Index  (scale adapted from the Partnership Self-Assessment Survey, 1997) | Index | 2 | 1 | 3 | 1 | 35% |
| Lantz, 2001 | - 1997 - Detroit Community-Academic Urban Research Center (URC), Board Evaluation (Lantz et al, 2001, Schulz et al, 2003, Israel et al, 2012) | Survey | 0 | 3 | 4 | 1 | 40% |
|  | - 1998^a^ - Detroit Community-Academic URC Board Evaluation | Survey^a^ | . | . | . | . | . |
|  | - 1999 - Detroit Community-Academic URC | Survey | 0 | 3 | 4 | 1 | 40% |
| Shortell, 2002 | - Capability Index (from Partnership Self-Assessment Survey (PSAS), Health Research & Educational Trust, 1997) | Index | 3 | 2 | 3 | 1 | 45% |
| Weiss, 2002 | - Partnership Self-Assessment Tool (PSAT) | Questionnaire | 5 | 4 | 4 | 3 | 80% |
| Schulz, 2003 | - Questionnaire | Questionnaire | 2 | 4 | 5 | 1 | 60% |
| Cotter, 2003 | - Log | Log | 0 | 1 | 5 | 2 | 40% |
| Butterfoss, 2004 | - Coalition Needs Assessment (CNA) - Coalition Effectiveness Inventory (CEI) - Meeting Effectiveness Inventory (MEI) - CTAT survey | Toolkit | 2 | 4 | 5 | 5 | 80% |
| El Ansari, 2004 | - Survey | Survey | 3 | 4 | 3 | 3 | 65% |
| El Ansari, 2004 | - Partnership Member Survey | Survey | 2 | 3 | 3 | 2 | 50% |
| Metzger, 2005 | - Partnership self-assessment survey (PSAS)-derived scales | Scales | 3 | 2 | 3 | 1 | 45% |
| Kramer, 2005 | - Coalition Member Survey | Survey | 2 | 3 | 5 | 3 | 65% |
|  | - Post Site Visit Survey | Survey | 2 | 3 | 5 | 3 | 65% |
| Kegler, 2005 | - Coalition Member Survey | Survey | 3 | 4 | 4 | 2 | 65% |
| Cramer, 2006 | - Internal Coalition Effectiveness (ICE) Instrument | Survey | 4 | 2 | 3 | 2 | 55% |
| Savitz, 2007 | - Partnership Strength Survey | Survey | 2 | 4 | 5 | 4 | 75% |
| Barber, 2007 | - Postal Survey | Survey | 2 | 3 | 4 | 1 | 50% |
| Feinberg, 2008 | - CTC Coalition Web-Based Self-Report Questionnaire | Questionnaire | 3 | 2 | 5 | 1 | 55% |
| Feinberg, 2008b | - CTC Coalition Web-Based Self-Report Questionnaire | Questionnaire | 3 | 4 | 5 | 1 | 65% |
| Wyatt, 2008 | - Evaluation of User and Carer Involvement in Primary Care Research Projects Questionnaire | Questionnaire | 1 | 3 | 4 | 4 | 60% |
| Lovell, 2008 | - Participatory Research Evaluation Questionnaire (modified from the Institute of Health Research, 1995) | Questionnaire | 0 | 2 | 4 | 3 | 45% |
| Garner, 2008 | - Community Key Leader Survey (Goodman & Wandersman, 1996) | Survey | 0 | 1 | 4 | 3 | 40% |
|  | - Coalition Group Member Survey (University of Wisconsin- Cooperative Extension, 1998) | Survey | 0 | 2 | 4 | 3 | 45% |
| Orr Brawer, 2008 | - Partnership Self-Assessment Tool (PSAT) (Lasker & Weiss, 2002) | Questionnaire | 2 | 4 | 4 | 5 | 75% |
|  | - Social Capital Survey  (Provan et al, 2013; Israel et al, 1994; Bullen & Onyx, 1995) | Survey | 3 | 2 | 3 | 4 | 60% |
| Adily, 2009 | - Survey | Survey | 1 | 3 | 4 | 3 | 55% |
| King, 2009 | - Community Impacts of Research Oriented Partnerships (CIROP) | Questionnaire | 4 | 2 | 4 | 5 | 75% |
| Van Olphen, 2009 | - Quantitative Rating Form: Adapted Community-Based Participatory Research Guidelines | Questionnaire | 3 | 4 | 3 | 3 | 65% |
| Sunderland, 2009 | - Partnership Self-Assessment Tool (modified from the Victorian Partnership Assessment Tool - VicHealth, 2003) | Questionnaire | 1 | 2 | 4 | 1 | 40% |
| Tolma, 2009 | - Profile of Collaboration Survey (Chrislip & Larson, 1994) - Interactive group evaluation form - Meeting observation form - Facilitator check-off list - Random Electronic Survey | Toolkit | 2 | 3 | 4 | 3 | 60% |
| Barnidge, 2010 | - Partnership Attributes Checklist - Organizational Capacity Checklist - Intermediate-level Outcome Checklist - Taking Action - Making Improvements Tool | Toolkit | 2 | 4 | 4 | 5 | 75% |
| Blevins, 2010 | - Collaboration Scale  (based on Naylor et al, 2002) | Survey | 1 | 4 | 3 | 3 | 55% |
| Wagemakers, 2010 | - Coordinated Action Checklist | Checklist | 5 | 2 | 4 | 4 | 75% |
| King, 2010 | - Community Impacts of Research Oriented Partnerships (CIROP) Questionnaire - Background Information Form for Research Partnerships - Research Contact Checklist - CIROP Respondent Form | Toolkit | 4 | 2 | 5 | 5 | 80% |
| Wright, 2010 | - Critical appraisal criteria for assessing the quality and impact of user involvement on health research | Criteria | 2 | 4 | 3 | 4 | 65% |
| Ziff, 2010 | - Wilder Collaboration Factors Inventory (Mattessich, Murray-Close & Monsey, 2001) | Questionnaire | 3 | 1 | 4 | 3 | 55% |
| Raine, 2010 | - Community Capacity Building Tool (CCBT) (Maclellan-Wright et al, 2007) | Questionnaire | 5 | 4 | 4 | 5 | 90% |
| Jones, 2011 | - Jones Synergy Scale | Scale | 4 | 2 | 2 | 2 | 50% |
| Jones, 2011b | - Jones Trust Scale | Scale | 3 | 2 | 2 | 2 | 45% |
| Payne, 2011 | - Questionnaire | Questionnaire | 2 | 5 | 4 | 4 | 75% |
| Perkins, 2011 | - CTC Web-survey for Agency Directors, Team Members | Questionnaire | 3 | 1 | 4 | 1 | 45% |
|  | - Web-Based Survey for Technical Assistants | Questionnaire | 3 | 2 | 4 | 1 | 50% |
| Sanchez, 2011 | - Coalition Self-Assessment Survey (CSAS) (modified from Kenney & Sofaer, 2000) | Survey | 2 | 3 | 4 | 3 | 60% |
| VanDevanter, 2011 | - Partnership Evaluation Survey Community–Academic Partnership Functioning (modified from Israel et al, 2005) | Survey | 1 | 3 | 4 | 1 | 45% |
| Bilodeau, 2011; Bilodeau, 2019^1^ | - Self-Evaluation Tool for Action in Partnership (Bilodeau et al, 2008, 2014, 2017) French Version, English Version | Questionnaire | 4 | 4 | 4 | 5 | 85% |
| Allen, 2011 | - Online survey (modified from Israel et al, 2005) | Survey | 2 | 4 | 5 | 2 | 65% |
| Curro, 2012 | - Survey | Survey | 2 | 3 | 3 | 2 | 50% |
| El Ansari, 2012 | - Survey | Survey | 4 | 3 | 3 | 3 | 65% |
| CIHR, 2013 McLean, 2012^1^ | - KT-K2A questionnaire | Questionnaire | 2 | 3 | 4 | 3 | 60% |
|  | - KT-PHSI questionnaire | Questionnaire | 2 | 3 | 4 | 3 | 60% |
|  | - KT-synthesis questionnaire | Questionnaire | 2 | 3 | 4 | 3 | 60% |
| Vale, 2012 | - MRC CTU Consumer Involvement Survey | Survey | 0 | 2 | 4 | 4 | 50% |
| Martinez, 2012 | - Protección en Construcción (PenC) Partner Survey (based on Parker et al, 1998) | Survey | 2 | 4 | 5 | 2 | 65% |
| Woodland, 2012 | - Levels of Organizational Integration Rubric (LOIR) (Adapted from Gajda, 2004 and Hogue, 1993 & 1995) | Rubric | 2 | 5 | 4 | 3 | 70% |
|  | - Team - Collaboration Assessment Rubric (TCAR) | Rubric | 0 | 4 | 4 | 3 | 55% |
| Tataw, 2012 | - 17-point assessment tool and framework (Narayan, 1993) | Survey | 2 | 3 | 3 | 1 | 45% |
| Kagan, 2012 | - Site Community Advisory Board (CAB) Survey | Survey | 2 | 4 | 4 | 4 | 70% |
| Brown, 2012 | - CTC Coalition Web-Based Survey | Questionnaire | 3 | 3 | 5 | 2 | 65% |
| Braun, 2012 | - Community Involvement Measure | Questionnaire | 2 | 4 | 2 | 2 | 50% |
| Stedman-Smith, 2012 | - Researcher survey | Survey | 2 | 1 | 4 | 1 | 40% |
|  | - Community Member survey | Survey | 2 | 2 | 4 | 1 | 45% |
| Nargiso, 2013 | - General Coalition Capacities Scale | Scale | 2 | 1 | 4 | 2 | 45% |
|  | - General Coalition Capacity Rubric | Rubric | 2 | 1 | 3 | 2 | 40% |
|  | - Environmental strategy specific capacity rubric | Rubric | 2 | 2 | 3 | 2 | 45% |
| Watson-Thompson, 2013 | - Coalition Process Assessment (Community Toolbox, University of Kansas, 2021) | Checklist | 2 | 4 | 4 | 4 | 70% |
| Khodyakov, 2013 | - Community Engagement in Research Index (CERI) | Index | 4 | 3 | 4 | 3 | 70% |
|  | - 3-model approach  (based on Baker et al, 1999) | Survey | 2 | 3 | 3 | 3 | 55% |
| Patterson, 2014 | - On Becoming a Service User Researcher Survey | Survey | 1 | 2 | 4 | 4 | 55% |
| Rosella, 2018 Rosella, 2014^1^ | - Partnership Self-Assessment Tool (PSAT) (Weiss et al, 2002) | Questionnaire | 3 | 3 | 4 | 5 | 75% |
| Perkins, 2014 | - adapted survey (based on PSAT(S) Cramm et al, 2011; Slaghuis et al, 2011 and Cramm et al, 2013) | Survey | 3 | 2 | 4 | 3 | 60% |
| Chang, 2014 | - Taiwan Health Promotion in Schools (HPS) Support Network Evaluation Study Survey | Survey | 3 | 3 | 3 | 3 | 60% |
| Arroyo-Johnson, 2015 | - Program for the Elimination of Cancer Disparities (PECaD) Collaborative Survey | Survey | 3 | 4 | 5 | 4 | 80% |
|  | - Padres Informados, Jovenes Preparados (PI/PJ) CBPR Survey | Survey | 3 | 4 | 5 | 3 | 75% |
| Brown, 2015 | - CTC Member Coalition Function Survey | Questionnaire | 3 | 3 | 5 | 3 | 70% |
|  | - CTC Functioning Survey (PCCD technical assistance providers) | Questionnaire | 3 | 3 | 4 | 3 | 65% |
|  | - Coalition function survey Additional K (mobilizers, voluntary chairs only) | Questionnaire | 3 | 2 | 5 | 3 | 65% |
|  | - Coalition function Additional L (mobilizers, voluntary chairs only) | Questionnaire | 3 | 2 | 5 | 3 | 65% |
| Gamble, 2015 | - Chief Investigator (CI) Survey | Survey | 1 | 3 | 4 | 3 | 55% |
|  | - Patient and Public Involvement (PPI) Contributor Survey | Survey | 1 | 4 | 4 | 3 | 60% |
| Murphy, 2015 | - The Partnership Assessment Toolkit (PAT) | Toolkit | 2 | 4 | 5 | 5 | 80% |
| Soper, 2015 | - RAND Europe/HERG National CLAHRC Evaluation - online survey | Survey | 2 | 3 | 4 | 5 | 70% |
| Truiett-Theodorson, 2015 | - The Wilder Collaboration Factors Inventory (modified from Mattessich, Murray-Close & Monsey, 2001) | Questionnaire | 1 | 2 | 4 | 2 | 45% |
| Wilson, 2015 | - Online Survey (based on Boote et al, 2006) | Survey | 3 | 4 | 4 | 5 | 80% |
| Bornstein, 2015 | - Member Involvement in Physical Activity Coalitions (MIPAC) Survey | Survey | 4 | 3 | 3 | 4 | 70% |
| Oetzel, 2015 | - Key Informant Survey (KIS) | Survey | 4 | 4 | 4 | 5 | 85% |
|  | - Community Engagement Survey (CES) | Survey | 4 | 4 | 4 | 5 | 85% |
| Oetzel, 2015b | - Community Engagement Survey (CES) | Survey | 4 | 4 | 4 | 5 | 85% |
| Arora, 2015 | - Partnership Assessment in Community Based Research (PAIR) | Questionnaire | 5 | 3 | 5 | 4 | 85% |
| Stocks, 2015 | - Questionnaire (adapted from Morrow et al, 2010) | Questionnaire | 4 | 4 | 5 | 4 | 85% |
| Brown, 2016 | - Coalition Context and Capacity Assessment Scales (from CTC Coalition Web-Based Survey) | Scales | 3 | 3 | 4 | 4 | 70% |
| Gibbons, 2016 | - Brief Online survey | Survey | 1 | 3 | 4 | 3 | 55% |
| Larkan, 2016 | - Towards Successful Global Health Research Partnerships Questionnaire | Questionnaire | 1 | 3 | 3 | 5 | 60% |
| Merkel, 2015 | - Rare Diseases Clinical Research Network Survey (Principal Investigator Version) | Survey | 2 | 3 | 4 | 3 | 60% |
|  | - Rare Diseases Clinical Research Network Survey  (Patient Advocacy Group Member Version) | Survey | 2 | 3 | 4 | 3 | 60% |
| Puyalto, 2016 | - Researcher questionnaire | Questionnaire | 1 | 4 | 3 | 3 | 55% |
|  | - Advisor questionnaire | Questionnaire | 1 | 4 | 4 | 3 | 60% |
| Robbins, 2015 | - Quarterly Survey Questions | Survey | 1 | 4 | 4 | 3 | 55% |
| Finch, 2016 | - Victorian Health Promotion Foundation Partnership Analysis Tool (VPAT) - Checklist (VicHealth, 2016) | Survey | 1 | 4 | 4 | 4 | 65% |
| Goold, 2016 | - DECIDERS Steering Committee Evaluation Questionnaire (adapted from Israel et al, 2013) | Questionnaire | 2 | 4 | 5 | 4 | 75% |
| Abelson, 2016 | Public and Patient Engagement Evaluation Tool (PEET) v1.0 (2015)   - Participant Questionnaire (v1.0 June 2015) | Questionnaire | 3 | 4 | 5 | 5 | 85% |
|  | Public and Patient Engagement Evaluation Tool (PEET) v1.0 (2015)   - Project Questionnaire (v1.0 June 2015) | Questionnaire | 3 | 4 | 5 | 5 | 85% |
|  | Public and Patient Engagement Evaluation Tool (PEET) v1.0 (2015)   - Organization Questionnaire (v1.0 June 2015) | Questionnaire | 3 | 4 | 5 | 5 | 85% |
| Dugan, 2016 | - Program Evaluation Rating Sheet (PERS) | Survey | 1 | 3 | 4 | 4 | 60% |
| Brutt, 2017 | - Perceived Participation  (modified from Pollock et al, 2015) | Questionnaire | 0 | 3 | 2 | 1 | 30% |
| Goodman, 2017 | - Community Engagement Measure | Survey | 5 | 5 | 4 | 4 | 90% |
| Littlecott, 2017 | - Survey | Survey | 2 | 2 | 3 | 4 | 55% |
| Scarinci, 2017 | - Partnership Engagement Survey - Utilization and Satisfaction Questionnaire | Toolkit | 2 | 4 | 5 | 4 | 75% |
| Okazaki, 2017 | - Coordinating Council Member Survey | Survey | 2 | 3 | 3 | 1 | 45% |
| Ray, 2017 | - Stakeholder Survey | Survey | 2 | 4 | 4 | 4 | 70% |
| Weeks, 2017 | - Environmental Scan of Patient and Public Involvement Evaluations in Health Technology Assessment Internationally | Survey | 1 | 3 | 4 | 5 | 65% |
| Jose, 2017 | - Levels of collaboration Survey (Frey et al, 2006) | Survey | 1 | 4 | 3 | 4 | 60% |
|  | - VicHealth Partnership Analysis Tool and Checklist  (VicHealth, 2003) | Survey | 1 | 3 | 3 | 4 | 55% |
| Carroll, 2017 | - Survey | Survey | 3 | 3 | 4 | 4 | 70% |
| Blackburn, 2018 | - Researcher Survey with Quality-Impact Index Score | Questionnaire (with index) | 2 | 3 | 4 | 5 | 70% |
|  | - PPI Contributor Survey with Quality-Impact Index Score | Questionnaire (with index) | 2 | 3 | 4 | 5 | 70% |
| Burrows, 2018 | - Evaluation of SPHERE Advisory Group (Public Advisory Group) | Questionnaire | 3 | 3 | 4 | 4 | 70% |
|  | - Evaluation of SPHERE Public Engagement (Researchers) | Questionnaire | 3 | 3 | 4 | 4 | 70% |
| Forsythe, 2018 | - Annual Report Survey (researchers) | Survey | 2 | 5 | 5 | 5 | 85% |
|  | - PCORI (WE-ENACT) survey (stakeholder partners) | Survey | 2 | 4 | 5 | 5 | 80% |
| Jones, 2018 | - Trust, Mistrust and Power scales from the Partnership Survey | Scales | 4 | 2 | 4 | 3 | 65% |
| Kazmerski, 2018 | - Stakeholder Survey  (modified from Ray & Miller, 2017) | Survey | 1 | 3 | 4 | 2 | 50% |
| Korn, 2018 | - Online Survey  (based on Wallerstein et al, 2008 & 2010, University of New Mexico Center for Participatory Research - CBPR Model, 2017) | Survey | 1 | 1 | 4 | 2 | 40% |
| Mann, 2018 | - PPI contributor questionnaire | Questionnaire | 1 | 3 | 3 | 3 | 50% |
|  | - Researcher questionnaire | Questionnaire | 1 | 3 | 3 | 3 | 50% |
| McIsaac, 2018 | - short online survey (adapted from Skinner et al, 2007) | Survey | 1 | 4 | 4 | 3 | 60% |
| West, 2018 | - Scale of Perceived Trustworthiness | Scale | 4 | 2 | 3 | 3 | 60% |
| Lobo, 2018 | - written response survey | Survey | 0 | 2 | 2 | 3 | 35% |
| Mathie, 2018 | - Completing the Feedback Cycle: Survey of PPI Representatives | Survey | 1 | 3 | 4 | 4 | 60% |
| Oetzel, 2018 | Scales and items from:   - Key Informant Survey (KIS) | Survey | 5 | 4 | 4 | 5 | 90% |
|  | Scales and items from:   - Community Engagement Survey (CES) | Survey | 5 | 4 | 4 | 5 | 90% |
| Nowell, 2018 | - Patient Governor Engagement evaluation (based on PCORI Engagement Rubric, 2014 and PCORI Patient and Family Engagement Rubric, 2014) | Survey | 2 | 3 | 4 | 3 | 60% |
| Kendall, 2018 | - PCORI Data Collection Tool (modified from Forsythe et al, 2016) | Survey | 1 | 3 | 4 | 4 | 60% |
| Walton, 2018 | - GFHS Family Council Meeting Evaluation | Survey | 0 | 4 | 4 | 4 | 60% |
| Hamilton, 2018 | - Patient Engagement in Research Scale (PEIRS) | Scale | 4 | 3 | 5 | 5 | 85% |
| Haynes, 2019 | - Learning Matrix (bespoke to project) - Emoticon survey (based on NHMRC Guidelines for Aboriginal and Torres Strait Islander Health Research, 2003) | Toolkit | 2 | 3 | 4 | 3 | 60% |
| Goodman, 2019 | - Quantitative measure of stakeholder engagement | Survey | 4 | 4 | 4 | 3 | 75% |
| Warner, 2019 | - Active Involvement of Users in Research Observation Schedule and Assessment Questionnaire | Questionnaire | 1 | 2 | 3 | 2 | 40% |
| Ken-Opurum, 2019 | - modified Coalition Self-Assessment Survey (CSAS) (modified from Kenney and Sofaer, 2000) | Survey | 1 | 4 | 5 | 3 | 65% |
| Tabriz, 2019 | - Semi-structured questionnaire | Questionnaire | 0 | 3 | 3 | 2 | 40% |
| Duran, 2019 | - Constructs and sub-constructs (context and partnership processes domains) and outcomes domains from the Community Engagement Survey (CES) | Survey | 5 | 4 | 4 | 5 | 90% |
|  | - Constructs and sub-constructs (context and partnership processes domains) and outcomes domains from the Key Informant Survey (KIS) | Survey | 5 | 4 | 4 | 5 | 90% |
| Lessard, 2019 | - Patient Engagement Satisfaction Survey - Attributed Level of Patient Engagement (International Association of Public Participation’s spectrum of engagement (IAP2), Alberta Health Services, 2012; Kirwan et al, 2017; Bellows et al, 2015) | Toolkit | 1 | 4 | 5 | 2 | 60% |
| Pavarini, 2019 | - anonymous assessment questionnaire | Questionnaire | 1 | 4 | 5 | 2 | 60% |
| Rarere, 2019 | - Evaluation Tool | Survey | 2 | 4 | 4 | 2 | 60% |
| Barger, 2019 | - TrACER Quality of Engagement Survey (External Stakeholder Advisory Group (ESAG) Feedback Survey) | Survey | 0 | 4 | 4 | 3 | 55% |
| Abelson, 2019 | Public and Patient Engagement Evaluation Tool (PPEET) (2018 version)   - Participant Questionnaire (Modules A-B) | Questionnaire | 4 | 4 | 5 | 5 | 90% |
|  | Public and Patient Engagement Evaluation Tool (PPEET) (2018 version)   - Project Questionnaire (Modules A-C) | Questionnaire | 4 | 4 | 5 | 5 | 90% |
|  | Public and Patient Engagement Evaluation Tool (PPEET) (2018 version)   - Organization Questionnaire | Questionnaire | 4 | 4 | 5 | 5 | 90% |
| Roberge-Dao, 2019 | - Survey questionnaire | Questionnaire | 1 | 2 | 4 | 4 | 55% |
| Crocker, 2019 | - Survey | Survey | 3 | 2 | 4 | 5 | 70% |
| Hemphill, 2019 | - PCORI Ways of Engaging‐Engagement Activity Tool (WE‐ENACT) | Survey | 2 | 3 | 5 | 5 | 75% |
| Langlois, 2019 | - self-administered questionnaire | Questionnaire | 1 | 3 | 4 | 3 | 55% |
| Faulkner, 2019 | - Post-Interview Brief Survey | Survey | 1 | 3 | 3 | 4 | 55% |
| Soobiah, 2019 | - modified Patient Engagement Evaluation Tool (PEET) (Moore et al, 2015) | Survey | 4 | 3 | 4 | 4 | 75% |
| Blank, 2019 | - Partnership Evaluation Survey (based on Weiss et al, 2002; Schulz et al, 2003; El Ansari, 2003) | Survey | 2 | 4 | 5 | 4 | 75% |
| Dickson, 2020 | - Items from the Key Informant Survey (E2 KIS) (English and Spanish translation versions) | Survey | 4 | 5 | 4 | 5 | 90% |
| Rodriguez Espinosa, 2020 | - CBPR processes & practices, and outcomes scales (from E2 Key Informant (KIS) and Community-engagement Surveys (CES)) | Scales | 5 | 4 | 4 | 5 | 90% |
| Coombe, 2020 | - Partnership Synergy Questionnaire | Questionnaire | 5 | 4 | 3 | 3 | 75% |
| Snijder, 2010 | - 7-point participation scale  (based on Pretty, 1995; Wagemakers et al, 2008) | Scale | 2 | 3 | 3 | 3 | 55% |
| O’Donovan, 2020 | - Post-Workshop Survey | Questionnaire | 1 | 2 | 4 | 3 | 50% |
|  | - Community Follow-up Assessment | Survey | 1 | 2 | 4 | 3 | 50% |
| Lucero, 2020 | - CBPR Process Scales (synergy, trust, CBPR principles, participation, influence) and Trust Typology (from E2 Community Engagement Survey (CES)) | Scales,  typology | 4 | 3 | 5 | 5 | 85% |
| Spitzer-Shohat, 2020 | - Community Impact of Research Oriented Partnerships (CIROP) Questionnaire | Questionnaire | 4 | 1 | 5 | 5 | 75% |
| Knudsen, 2020 | - coalition member and key stakeholder survey | Survey | 1 | 3 | 4 | 3 | 55% |
| Aguirre, 2020 | - Community Advisory Board Evaluation | Survey | 0 | 4 | 4 | 2 | 50% |
| Hughes, 2020 | - Patient and Public Education Evaluation Tool (PPEET v1.0) - Participant Questionnaire | Questionnaire | 1 | 3 | 5 | 3 | 60% |
| Hinrichsen, 2020 | - Expanded Evaluation Questionnaire (perceived impact) | Questionnaire | 2 | 3 | 5 | 3 | 65% |
| Toledo-Chávarri, 2020 | - Questionnaire (Spanish language) (adapted from Popay, Collins, and the PiiAF Study Group, 2014) | Questionnaire | 2 | 3 | 4 | 5 | 70% |
| Luchtenberg, 2020 | - Feedback Form and Unstructured Oral Evaluation | Toolkit | 0 | 4 | 4 | 2 | 50% |
| Gafos, 2020 | - PROUD Researcher Survey | Survey | 1 | 3 | 4 | 4 | 60% |
|  | - PROUD Community Member Survey | Survey | 1 | 3 | 4 | 4 | 60% |
| Drebit, 2020 | - Public and Patient Engagement Evaluation Tool (PPEET) V2.0 - Organizational Questionnaire | Questionnaire | 3 | 4 | 5 | 5 | 85% |
|  | - Public and Patient Engagement Evaluation Tool (PPEET) V2.0 - Project Questionnaire - Patient Partners Survey | Questionnaire | 3 | 4 | 5 | 5 | 85% |
| Bhati, 2020 | - Web-based evaluation survey (based on Ableson et al, PPEET; PCORI We-ENACT tools) | Survey | 1 | 4 | 5 | 5 | 75% |
| van Schelven, 2020 | Involvement Matrix   - project report - questionnaire | Toolkit | 1 | 4 | 4 | 3 | 60% |
| Haesebaert, 2020 | - Logbooks^a^ - Meeting audiorecordings^a^ - EQUIPPS - GMF Initial Questionnaire for Quality and Patient-Oriented Research (Patient/Caregiver) (French language) - EQUIPPS - GMF Initial Questionnaire for Quality and Patient-Oriented Research (Clinicians/Administrators) (French language) | Toolkit | 2 | 2 | 4 | 5 | 65% |
| Vat, 2020 | - Patients as Partners in Research (patient partner survey) | Survey | 1 | 5 | 5 | 5 | 80% |
|  | - Patients as Partners in Research (researcher survey) | Survey | 1 | 4 | 5 | 5 | 75% |
|  | - Student Survey (adapted from Patient Canada Evaluation Tools and Wilson et al, 2015) | Survey | 1 | 4 | 5 | 5 | 75% |
| Birch, 2020 | - Survey for researchers | Survey | 1 | 3 | 4 | 5 | 65% |
|  | - Survey for Patient Research Partners | Survey | 1 | 3 | 4 | 5 | 65% |
| Seeralan, 2021 | - Public Patient Engagement Evaluation Tool (PPEET) - (German Version) - Patient Questionnaire Module A & B | Questionnaire | 1 | 4 | 5 | 4 | 70% |
|  | - Public Patient Engagement Evaluation Tool (PPEET) - (German Version) - Project Questionnaire Modules A-C | Questionnaire | 1 | 4 | 5 | 4 | 70% |
| Alexander, 2021 | - Community Capacity Evaluation Survey | Survey | 3 | 4 | 5 | 4 | 80% |
| den Houting, 2021 | - Academic and Community Partner Survey | Survey | 2 | 4 | 4 | 5 | 75% |
| Scholz, 2021 | - Consumers as Researchers in Mental Health (CaRiMH) survey | Survey | 1 | 4 | 4 | 4 | 65% |
| van Schelven, 2021 | - Project Outcome Scale | Scale | 3 | 4 | 4 | 3 | 70% |
| Hamilton, 2021 | - Patient Engagement in Research Scale (PEIRS-22 shortened version) (modified from Hamilton et al, 2018) | Scale | 4 | 3 | 3 | 5 | 75% |
| Boursaw, 2021 | - Community Engagement Survey (CES) scales | Scales | 5 | 3 | 4 | 5 | 85% |
| Loban, 2021 | - IMPACT Partnership Questionnaire (Weiss et al, 2002; Jones & Berry, 2011] | Questionnaire | 3 | 2 | 4 | 3 | 60% |
| Martinez, 2021 | - Stakeholder-Centric Engagement Evaluation | Survey | 5 | 4 | 5 | 3 | 85% |
| Moore de Peralta, 2021 | - revised CBPR-PTS instrument (English-Spanish Bilingual Version) | Survey | 4 | 3 | 3 | 4 | 70% |
| Livingstone, 2021 | - Highly Specialized Technologies (HST) Impact Form (v1, v2) | Questionnaire | 1 | 4 | 5 | 4 | 70% |
|  | - Interventional Procedures (IP) Impact Form (v1, v2) | Questionnaire | 1 | 4 | 5 | 4 | 70% |
| March, 2021 | - Stakeholder Survey (adapted from Ray & Miller, 2017 and Kazmerski et al, 2019) | Survey | 1 | 4 | 5 | 2 | 60% |
| Enard, 2021 | - Survey | Survey | 1 | 1 | 4 | 1 | 35% |
| Rasburn, 2021 | - Early engagement survey | Survey | 1 | 3 | 4 | 3 | 55% |
| Knowles, 2021 | - Survey (based on the Generic Learning Outcomes Framework, Arts Council UK) | Survey | 1 | 2 | 2 | 3 | 40% |
| Nunn, 2021 | - Survey | Survey | 2 | 3 | 3 | 4 | 60% |
| Chung, 2021 | - Patient Engagement in Research Scale (PEIRS) instrument | Scale | 4 | 3 | 4 | 5 | 80% |

# **Appendix 9** Synthesis of Future Research Questions

| **Reported Research Questions (n=325)** | **Code Freq (n)** | **Key Themes** |
| --- | --- | --- |
| 1. Evolution of Tools | 80 |  |
| Psychometric testing | 30 | - What is the evidence for tool validity (correlative, face, factor structure, criterion, construct, predictive, discriminant, ecological, among others) and reliability (internal consistency)? - What form should comprehensive psychometric tool development take? - What are the tools’ ceiling/floor effects and responsiveness? - How can tools be applied to predictive modelling? - What considerations are required to understand context and outcomes? - How should the purpose of partnership assessments be balanced with goals and outcomes? - What is the generalizability of tools in other populations? - What is the appropriateness, acceptability, utility, and practicality of the tools? - Can existing tools be shortened? |
| Tool testing | 35 | - To what extent does/should stakeholder involvement in testing, input, and use occur? - Have tools been tested in the same/homogenous populations, and what are partners’ interpretation of the items? - How can tools be tailored to suit different populations? - How can tools be adapted to facilitate cross-cultural use? - How should stakeholders vet items and contexts? - Have tools been field testing with target groups? - Has Delphi testing occurred to develop indicators and validate underlying frameworks? - How can propensity scores be used in larger samples to facilitate causal inference in tool testing? - Have tools been tested in other coalitions, partnerships? - To what extent has tool testing occurred in coalitions with varied geographic, leadership, organizational missions, and models? - Have tools been tested on a broader set of coalitions (i.e., increased number, greater sample size)? |
| Tool and assessment process refinements, adaptations | 11 | - What refinement of measures is required to further develop tools? - What instrument and process development is needed for systematic tool evaluation? - How should existing tools be modified for use in different stages of research? - How can the development of objective, comprehensive measures of partnership functioning be balanced with the comprehensiveness of a tailored approach? - How can tool scale measurement be modified? |
| Role of theory | 1 | - How can we test tools using theory? |
| Tool specific outcomes | 1 | - What is the ability of aggregated scales to measure partners’ perceptions? |
| Translation of tool into other languages | 1 | . |
| Uptake and use of partnership evaluations | 1 | . |
| 1. Partnership measurement and methods | 46 | - Have the partnership research measurement questions been co-prioritized? - Has the planning of PPI evaluation processes occurred a *priori?* - How does a focus on regular monitoring and evaluation of partnerships act as an opportunity for reflection and improvement, facilitate learning and adjustments to partnerships? - How can we better understand the link between partnership plans and real-world actions and shift measurement of perceived outcomes (e.g., self-report) to actual outcomes/impacts (e.g., objective measures) of partnership and partnership process? - How can partnership process variables be integrated and how do we measure of link between coalition process and ultimate outcomes? - How can we statistically test relationships between quantitative indicator data points at each step of the partnership process? - What are ways to seek adequate sample size to refine evaluation of partnership functioning? - What is the role of measurement and regular use of measurement to optimize coalition functioning? - To what extent is co-analysis a mechanism to help prioritize the questions and concerns that are most relevant for stakeholders? - How do we uncover the potential for inherent response bias in assessing engagement? - What new measures for partnership assessment can be developed? - What combination of measures are needed for comprehensive partnership assessment? - How can we best refine tools through psychometric testing (known groups, benchmarks, invariance, subscales)? - How can we refine the synergy process and partnership synergy outcomes and intermediate outcomes? - How can we optimization PPI assessment and the gathering of impact data? - How can we use comparable questionnaires and qualitative data to explore existing and new impacts, and to predict trends for PPI dose-response? - How can we undertake quantitative empirical analysis of participatory program implementation? - What are the ways to embrace mixed methods as a comprehensive evaluation approach? - How can we use qualitative methods to meaningfully compare characteristics of coalitions? - How can we establish minimal outcomes reporting standards, and the standardization of methods to facilitate tool use, as well as definitions for intermediate and long-term outcomes? - How do we better understand the role of and preferences for voting and different voting methods? - What are some effective methods for assessing small sample sizes? - How can we create generalizability through multiple focus groups? - How can we use of longitudinal design with stakeholder reactions and feedback throughout the project? - How to use longitudinal data to facilitate predictive model building? - How to use longitudinal qualitative evaluation to understand stages of partnership development, and to identify triggers of partnership maturity? - What tailored methods and flexible tools can be used to assess partnership, partnership domains, partnership structure and growth, at different stages of partnership maturity and over time? - How can qualitative tools be used to capture the complexity of engagement outcomes? - What are the key characteristics of participatory evaluation? - How can the level and detail of partnership reporting be optimized? - How can the accuracy of reporting the number of stakeholders contributing to partnership assessment responses be improved (stratification)? |
| 1. Investigating engagement (level, type, modality, intensity, optimization) | 39 | - What is the role of personal influence on engagement level? - What are the differentiating characteristics of active vs less active partners (researchers and stakeholders) in a partnership? - What are the effective engagement methods for stakeholders? - How can we better understand the cost-benefits of engagement, and test knowledge transfer and exchange strategies? - What are the intended and unintended consequences of participatory research engagement? - Can we establish optimal engagement levels, the level and degree of engagement by study phases, optimal approaches, and optimal partnership intensity? - What are the required skills and what is optimal engagement, by research phase? - How can stakeholder involvement be optimized over time? - What are the barriers to and facilitators of partnership engagement? - What are the challenges to partnership engagement and disengagement? - What role do partnership goals have in influencing participation? - How can virtual engagement and effectiveness in stakeholder groups be explored? - How can we better understand role duality in PPI? - How can partner non-response be investigated and understood? - What is the influence of multiple stakeholder perspectives in evaluation? - What effect does engagement have on research, by study type? - What is the effectiveness of various partnership research approaches? - How can stakeholders be diversified and evaluated? - How should the perspectives of multiple stakeholder groups, particularly those who have been underrepresented over time be accessed and understood? - How can co-production dynamics be best understood? - How can partnership pathways that enable research uptake be identified? - How can the role and impact of advisory groups be empirically measured? - What is the staff receptivity to increased patient involvement? - How to explore and track Health Technology Assessment engagement strategies and engagement practice standardization? - How can we study the reported intention vs actual participation in future research? - What are the most effective engagement efforts? - What are the best strategies for recruitment and retention of public and patient members? - What is the optimal training and skills development in participatory research, for stakeholders and researchers? - How do we best lever productive tension in new partnerships? |
| 1. Factors influencing partnership optimization (determinants, dose, influences) | 36 | - What are the determinants of- and how do we measure social capital? - How can we Identify and test key factors/co-factors influencing partnership processes, partnership optimization, success, effectiveness, and sustainability? - How can we test theorized factors for successful partnership outcomes? - What is the role of synergy, trust, and decision making in partnership function and ultimate outcomes/impacts? - What is the relative influence of costs and benefits to partnership outcomes and impacts? - How to create an empirical understanding of the relationship between the processes and outcomes of partnership? - What is the influence of partner consensus about partnership vision, coalition definition, on outcomes and impacts? - What influence does social capital have on ultimate partnership outcomes and impacts? - What are the modulating influences of social capital on partnership outcomes and impacts, and on further development of social capital? - What influence does the quality of coalition functioning have on youth outcomes? - What influence does a lack of consensus about coalition definition and its influence on have on partnership outcomes and impacts? - What is the role of partnerships in increasing research evidence uptake and use, and on health and health care improvements? - What is the role of synergy in achieving population health outcomes? - What influence do stakeholder groups have over time on outcomes/impacts? - What is the optimal training required to prepare for successful partnership research, and for developing partnership skills? - What is the optimal training required for researchers? - What influence does external outsourcing of engagement work have to play in partnerships? - What is the relationship between training and level of engagement, training requirements and dose and specificity (by stakeholder group) of training? - What are the correlates of partnership success? - What is the relationship and interaction between multiple antecedents/correlates to partnership function? - What is the optimal dose of technical assistance required for optimized partnership? - What is the optimal dose of engagement to achieve benefits of research partnership? - What are the key constructs related to coalition function? - What are the key capacity constructs of partnership functioning? - What is the empirical evidence for the impact of public involvement in research? - What is the influence of partnerships on intervention process and interventions? - What are the key motivations for involvement in underrepresented groups, and how can these be used as a mechanism for enhancing underrepresented groups’ involvement? - What is the relationship between social capital and empowerment? - How do the benefits of participatory research balance against empowerment and critical consciousness? |
| 1. Optimizing partnership outcomes and impacts | 36 | - How can the measures of capacity related to effective implementation be extended and refined? - How do we Identify the complex paths connecting function to outcome in partnerships? - What are the costs and benefits of partnership and how do they influence outcomes? - How can funding investments be optimized to achieve outcomes/impacts on investment? - What is the impact of coalition capacity on outcomes? - What leadership direction can be derived from observing stakeholders navigating social capital among the partners? - What is the longitudinal impact of action research on ultimate outcomes/impacts? - To what extent do partnerships influence ultimate outcomes and impacts (e.g., patient benefit)? - What is the influence of patient and public involvement on safety, and safety in trials? - What are the costs and economic analysis of PPI models and their relative impact on key research outcomes? - What are the actual vs perceived outcomes and impacts, measured longitudinally? - What are the outcomes of partnership research, from varying stakeholder perspectives? - What is the the wider impact of patient and public involvement, viewed through the systematic assessment of costs-consequences? - How can partnership working be linked to ultimate health and other relevant outcomes? - What is the impact of patient and public involvement on the study? - What contributions does engagement make to culture and researcher development? - What promising partnership practices lead to change? - What are the personal outcomes and antecedents of health research partnerships, and what are the personal outcomes and antecedents of their sustainment? - What causal links can be drawn between personal outcomes and sustainability arising from health research partnerships? - What are the long-term impacts of partnership on health outcomes? - How can we best monitor/evaluate studies to assess cost-effective approaches to partnership? - What sort of study monitoring and assessment of real time impact is required to influence ongoing partnership, facilitate attribution, and optimize impacts? - What influence do partnership approaches have on outcomes and impacts? - What are some key methods for evaluating youth involvement? - What is the influence of partnership on effectiveness of interventions? - How can planning and use of models be used for patient and public involvement, and what are the most effective mechanisms to conduct patient and public involvement? - What are the long-term outcomes and impacts of partnership, and what is the influence of patient and public involvement on outcomes and impacts? |
| 1. Role of partnership in supporting sustainment | 14 | - What personal outcomes and antecedents exist in partnerships, and what is the nature of their sustainment? - What causal link exists between personal outcomes and sustainability? What partnership actions should be undertaken facilitate sustainment (i.e., for partners to retain the vision, to avoid or prevent burn out)? - What is the nature of partnership centrality? - What are the interorganizational linkages in partnerships and how do these relate to coalition sustainability? - What is the combined effect of partnership on coalition sustainability? - What are the effects of the intensity and type of facilitation on coalition function and sustainability? - What is the role of partnership function in attaining synergy and testing models of coalition functioning, to optimize sustainability? - What is the role of synergy in supporting sustainability? - What are the key constructs that support development of synergy and sustainability? - What are the predictors of sustainability that can be attained through team functioning and sustainability planning? - What are the key factors that facilitate and hinder sustainability and scale? |
| 1. Comparative effectiveness of partnership approaches | 12 | - What is the effectiveness of a coalition approach compared to other research partnership approaches? - What is the cost effectiveness of a coalition (i.e., form, type, and circumstances to optimize partnership) as compared with other partnership approaches? - What is the comparative effectiveness of varied technical assistance models (i.e., varied by funding, intensity)? - What is the comparative role of context among different partnership cases? What does the role of context contribute to our understanding of authentic partnership? - What are the various, effective methods to assess partnership? - What is the comparative effectiveness of impact measurement among different and meaningful patient and public involvement approaches? - How do approaches vary across populations and contexts, in which specific ways? - How do engagement methods compare and how does this comparison contribute to partnership study optimization? |
| 1. Use of theory | 8 | - How is theory used to guide evaluation, define goals and success? - How are theory-based approaches used to better understand the influence of partnerships? - How can we expand and test conceptual frameworks, and their underlying principles? - How can we expand evaluation theory and methods in PPI evaluation? - How can frameworks be used to create a clear lens for research, evaluation, and reporting of partner engagement? |
| 1. Evolution of partnerships over time | 7 | - How do partnerships change over time and what are the key differences between higher and lower functioning coalitions? - How can longitudinal measurement be used to reveal how, when and which ways partnerships evolve? - How can we shift in investigator disposition and practices over time (i.e., from transactional to relational functioning)? - How does impact change over time, observed through the lens of the long-term case study? - What are the ways we can shift partnership practice towards the inclusion of partners across multiple phases of research, as mandated by funders? |
| 1. Role of leadership in partnerships | 7 | - What is the influence of social capital on leadership and skills development? - What is the effect of paid leadership on coalition function and effectiveness? - What skills are required of partnership leaders, and what is their influence on intermediate and ultimate outcomes and impacts? - What is the role of leadership and championship in supporting partnerships? - How can longitudinal design be leveraged to explore optimization of leadership and teamwork in partnerships? |
| 1. Role of context in partnership | 6 | - What is the influence of a fluctuating environment on social capital? - What is the stability of social capital over time? - What is the role of context in modulating the relationship between participation and personal influence within partnerships? - What is the role of context and its contribution to theory in health research partnerships? - What are the key contextual constructs influencing partnership function? |
| 1. Optimizing implementation | 5 | - How can rigor be balanced with relevance to improve applicability of partnerships? - How should implementation be tailored to retain fidelity? - What are the mechanisms that promote research use? - What are the key actions that support implementation and impact on policy, practice? - How can partnership methods and efforts be trained on the improvement of ultimate impacts? |
| 1. Addressing priority populations and their concerns through partnership research approaches | 5 | - How do we best optimize involvement of youth in research? - How can we address lateral violence, and how might the issue be addressed using participatory approaches? - What are the potential empowerment aspects of tools and how can these be further developed and assessed? - How can we use respondent-driven sampling to facilitate contact with hard-to-reach populations in partnership research? |
| 1. Miscellaneous | 5 | - How can web-based data capture and data linkage be used to assess coalitions? - What is the role of academics in real-world community needs assessment and intervention? - What influence do partnership approaches have in different forms of health research? - What is the role of boundary spanners in partnership research? - How can organization-based participatory research be applied to other fields and purposes? |
| 1. Quality improvement of partnership evaluation process | 4 | - How can insights from partners be sought regarding the evaluation process itself to improve upon it from both process and methods angles? - How can evaluation approaches and experiences be shared and exchanged? - What are the effective ways to create implementation efficiency in a coalition? - What do we know about workflow efficiency in health research partnerships? |
| 1. Indicators | 3 | - What are the existing and new indicators of community engagement? - What are the existing and new indicators of engagement impact? - How can process modelling be used within and among partnership cases to identify new or better measures of engagement? |
| 1. Conceptual clarity | 2 | - What is the full scope for both costs and benefits concepts? - How can we test models of collaboration and what do these tests tell us about the collaboration concept? |
| 1. Structural barriers to partnership | 2 | - What institutional policies and guidelines exist that hinder partnerships? - What are the barriers to/facilitators of partnership implementation and how can these be targeted to optimize partnership implementation? |
| 1. Role of funders | 2 | - What is the role of funders in monitoring partnership implementation, in creating change over time, and in impact evaluation? - How can funding be studied as a mechanism for enhancing meaningful involvement and impact? |
| 1. Role of governance and management on partnership success | 2 | - What is the role of management and governance on optimization of partnership outcomes and impacts? - How are governance structure and process best understood within health research partnerships? |
| 1. Understanding community development to optimize partnership success | 2 | - How can we optimize partnership success? - How can community capacity be optimized across partnerships, and how does community capacity contribute to outcomes and impacts? |
| 1. Role and influence of partnership standards | 1 | - What influence do partnership standards have and what is the uptake of standards in partnerships? |
| 1. Role of HCP in facilitating program effectiveness through partnerships | 1 | - What are the health and program effectiveness outcomes? - What roles do health care providers play in leading, and facilitating partnership? |

# **Appendix 10** Synthesis of Evidence Gaps

| **Reported Research Gaps**  **(n=19)** | **Code Frequ (n)** | **Key Themes** |
| --- | --- | --- |
| Refined understanding of engagement levels, timing among stakeholders | 6 | - Encourage researcher-stakeholder relationship formation - Engage stakeholders early and across multiple phases - Tailor engagement to stakeholder groups - Tension between stakeholder representativeness and importance of the presence of stakeholder voice |
| Support teams working to integrate stakeholder partnership | 5 | - Identify and provide supports for partnership - Create policy to support partnership - Focus on studies with greatest impact potential for embedding and realizing partnership - Need guidance to assess acceptability, applicability, cost-effectiveness of stakeholder involvement to tailor partnership approach, and to assess impact - Support researcher learning |
| Objective metrics required | 2 | - Need objective metrics for research outcomes and impacts; self-reported metrics are subjective |
| Conceptual underpinnings | 2 | - Need conceptual frameworks and models to understand and act to improve partnerships - Need to contextualize non-health domain frameworks |
| Structures supporting PPI | 2 | - Need governance to ensure stakeholders are embedded and team has access to stakeholder voices - Need to enable stakeholder-led research |
| Unpacking partnership science | 1 | - Need to uncover contexts and mechanisms of engagement as a gateway to understanding impact |
| Health research strengthening | 1 | - Need to build capacity for systems thinking |

# **Appendix 11** Synthesis of Recommendations

| **Recommendation Category**  **(n=54)** | **Code Freq (n)** | **Key Themes** |
| --- | --- | --- |
| Supporting research partnerships, structural supports for partnerships | 26 | - Capacity building in patients and staff for stakeholder engagement and for external stakeholders (e.g., research methods) - Supply resources for facilitating PI and capacity building (e.g., resources, software) - Dissemination supports and dissemination ‘detailing’ to facilitate uptake - Funder expectations about organizational infrastructure and project-specific capacities to support PPI and ensure impact and relevance - Funder provision of resources directed deliberately at partnership research - Funder revision of structures to support advisory group structures and facilitating factors for participatory research - Funders monitor PPI activity within research projects and help researchers to make realistic plans for PPI at the outset - Focused investment to overcome systemic barriers to participatory research - Changes to funding structures - Structure explicit objectives for PPI early in the process to achieve productivity and other goals - Consider key factors to assess readiness/feasibility prior to partnership establishment - PPI planning as an integral part of the evaluation design - Prioritizing partnership function will facilitate the effectiveness of the partnership - Platform for community agencies to collectively discuss and make decisions - Remove boundaries to facilitate communication, collaborative design, and outcomes - Bottom-up approach requires a structural shift by funders to allow changes and developments in collaborative objectives and activities post-award - Move from inclusion to co-design in order to maximize patients’ and families’ ability to share insights and assure satisfaction with the process - Co-building evidence is only one aspect – embedding the tools thoughtfully into the care trajectory and patient flow is critical and requires additional and continued stakeholder involvement - Advancing theory and practice through authentic collaboration must be integrated into tenure and promotion guidelines and faculty reporting - Collective focus on the ultimate impacts of the target population(s) |
| Engagement level and timing for different stakeholders | 15 | - Stakeholder disposition (planning, motivation, ongoing and new work) - Balance must be struck between exploration and actionable implementation results and policy improvements - Broadening stakeholder complement ensures relevance and enhances impact - Collective priority setting, focus on action and intervention - Active 2-way brokering and engagement required to build opportunities |
| Sustainability planning | 5 | - Sustainability planning is critical regardless of the nature of the research contribution by considering the boundaries of scalability of the intervention including topic, benefits, attitudes, networks, leadership, policy articulation and integration, financial and political factors - Continued implementation requires attention to key sustainability factors, including training and resources |
| Primary research on partnership approaches | 4 | - Greater focus on context to facilitate uptake and viability of partnership - Planning and prioritization algorithm for deciding on how much engagement (dose), methods, objectives, and resources - Standardization of engagement in partnerships - Integration of narrative methods - Integration of ongoing monitoring and evaluation of partnerships - Build an evidence base to codify best practices in partnership research |
| Terminology | 3 | - Differentiating terms is a requirement for measurement - Clear definition of consumer and consumer involvement is required for all stakeholders |
| Rigorous evaluation of partnerships | 1 | - Patient centered practice and comprehensive evaluations facilitate understanding of process and outcomes and realize impacts beyond the project |

# **Appendix 12** Bibliography of included studies

| **Included Papers (n=169: n=166 studies with 3 companion reports)** |
| --- |
| 1. Butterfoss FD, Goodman RM, Wandersman A. Community coalitions for prevention and health promotion: factors predicting satisfaction, participation, and planning. Health Education Quarterly. 1996;23(1):65-79. |
| 1. Goodman RM, Wandersman A, Chinman M, Imm P, Morrissey E. An ecological assessment of community-based interventions for prevention and health promotion: approaches to measuring community coalitions. Am J Comm Psyc. 1996;24(1):33-61. |
| 1. Goldstein SM. Community coalitions: a self-assessment tool. American Journal of Health Promotion. 1997;11(6):430-435. |
| 1. Kegler MC, Steckler A, McLeroy K, Malek SH. Factors that contribute to effective community health promotion coalitions: a study of 10 Project ASSIST coalitions in North Carolina. Health Education & Behavior. 1998;25(3):338-353. |
| 1. Armbruster C, Gale B, Brady J, Thompson N. Perceived ownership in a community coalition. Public Health Nursing. 1999;16(1):17-22. |
| 1. Chan B, Bazzoli G, Shortell SM, Hasnain-Wynia R. A social capital index for community partnerships. International Quarterly of Community Health Education. 2000;20(3):213-235. |
| 1. Lantz PM, Viruell-Fuentes E, Israel BA, Softley D, Guzman R. Can communities and academia work together on public health research? Evaluation results from a community-based participatory research partnership in Detroit. Journal of Urban Health. 2001;78(3):495-507. |
| 1. Shortell SM, Zukoski AP, Alexander JA, et al. Evaluating partnerships for community health improvement: tracking the footprints. J Health Politics, Policy & Law. 2002;27(1):49-91. |
| 1. Weiss ES, Anderson RM, Lasker RD. Making the most of collaboration: exploring the relationship between partnership synergy and partnership functioning. Health Education & Behavior. 2002;29(6):683-698. |
| 1. Schulz AJ, Israel BA, Lantz P. Instrument for evaluating dimensions of group dynamics within community-based participatory research partnerships. Evaluation and Program Planning. 2003;26(3):249-262. |
| 1. Cotter JJ, Welleford EA, Vesley-Massey K, Thurston MO. Collaborative community-based research and innovation. Family & Community Health. 2003;26(4):329-337. |
| 1. Butterfoss FD. The coalition technical assistance and training framework: helping community coalitions help themselves. Health Promotion Practice. 2004;5(2):118-126. |
| 1. El Ansari W, Phillips CJ. The costs and benefits to participants in community partnerships: a paradox? Health Promotion Practice. 2004;5(1):35-48. |
| 1. El Ansari W, Phillips CJ, Zwi AB. Public health nurses' perspectives on collaborative partnerships in South Africa. Public Health Nursing. 2004;21(3):277-286. |
| 1. Metzger ME, Alexander JA, Weiner BJ. The effects of leadership and governance processes on member participation in community health coalitions. Health Education & Behavior. 2005;32(4):455-473. |
| 1. Kramer JS, Philliber S, Brindis CD, et al. Coalition models: lessons learned from the CDC's Community Coalition Partnership Programs for the Prevention of Teen Pregnancy. Journal of Adolescent Health. 2005;37(3 Suppl):S20-30. |
| 1. Kegler MC, Williams CW, Cassell CM, et al. Mobilizing communities for teen pregnancy prevention: associations between coalition characteristics and perceived accomplishments. Journal of Adolescent Health. 2005;37(3 Suppl):S31-41. |
| 1. Cramer ME, Atwood JR, Stoner JA. Measuring community coalition effectiveness using the ICE instrument. Public Health Nursing. 2006;23(1):74-87. |
| 1. Savitz LA. Managing effective participatory research partnerships. Joint Commission Journal on Quality & Patient Safety. 2007;33(12):7-15. |
| 1. Barber R, Boote JD, Cooper CL. Involving consumers successfully in NHS research: a national survey. Health Expectations. 2007;10(4):380-391. |
| 1. Feinberg ME, Bontempo DE, Greenberg MT. Predictors and level of sustainability of community prevention coalitions. American Journal of Preventive Medicine. 2008;34(6):495-501. |
| 1. Feinberg ME, Gomez BJ, Puddy RW, Greenberg MT. Evaluation and community prevention coalitions: validation of an integrated Web-based/technical assistance consultant model. Health Education & Behavior. 2008;35(1):9-21. |
| 1. Wyatt K, Carter M, Mahtani V, Barnard A, Hawton A, Britten N. The impact of consumer involvement in research: an evaluation of consumer involvement in the London Primary Care Studies Programme. Family Practice. 2008;25(3):154-161. |
| 1. Lovell SA. Engaging communities in health geography? Assessing the strategy of community-based participatory research, ProQuest Information & Learning; 2008. |
| 1. Garner L. Evaluation of a youth substance abuse prevention coalition: A case study of a community-initiated model, ProQuest Information & Learning; 2008. |
| 1. Orr Brawer CR. Replication of the value template process in a community coalition: Implications for social capital and sustainability, ProQuest Information & Learning; 2008. |
| 1. Adily A, Black D, Graham ID, Ward JE. Research engagement and outcomes in public health and health services research in Australia. Australian & New Zealand Journal of Public Health. 2009;33(3):258-261. |
| 1. King G, Servais M, Kertoy M, et al. A measure of community members' perceptions of the impacts of research partnerships in health and social services. Evaluation & Program Planning. 2009;32(3):289-299. |
| 1. Van Olphen J, Ottoson J, Green L, Barlow J, Koblick K, Hiatt R. Evaluation of a partnership approach to translating research on breast cancer and the environment. Progress in Community Health Partnerships. 2009;3(3):213-226. |
| 1. Sunderland N, Domalewski D, Kendall E, Armstrong K. Which comes first: the partnership or the tool? Reflections on the effective use of partnership tools in local health partnerships. Australian Journal of Primary Health. 2009;15(4):303-311. |
| 1. Tolma EL, Cheney MK, Troup P, Hann N. Designing the process evaluation for the collaborative planning of a local turning point partnership. Health Promotion Practice. 2009;10(4):537-548. |
| 1. Barnidge EK, Brownson CA, Baker EA, Shetty G. Tools for building clinic-community partnerships to support chronic disease control and prevention. Diabetes Educator. 2010;36(2):190-201. |
| 1. Blevins D, Farmer MS, Edlund C, Sullivan G, Kirchner JE. Collaborative research between clinicians and researchers: a multiple case study of implementation. Implementation Science. 2010;5:76. |
| 1. Wagemakers A, Koelen MA, Lezwijn J, Vaandrager L. Coordinated action checklist: a tool for partnerships to facilitate and evaluate community health promotion. Global Health Promotion. 2010;17(3):17-28. |
| 1. King G, Servais M, Forchuk C, et al. Features and impacts of five multidisciplinary community-university research partnerships. Health & Social Care in the Community. 2010;18(1):59-69. |
| 1. Wright D, Foster C, Amir Z, Elliott J, Wilson R. Critical appraisal guidelines for assessing the quality and impact of user involvement in research. Health Expectations. 2010;13(4):359-368. |
| 1. Ziff MA, Willard N, Harper G. Connect to Protect Researcher Community Partnerships: Assessing Change in Successful Collaboration Factors over Time. Global Journal of Community Psychology Practice, 2010, 1(1): 32-39. |
| 1. Raine KD, Plotnikoff R, Nykiforuk C, et al. Reflections on community-based population health intervention and evaluation for obesity and chronic disease prevention: the Healthy Alberta Communities project. International Journal of Public Health. 2010;55(6):679-686. |
| 1. Jones J, Barry MM. Developing a scale to measure synergy in health promotion partnerships. Global Health Promotion. 2011;18(2):36-44. |
| 1. Jones J, Barry M. Developing a scale to measure trust in health promotion partnerships. Health Promot Int. 2011 Dec;26(4):484-91. doi: 10.1093/heapro/dar007. Epub 2011 Feb 20. |
| 1. Payne JM, D'Antoine HA, France KE, et al. Collaborating with consumer and community representatives in health and medical research in Australia: results from an evaluation. Health Research Policy & Systems. 2011;9:18. |
| 1. Perkins DF, Feinberg ME, Greenberg MT, et al. Team factors that predict to sustainability indicators for community-based prevention teams. Evaluation & Program Planning. 2011;34(3):283-291. |
| 1. Sanchez V, Carrillo C, Wallerstein N. From the ground up: building a participatory evaluation model. Progress in Community Health Partnerships. 2011;5(1):45-52. |
| 1. VanDevanter N, Kwon S, Sim SC, Chun K, Coalition BFC, Trinh-Shevrin C. Evaluation of community-academic partnership functioning: center for the elimination of hepatitis B health disparities. Progress in Community Health Partnerships. 2011;5(3):223-233. |
| 1. Bilodeau A, Galarneau M, Fournier M, et al. L'Outil diagnostique de l'action en partenariat: fondements, élaboration et validation. Canadian Journal of Public Health. 2011;102(4):298-302.   English translation accompanying report:  Bilodeau A, Kranias G. Self-Evaluation Tool for Action in Partnership: Translation and Cultural Adaptation of the Original Quebec French Tool to Canadian English. Canadian Journal of Program Evaluation, 2019, 34(2): 192-206 doi 10.3138/cjpe.43685 |
| 1. Allen ML, Culhane-Pera KA, Pergament S, Call KT. A capacity building program to promote CBPR partnerships between academic researchers and community members. Clinical and translational science. 2011;4(6):428-433. |
| 1. Curro FA, Thompson VP, Grill A, et al. An assessment of the perceived benefits and challenges of participating in a practice-based research network. Primary Dental Journal. 2012;1(1):50-57. |
| 1. El Ansari W. Leadership in community partnerships: South African study and experience. Central European Journal of Public Health. 2012;20(3):174-184. |
| 1. Protocol superseded by report: CIHR Evaluation Unit, Resource Planning and Management Portfolio. Evaluation of CIHR's Knowledge Translation Funding Program - Evaluation Report 2013. 95pg   Original Citation (Protocol – accompanying report): McLean RKD, Graham ID, Bosompra K, et al. Understanding the performance and impact of public knowledge translation funding interventions: protocol for an evaluation of Canadian Institutes of Health Research knowledge translation funding programs. Implementation Science. 2012;7:57. (PROTOCOL) |
| 1. Vale CL, Thompson LC, Murphy C, Forcat S, Hanley B. Involvement of consumers in studies run by the Medical Research Council Clinical Trials Unit: results of a survey. Trials [Electronic Resource]. 2012;13:9. |
| 1. Martinez LS, Ndulue UJ, Brunette MJ. Lessons learned from the protección en Construcción (PenC) community research partnership. International Public Health Journal. 2012;4(3):275-283. |
| 1. Woodland RH, Hutton MS. Evaluating organizational collaborations: Suggested entry points and strategies. American Journal of Evaluation. 2012;33(3):366-383. |
| 1. Tataw DB. Toward a Horizontal Participatory Implementation Approach for Community Health Programs Serving Vulnerable Populations. Journal of Human Behavior in the Social Environment. 2012;22(4):421-435. |
| 1. Kagan JM, Rosas SR, Siskind RL, et al. Community-researcher partnerships at NIAID HIV/AIDS clinical trials sites: insights for evaluation and enhancement. Progress in Community Health Partnerships. 2012;6(3):311-320. |
| 1. Brown LD, Feinberg ME, Greenberg MT. Measuring Coalition Functioning: Refining Constructs through Factor Analysis. Health Educ Behav. 2012 August ; 39(4): 486–497. doi:10.1177/1090198111419655. |
| 1. Braun KL, Nguyen TT, Tanjasiri SP, et al. Operationalization of community-based participatory research principles: assessment of the national cancer institute's community network programs. American Journal of Public Health. 2012;102(6):1195-1203. |
| 1. Stedman-Smith M, McGovern PM, Peden-McAlpine CJ, Kingery LR, Draeger KJ. Photovoice in the Red River Basin of the north: a systematic evaluation of a community-academic partnership. Health Promotion Practice. 2012;13(5):599-607. |
| 1. Nargiso JE, Friend KB, Egan C, et al. Coalitional capacities and environmental strategies to prevent underage drinking. American Journal of Community Psychology. 2013;51(1-2):222-231. |
| 1. Watson-Thompson J, Woods NK, Schober DJ, Schultz JA. Enhancing the capacity of substance abuse prevention coalitions through training and technical assistance. Journal of Prevention & Intervention in the Community. 2013;41(3):176-187. |
| 1. Khodyakov D, Stockdale S, Jones A, Mango J, Jones F, Lizaola E. On measuring community participation in research. Health Education & Behavior. 2013;40(3):346-354. |
| 1. Patterson S, Trite J, Weaver T. Activity and views of service users involved in mental health research: UK survey. British Journal of Psychiatry. 2014;205(1):68-75. |
| 1. Protocol superseded by report: Rosella L, Bornbaum C, Kornas K, et al. Evaluating the process and outcomes of a knowledge translation approach to supporting use of the Diabetes Population Risk Tool (DPoRT) in public health practice. 2018, 33(1): 21-48. Original Citation (Protocol – accompanying report): Rosella L, Peirson L, Bornbaum C, et al. Supporting collaborative use of the Diabetes Population Risk Tool (DPoRT) in health-related practice: a multiple case study research protocol. Implementation Science. 2014;9(1):35. |
| 1. Perkins C-TM. Partnership functioning and sustainability in nursing academic practice partnerships: The mediating role of partnership synergy, University of Northern Colorado; 2014. |
| 1. Chang FC, Liu CH, Liao LL, et al. Facilitating the implementation and efficacy of health-promoting schools via an action-research approach in Taiwan. Health Promotion International. 2014;29(2):306-316. |
| 1. Arroyo-Johnson C, Allen ML, Colditz GA, et al. A Tale of Two Community Networks Program Centers: Operationalizing and Assessing CBPR Principles and Evaluating Partnership Outcomes. Progress in Community Health Partnerships. 2015;9 Suppl:61-69. |
| 1. Brown LD, Feinberg ME, Shapiro VB, Greenberg MT. Reciprocal relations between coalition functioning and the provision of implementation support. Prevention Science. 2015;16(1):101-109. |
| 1. Gamble C, Dudley L, Allam A, et al. An evidence base to optimize methods for involving patient and public contributors in clinical trials: a mixed-methods study. NIHR Journals Library Health Services and Delivery Research. 2015:09. |
| 1. Murphy J, Hatfield J, Afsana K, Neufeld V. Making a commitment to ethics in global health research partnerships: a practical tool to support ethical practice. Journal of Bioethical Inquiry. 2015;12(1):137-146. |
| 1. Soper B, Hinrichs S, Drabble S, et al. Delivering the aims of the Collaborations for Leadership in Applied Health Research and Care: understanding their strategies and contributions. NIHR Journals Library Health Services and Delivery Research. 2015:05. |
| 1. Truiett-Theodorson R, Tuck S, Bowie JV, Summers AC, Kelber-Kaye J. Building effective partnerships to improve birth outcomes by reducing obesity: The B'more Fit for healthy babies coalition of Baltimore. Evaluation & Program Planning. 2015;51:53-58. |
| 1. Wilson P, Mathie E, Keenan J, et al. ReseArch with Patient and Public invOlvement: a RealisT evaluation – the RAPPORT study. NIHR Journals Library Health Services and Delivery Research. 2015:09. |
| 1. Bornstein DB, Pate RR, Beets MW, Ortaglia A, Saunders RP, Blair SN. New Perspective on Factors Related to Coalition Success: Novel Findings From an Investigation of Physical Activity Coalitions Across the United States. Journal of Public Health Management & Practice. 2015;21(6):E23-30. |
| 1. Oetzel JG, Villegas M, Zenone H, White Hat ER, Wallerstein N, Duran B. Enhancing stewardship of community-engaged research through governance. American Journal of Public Health. 2015;105(6):1161-1167. |
| 1. Oetzel JG, Zhou C, Duran B, et al. Establishing the psychometric properties of constructs in a community-based participatory research conceptual model. American Journal of Health Promotion. 2015;29(5):e188-202. |
| 1. Arora PG, Krumholz LS, Guerra T, Leff SS. Measuring Community-Based Participatory Research Partnerships: The Initial Development of an Assessment Instrument. Progress in Community Health Partnerships. 2015;9(4):549-560. |
| 1. Stocks SJ, Giles SJ, Cheraghi-Sohi S, Campbell SM. Application of a tool for the evaluation of public and patient involvement in research. BMJ Open. 2015;5(3):e006390. |
| 1. Brown LD, Chilenski SM, Ramos R, Gallegos N, Feinberg ME. Community Prevention Coalition Context and Capacity Assessment: Comparing the United States and Mexico. Health Education & Behavior. 2016;43(2):145-155. |
| 1. Gibbons MC, Illangasekare SL, Smith E, Kub J. A Community Health Initiative: Evaluation and Early Lessons Learned. Progress in Community Health Partnerships. 2016;10(1):89-101. |
| 1. Larkan F, Uduma O, Lawal SA, van Bavel B. Developing a framework for successful research partnerships in global health. Global Health. 2016;12(1):17. |
| 1. Merkel PA, Manion M, Gopal-Srivastava R, et al. The partnership of patient advocacy groups and clinical investigators in the rare diseases clinical research network. Orphanet Journal Of Rare Diseases. 2016;11(1):66. |
| 1. Puyalto C, Pallisera M, Fullana J, Vila M. Doing Research Together: A Study on the Views of Advisors with Intellectual Disabilities and Non-Disabled Researchers Collaborating in Research. Journal of Applied Research in Intellectual Disabilities. 2016;29(2):146-159. |
| 1. Robbins M, Tufte J, Hsu C. Learning to "Swim" with the Experts: Experiences of Two Patient Co-Investigators for a Project Funded by the Patient-Centered Outcomes Research Institute. Permanente Journal. 2016;20(2):85-88. |
| 1. Finch CF, Donaldson A, Gabbe BJ, et al. The evolution of multiagency partnerships for safety over the course of research engagement: experiences from the NoGAPS project. Injury Prevention. 2016;22(6):386-391. |
| 1. Goold S, Rowe Z, Calhoun K, et al. The State as Community in Community-Based Participatory Research. Progress in Community Health Partnerships. 2016;10(4):515-522. |
| 1. Abelson J, Li K, Wilson G, Shields K, Schneider C, Boesveld S. Supporting quality public and patient engagement in health system organizations: development and usability testing of the Public and Patient Engagement Evaluation Tool. Health Expectations. 2016;19(4):817-827. |
| 1. Dugan AG, Farr DA, Namazi S, et al. Process evaluation of two participatory approaches: Implementing total worker health interventions in a correctional workforce. American Journal of Industrial Medicine. 2016;59(10):897-918. |
| 1. Brutt AL, Meister R, Bernges T, et al. Patient involvement in a systematic review: Development and pilot evaluation of a patient workshop. Zeitschrift fur Evidenz Fortbildung und Qualitat im Gesundheitswesen. 2017;127-128:56-61. |
| 1. Goodman MS, Sanders Thompson VL, Johnson CA, et al. Evaluating Community Engagement in Research: Quantitative Measure Development. Journal of Community Psychology. 2017;45(1):17-32. |
| 1. Littlecott HJ, Fox KR, Stathi A, Thompson JL. Perceptions of success of a local UK public health collaborative+. Health Promotion International. 2017;32(1):102-112. |
| 1. Scarinci IC, Moore A, Benjamin R, Vickers S, Shikany J, Fouad M. A participatory evaluation framework in the establishment and implementation of transdisciplinary collaborative centers for health disparities research. Evaluation & Program Planning. 2017;60:37-45. |
| 1. Okazaki S, Wong SN, Kaplan BL. Strategic collaborative partnerships to improve immigrant Chinese community health: A case study. Asian American Journal of Psychology. 2017;8(4):339-350. |
| 1. Ray KN, Miller E. Strengthening stakeholder-engaged research and research on stakeholder engagement. Journal of Comparative Effectiveness Research. 2017;6(4):375-389. |
| 1. Weeks L, Polisena J, Scott AM, Holtorf AP, Staniszewska S, Facey K. Evaluation of Patient and Public Involvement Initiatives in Health Technology Assessment: A Survey of International Agencies. International Journal of Technology Assessment in Health Care. 2017;33(6):715-723. |
| 1. Jose K, Venn A, Jarman L, et al. Partnering Healthy@Work: an Australian university-government partnership facilitating policy-relevant research. Health Promotion International. 2017;32(6):964-976. |
| 1. Carroll SL, Embuldeniya G, Abelson J, McGillion M, Berkesse A, Healey JS. Questioning patient engagement: research scientists' perceptions of the challenges of patient engagement in a cardiovascular research network. Patient preference & adherence. 2017;11:1573-1583. |
| 1. Blackburn S, McLachlan S, Jowett S, et al. The extent, quality, and impact of patient and public involvement in primary care research: a mixed methods study. Research Involvement & Engagement. 2018;4:16. |
| 1. Burrows A, Meller B, Craddock I, Hyland F, Gooberman-Hill R. User involvement in digital health: Working together to design smart home health technology. Health Expectations. 2018:05. |
| 1. Forsythe L, Heckert A, Margolis MK, Schrandt S, Frank L. Methods and impact of engagement in research, from theory to practice and back again: early findings from the Patient-Centered Outcomes Research Institute. Quality of Life Research. 2018;27(1):17-31. |
| 1. Jones J, Barry MM. Factors influencing trust and mistrust in health promotion partnerships. Global Health Promotion. 2018;25(2):16-24. |
| 1. Kazmerski TM, Miller E, Sawicki GS, et al. Developing Sexual and Reproductive Health Educational Resources for Young Women with Cystic Fibrosis: A Structured Approach to Stakeholder Engagement. The Patient: Patient Centered Outcomes Research. 2018:26. |
| 1. Korn AR, Hennessy E, Tovar A, Finn C, Hammond RA, Economos CD. Engaging Coalitions in Community-Based Childhood Obesity Prevention Interventions: A Mixed Methods Assessment. Childhood Obesity. 2018:06. |
| 1. Mann C, Chilcott S, Plumb K, Brooks E, Man MS. Reporting and appraising the context, process, and impact of PPI on contributors, researchers, and the trial during a randomized controlled trial - the 3D study. Research Involvement & Engagement. 2018;4:15. |
| 1. McIsaac JLD, Penney TL, Storey KE, et al. Integrated knowledge translation in population health intervention research: a case study of implementation and outcomes from a school-based project. Health Research Policy & Systems. 2018;16(1):72. |
| 1. West KM. Researcher trustworthiness in community-academic research partnerships: Implications for genomic research, ProQuest Information & Learning; 2018. |
| 1. Lobo R, Crawford G, Hallett J, et al. A research and evaluation capacity building model in Western Australia. Health Promotion International. 2018;33(3):468-478. |
| 1. Mathie E, Wythe H, Munday D, et al. Reciprocal relationships and the importance of feedback in patient and public involvement: A mixed methods study. Health Expectations. 2018;21(5):899-908. |
| 1. Oetzel JG, Wallerstein N, Duran B, et al. Impact of Participatory Health Research: A Test of the Community-Based Participatory Research Conceptual Model. BioMed Research International. 2018;2018:7281405. |
| 1. Nowell WB, Curtis JR, Crow-Hercher R. Patient Governance in a Patient-Powered Research Network for Adult Rheumatologic Conditions. Medical Care. 2018;56 Suppl 10 Suppl 1:S16-S21. |
| 1. Kendall, C., et al. (2018). ""Still learning and evolving in our approaches": patient and stakeholder engagement among Canadian community-based primary health care researchers." Research Involvement & Engagement 4: 47. |
| 1. Walton, K., et al. (2018). "Putting family into family-based obesity prevention: enhancing participant engagement through a novel integrated knowledge translation strategy." BMC Medical Research Methodology 18(1): 126. |
| 1. Hamilton, C. B., et al. (2018). "Development and pre-testing of the Patient Engagement In Research Scale (PEIRS) to assess the quality of engagement from a patient perspective." PLoS ONE [Electronic Resource] 13(11): e0206588. |
| 1. Haynes, E., et al. (2019). "Community-based participatory action research on rheumatic heart disease in an Australian Aboriginal homeland: Evaluation of the ‘On track watch’ project." Evaluation and Program Planning 74: 38-53. |
| 1. Goodman, M. S., et al. (2019). "Content validation of a quantitative stakeholder engagement measure." Journal of Community Psychology 47(8): 1937-1951. |
| 1. Warner, G., et al. (2019). "‘i felt like a human being’—an exploratory, multi‐method study of refugee involvement in the development of mental health intervention research." Health Expectations: An International Journal of Public Participation in Health Care & Health Policy. |
| 1. Ken-Opurum, J., et al. (2019). "A mixed-methods evaluation using effectiveness perception surveys, social network analysis, and county-level health statistics: A pilot study of eight rural Indiana community health coalitions." Evaluation and Program Planning 77. |
| 1. Tabriz, A. A., et al. (2019). "Logic model framework for considering the inputs, processes and outcomes of a healthcare organization-research partnership." BMJ Quality & Safety: 11. |
| 1. Duran, B., et al. (2019). "Toward Health Equity: A National Study of Promising Practices in Community-Based Participatory Research." Progress in Community Health Partnerships 13(4): 337-352. |
| 1. Lessard, D., et al. (2019). "Evaluation of a project to engage patients in the development of a patient‐reported measure for HIV care (the I‐Score Study)." Health Expectations 22(2): 209-225. |
| 1. Pavarini, G., et al. (2019). "Co‐producing research with youth: The NeurOx young people's advisory group model." Health Expectations 22(4): 743-751. |
| 1. Rarere, M., et al. (2019). "Critical reflection for researcher–community partnership effectiveness: the He Pikinga Waiora process evaluation tool guiding the implementation of chronic condition interventions in Indigenous communities." Australian Journal of Primary Health 25(5): 478-485. |
| 1. Barger, S., et al. (2019). "Effective stakeholder engagement: design and implementation of a clinical trial (SWOG S1415CD) to improve cancer care." BMC Medical Research Methodology 19(1): 119. |
| 1. Abelson, J., et al. (2019). "Supporting the evaluation of public and patient engagement in health system organizations: Results from an implementation research study." Health Expectations 22(5): 1132-1143. |
| 1. Roberge-Dao, J., et al. (2019). "A mixed-methods approach to understanding partnership experiences and outcomes of projects from an integrated knowledge translation funding model in rehabilitation." BMC Health Services Research 19(1): 230. |
| 1. Crocker, J. C., et al. (2019). "Patient and public involvement (PPI) in UK surgical trials: a survey and focus groups with stakeholders to identify practices, views, and experiences." Trials [Electronic Resource] 20(1): 119. |
| 1. Hemphill, R., et al. (2019). "What motivates patients and caregivers to engage in health research and how engagement affects their lives: Qualitative survey findings." Health expectations : an international journal of public participation in health care and health policy. |
| 1. Langlois, E. V., et al. (2019). "Embedding implementation research to enhance health policy and systems: A multi-country analysis from ten settings in Latin America and the Caribbean." Health Research Policy and Systems 17 (1) (no pagination)(85). |
| 1. Faulkner, A., et al. (2019). "'Dignity and respect': An example of service user leadership and co-production in mental health research." Health expectations : an international journal of public participation in health care and health policy. |
| 1. Soobiah, C., et al. (2019). "Engaging knowledge users in a systematic review on the comparative effectiveness of geriatrician-led models of care is possible: A cross-sectional survey using the Patient Engagement Evaluation Tool." Journal of Clinical Epidemiology 113: 58-63. |
| 1. Blank, A. E., et al. (2019). "Bronx Community Collaborative Opportunities for Research and Education: Implementation and Evaluation of a Community-Academic Partnership." Progress in community health partnerships : research, education, and action 13(3): 273-282. |
| 1. Dickson, E., et al. (2020). "Characteristics and Practices Within Research Partnerships for Health and Social Equity." Nursing Research 69(1): 51-61. |
| 1. Rodríguez Espinosa, P., et al. (2020). "Personal Outcomes in Community‐based Participatory Research Partnerships: A Cross‐site Mixed Methods Study." American Journal of Community Psychology 66(3/4): 439-449. |
| 1. Coombe, C. M., et al. (2020). "A Participatory, Mixed Methods Approach to Define and Measure Partnership Synergy in Long‐standing Equity‐focused CBPR Partnerships." American Journal of Community Psychology 66(3/4): 427-438. |
| 1. Snijder, M., et al. (2020). "'We walked side by side through the whole thing': A mixed‐methods study of key elements of community‐based participatory research partnerships between rural Aboriginal communities and researchers." Australian Journal of Rural Health 28(4): 338-350. |
| 1. O'Donovan, J., et al. (2020). "Participatory approaches, local stakeholders and cultural relevance facilitate an impactful community-based project in Uganda." Health Promotion International 35(6): 1353-1368. |
| 1. Lucero, J. E., et al. (2020). "Engage for Equity: The Role of Trust and Synergy in Community-Based Participatory Research." Health Education & Behavior 47(3): 372-379. |
| 1. Spitzer-Shohat, S., et al. (2020). "Development of a novel social incubator for health promoting initiatives in a disadvantaged region." BMC Public Health 20(1): 1-9. |
| 1. Knudsen, H. K., et al. (2020). "Model and approach for assessing implementation context and fidelity in the HEALing Communities Study." Drug & Alcohol Dependence 217: N.PAG-N.PAG. |
| 1. Aguirre, M. C., et al. (2020). "The role of advocacy in adapting the diabetes prevention program for couple-based delivery that reaches marginalized groups." the Behavior Therapist 43(7): 261-265. |
| 1. Hughes, G., et al. (2020). "Evaluating patient attitudes to increased patient engagement with antimicrobial stewardship: A quantitative survey." JAC-Antimicrobial Resistance 2(3). |
| 1. Hinrichsen, C., et al. (2020). "Implementing mental health promotion initiatives-process evaluation of the abcs of mental health in denmark." International Journal of Environmental Research and Public Health 17(16): 1-26. |
| 1. Toledo-Chavarri, A., et al. (2020). "Evaluation of patient involvement strategies in health technology assessment in Spain: The viewpoint of HTA researchers." International Journal of Technology Assessment in Health Care. |
| 1. Luchtenberg, M. L., et al. (2020). "'I actually felt like i was a researcher myself.' on involving children in the analysis of qualitative paediatric research in the Netherlands." BMJ Open 10(8): 034433. |
| 1. Gafos, M., et al. (2020). ""PROUD to have been involved": an evaluation of participant and community involvement in the PROUD HIV prevention trial." Research involvement and engagement 6: 13. |
| 1. Drebit, S., et al. (2020). "Evaluation of Patient Engagement in a Clinical Emergency Care Network: Findings From the BC Emergency Medicine Network." Journal of patient experience 7(6): 937-940. |
| 1. Bhati, D. K., et al. (2020). "Patients' engagement in primary care research: a case study in a Canadian context." Research involvement and engagement 6(1): 65. |
| 1. van Schelven, F., et al. (2020). "Patient and public involvement of young people with a chronic condition: lessons learned and practical tips from a large participatory program." Research involvement and engagement 6: 59. |
| 1. Haesebaert, J., et al. (2020). ""They heard our voice!" patient engagement councils in community-based primary care practices: a participatory action research pilot study." Research involvement and engagement 6: 54. |
| 1. Vat, L. E., et al. (2020). "Giving patients a voice: a participatory evaluation of patient engagement in Newfoundland and Labrador Health Research." Research involvement and engagement 6: 39. |
| 1. Birch, R., et al. (2020). "Development and formative evaluation of patient research partner involvement in a multi-disciplinary European translational research project." Research involvement and engagement 6: 6. |
| 1. Seeralan, T., et al. (2021). "Patient involvement in developing a patient‐targeted feedback intervention after depression screening in primary care within the randomized controlled trial GET.FEEDBACK.GP." Health Expectations 24: 95-112. |
| 1. Alexander, R., et al. (2021). Capacity Development and Evaluation of a Parent Advisory Team Engaged in Childhood Obesity Research. Health Promotion Practice 22(1): 102-111. |
| 1. den Houting, J., et al. (2021). "'I'm not just a guinea pig': Academic and community perceptions of participatory autism research." Autism: The International Journal of Research & Practice 25(1): 148-163. |
| 1. Scholz, B., et al. (2021). "'People Just Need to Try It to Be Converted!': A Picture of Consumer Mental Health Research in Australia and New Zealand." Issues in Mental Health Nursing 42(3): 249-255. |
| 1. van Schelven, F., et al. (2021). "Exploring the impact of patient and public involvement with young people with a chronic condition: A multilevel analysis." Child: Care, Health and Development 47(3): 349-356. |
| 1. Hamilton, C. B., et al. (2021). "Shortening and validation of the patient engagement in research scale (peirs) for measuring meaningful patient and family caregiver engagement." Health Expectations: An International Journal of Public Participation in Health Care & Health Policy. |
| 1. Boursaw, B., et al. (2021). "Scales of practices and outcomes for community‐engaged research." American Journal of Community Psychology. |
| 1. Loban, E., et al. (2021). "Measuring partnership synergy and functioning: Multi-stakeholder collaboration in primary health care." PLoS ONE 16(5 May): e0252299. |
| 1. Martinez, J., et al. (2021). "Evaluating Stakeholder Engagement: Stakeholder-Centric Instrumentation Process (SCIP)." Western Journal of Nursing Research: 1939459211004274. |
| 1. Moore de Peralta, A., et al. (2021). "A Contribution to Measure Partnership Trust in Community-Based Participatory Research and Interventions With Latinx Communities in the United States." Health Promotion Practice: 15248399211004622. |
| 1. Livingstone, H., et al. (2021). "Evaluation of the impact of patient input in health technology assessments at NICE." International Journal of Technology Assessment in Health Care: e33. |
| 1. March, C., et al. (2021). "Engaging stakeholders: Can we measure the impact on pediatrics diabetes research?" Pediatric Diabetes 22(SUPPL 29): 72-73. (EP 103) |
| 1. Enard, K. R., et al. (2021). "In pursuit of equity: partnering to improve breast and prostate cancer outcomes among African Americans." Cancer Causes and Control 32(5): 473-482. |
| 1. Rasburn, M., et al. (2021). "Strengthening patient outcome evidence in health technology assessment: A coproduction approach." International Journal of Technology Assessment in Health Care. |
| 1. Knowles, S. E., et al. (2021). "More than a method: trusting relationships, productive tensions, and two-way learning as mechanisms of authentic co-production." Research involvement and engagement 7(1): 34. |
| 1. Nunn, J. S., et al. (2021). "Involving elderly research participants in the co-design of a future multi-generational cohort study." Research involvement and engagement 7(1): 23. |
| 1. Chung, J., et al. (2021). "Assessment of Stakeholder Engagement in a Down Syndrome Research Study." Journal of patient-centered research and reviews 8(1): 64-67. |

# **Appendix 13** PRISMA- ScR Checklist

| **SECTION** | **ITEM** | **PRISMA-ScR CHECKLIST ITEM** | **REPORTED ON PAGE #** |
| --- | --- | --- | --- |
| **TITLE** | | | |
| Title | 1 | Identify the report as a scoping review. | P1 |
| **ABSTRACT** | | | |
| Structured summary | 2 | Provide a structured summary that includes (as applicable): background, objectives, eligibility criteria, sources of evidence, charting methods, results, and conclusions that relate to the review questions and objectives. | P4-5 |
| **INTRODUCTION** | | | |
| Rationale | 3 | Describe the rationale for the review in the context of what is already known. Explain why the review questions/objectives lend themselves to a scoping review approach. | P6-8 |
| Objectives | 4 | Provide an explicit statement of the questions and objectives being addressed with reference to their key elements (e.g., population or participants, concepts, and context) or other relevant key elements used to conceptualize the review questions and/or objectives. | P8 |
| **METHODS** | | | |
| Protocol and registration | 5 | Indicate whether a review protocol exists; state if and where it can be accessed (e.g., a Web address); and if available, provide registration information, including the registration number. | P5,8 |
| Eligibility criteria | 6 | Specify characteristics of the sources of evidence used as eligibility criteria (e.g., years considered, language, and publication status), and provide a rationale. | P9-10 and Table 2 |
| Information sources* | 7 | Describe all information sources in the search (e.g., databases with dates of coverage and contact with authors to identify additional sources), as well as the date the most recent search was executed. | P9-10 |
| Search | 8 | Present the full electronic search strategy for at least 1 database, including any limits used, such that it could be repeated. | P9, Appendix 4 (ADDITIONAL) |
| Selection of sources of evidence† | 9 | State the process for selecting sources of evidence (i.e., screening and eligibility) included in the scoping review. | P10-11, Appendix 3 (ADDITIONAL) and Appendix 5 (ADDITIONAL) |
| Data charting process‡ | 10 | Describe the methods of charting data from the included sources of evidence (e.g., calibrated forms or forms that have been tested by the team before their use, and whether data charting was done independently or in duplicate) and any processes for obtaining and confirming data from investigators. | P9-10. Appendix 3 (ADDITIONAL) |
| Data items | 11 | List and define all variables for which data were sought and any assumptions and simplifications made. | P10, Appendices 2 & 3 (ADDITIONAL) |
| Critical appraisal of individual sources of evidence§ | 12 | If done, provide a rationale for conducting a critical appraisal of included sources of evidence; describe the methods used and how this information was used in any data synthesis (if appropriate). | P10, Appendix 8 (ADDITIONAL) |
| Synthesis of results | 13 | Describe the methods of handling and summarizing the data that were charted. | P10, Appendices 2 & 3 (ADDITIONAL) |
| **RESULTS** | | | |
| Selection of sources of evidence | 14 | Give numbers of sources of evidence screened, assessed for eligibility, and included in the review, with reasons for exclusions at each stage, ideally using a flow diagram. | P10-11, Figure 1 (PRISMA Flow Diagram) |
| Characteristics of sources of evidence | 15 | For each source of evidence, present characteristics for which data were charted and provide the citations. | P10-18 Tables 3-6 and Figures 1-4, and ADDITIONAL File |
| Critical appraisal within sources of evidence | 16 | If done, present data on critical appraisal of included sources of evidence (see item 12). | NA |
| Results of individual sources of evidence | 17 | For each included source of evidence, present the relevant data that were charted that relate to the review questions and objectives. | P10-18 with related figures and tables (Tables 3-6, Figures 1-4) and ADDITIONAL File |
| Synthesis of results | 18 | Summarize and/or present the charting results as they relate to the review questions and objectives. | P10-18 with related figures and tables (Tables 3-6, Figures 1-4) and ADDITIONAL File |
| **DISCUSSION** | | | |
| Summary of evidence | 19 | Summarize the main results (including an overview of concepts, themes, and types of evidence available), link to the review questions and objectives, and consider the relevance to key groups. | P18, Table 6 |
| Limitations | 20 | Discuss the limitations of the scoping review process. | P22-23 |
| Conclusions | 21 | Provide a general interpretation of the results with respect to the review questions and objectives, as well as potential implications and/or next steps. | P24 |
| **FUNDING** | | | |
| Funding | 22 | Describe sources of funding for the included sources of evidence, as well as sources of funding for the scoping review. Describe the role of the funders of the scoping review. | P25 |

*From:* Tricco AC, Lillie E, Zarin W, O'Brien KK, Colquhoun H, Levac D, et al. PRISMA Extension for Scoping Reviews (PRISMAScR): Checklist and Explanation. Ann Intern Med. 2018;169:467–473. [doi: 10.7326/M18-0850](http://annals.org/aim/fullarticle/2700389/prisma-extension-scoping-reviews-prisma-scr-checklist-explanation).

# **References**

1. Sampson, M., McGowan, J., Cogo, E., Grimshaw, J., Moher, D., Lefebvre, C., *An evidence-based practice guideline for the peer review of electronic search straetgies.* Journal of Clinical Epidemiology, 2009. **62**: p. 944-952.

2. McGowan, J., Sampson, M., Salzwedel, D., Cogo, E., Foerster, V., Lefebvre, C., *Guideline Statement: PRESS Peer Review of Electronic Search Strategies 2015 Guideline Statement.* Journal of Clinical Epidemiology, 2016. **75**: p. 40-46.

3. Porter, L., *Planning in (post) colonial settings: Challenges for theory and practice.* Planning Theory and Practice, 2006. **7**(4): p. 383-396.

4. Byrne, M. *Five terms to use as an alternative to “third world”.* 2019 3 August, 2019 [cited 2022 19 January]; Available from: <https://borgenproject.org/tag/majority-world/>.

5. Hoekstra, F., Mrklas, K.J.*, Sibley, K., Nguyen, T., Vis-Dunbar, M., Neilson, C.J., Crockett, L.K., Gainsforth, H.L.^, Graham, I.D^. (*co-authors, ^co-senior authors), *A Review Protocol on Research Partnerships: A Coordinated Multicenter Team Approach.* Systematic Reviews, 2018. **7**(217): p. 1-14.

6. University of Waterloo. *Research Ethics: Definition of a health outcome.* 2018 [cited 2018 March 7]; Available from: <https://uwaterloo.ca/research/office-research-ethics/research-human-participants/pre-submission-and-training/human-research-guidelines-and-policies-alphabetical-list/definition-health-outcome>.

7. Higher Education Funding Council for England: Research Excellence Framework 2014. *Assessment framework and guidance on submissions 2011.* 2014 [cited 14 Nov 2017; Available from: <http://www.ref.ac.uk/2014/media/ref/content/pub/assessmentframeworkandguidanceonsubmissions/GOS%20including%20addendum.pdf>.

8. Stanick, C.F., Halko, H.M., Nolen, E.A., Powell, B.J., Dorsey, C.N., Mettert, K.D, Weiner, B.J., Barwick, M., WOlfenden, L., Damschroder, L.J., Lewis, C.C., *Pragmatic measures for implementation research: development of the Psychometric and Pragmatic Evidence Rating Scale (PAPERS).* Translational Behavioural Medicine, 2021. **11**(1): p. 11-20.

9. Lewis, C.C., Mettert, K.D., Stanick, C.F., Halko, H.M., Nolen, E.A., Powell, B.J., Weiner, B.J., *The psychometric and pragmatic evidence rating scale (PAPERS) for measure development and evaluation.* Implementation Research and Practice, 2021(January).

10. Graham, I.D., Beardall, S., Carter, A.O., Glennie, J., Hebert, P.C., Tetroe, J.M., McAlister, F.A>, Visentin, S., Anderson, G.M., *What is the quality of drug therapy clinical practice guidelines in Canada?* Canadian Medical Association Journal, 2001. **165**(2): p. 157-163.

11. Centre of Excellence on Partnership with Patients and the Public (CEPPP). *Patient and Public Engagement Evaluation Toolkit*. 2021 [cited 2021 23 Nov]; Available from: <https://ceppp.ca/en/evaluation-toolkit/#care>||div1|category_evaluation-toolkit-project|1.

12. Boivin, A., L’Esperance, A., Gauvin, F.P., Dumez, V., Maccaulay, A.C., Lehoux, P., Abelson, J., *Patient and public engagement in research and health system decision making: A systematic review of evaluation tools.* Health Expectations, 2018. **21**(6): p. 1075-1084.

13. IKTRN (Integrated Knowledge Translation Research Network). *Resources: Our Publications*. 2021 [cited 2021 23 November]; Available from: <https://iktrn.ohri.ca/resources/publications/>.

14. Hoekstra, F., Mrklas, K.J., Khan, M., McKay, R.C., Vis-Dunbar, M., Sibley, K., Nguyen, T., Graham, I.D., SCI Guiding Principles Consensus Panel, & Gainforth, H.L., *A review of reviews on principles, strategies, outcomes and impacts of research partnerships approaches: a first step in synthesising the research partnership literature.* Health Research Policy and Systems, 2020. **18**(51).

15. Hoekstra, F., Trigo, F., Sibley, K., Graham, I.D., Kennefick, M., Mrklas, K.J., Nguyen, T., Vis-Dunbar, M., Gainforth, H.L.,, *Systematic overviews of partnership principles and strategies identified from health research about spinal cord injury and related health conditions: a scoping review.* The Journal of Spinal Cord Medicine, 2021. **TBD**(TBD).

16. Kothari, A., McCutcheon, C., Graham, I.D., for the iKT Research Network., *Defining Integrated Knowledge Translation and Moving Forward: A Reponse to Recent Commentaries.* International Journal of Health Policy and Management, 2017. **6**: p. 1-2.

17. Graham, I.D., et al., *Moving knowledge into action for more effective practice, programmes and policy: protocol for a research programme on integrated knowledge translation.* Implementation Science, 2018. **13**(1): p. 22.

18. Tigges, B.B., Miller, D., Dudding, K.M., Balls-Berry, J.E., et al.,, *Measuring quality and outcomes of research collaborations: An integrative review.* Journal of Clinical and Translational Science, 2019. **3**: p. 261-289.

19. Daigneault, P.M., *Taking stock of four decades of quantitative reserach on stakeholder participation and evaluation use: A systematic map.* Evaluation and Program Planning, 2014. **45**: p. 171-181.

20. MacGregor, S., *An overview of quantitative instruments and measures for impact in co-production.* Journal of Professional Capital and Community, 2020. **6**(2): p. 163-183.

21. Sandoval, J.A., Lucero J., Oetzel, J., Avila, M., Belone, L., Mau, M., Pearson, C., Tafoya, G., Duran, B., Iglesias Rios, L., Wallerstein, N., *Process and outcome constructs for evaluating community-based participatory research projects: a matrix of existing measures.* Health Education Research, 2012. **27**(4): p. 680-690.

22. Arksey, H., & O'Malley, L., *Scoping studies: towards a methodological framework.* International Journal of Social Research Methodology: Theory and Practice, 2005. **8**(1): p. 19-32.

23. Levac, D., Colquhoun, H., & O'Brien, K.K., *Scoping studies: advancing the methodology.* Implementation Science, 2010. **5**(69): p. 1-9.

24. Daudt, H.M., van Mossel, C., Scott, S.J., *Enhancing the scoping study methodology: a large, inter-professional team's experience with Arksey and O'Malley's framework.* BMC Medical Research Methodology, 2013. **13**(48): p. 1-9.

25. Colquhoun, H.I., Levac, D., O'Brien, K.K., Straus, S., Tricco, A.C., Perrier, L., Kastner, M., & Moher, D., *Scoping Reviews: Time for clarity in defintion, methods and reporting.* Journal of Clinical Epidemiology, 2014. **67**(12): p. 1291-1294.

26. Tricco, A.C., Lillie, E., Zarin, W., O'Brien, K., Colquhoun, H., Kastner, M., Levac, D., Ng, C., Pearson Sharpe, J., Wilson, K., Kenny, M., Warren, R., Wilson, C., Stelfox, H.T., & Straus, S.E., *A scoping review on the conduct and reporting of scoping reviews.* BMC Medical Research Methodology, 2016. **16**(15): p. 1-10.

27. Tricco, A.C., Lillie, E., Zarin, W., O'Brien, K., Colquhoun, H., Levac, D., Moher, D., Peters, M.D.J., Horsley, T., Weeks, L., Hempel, S., Akl, E.A., Chang, C., McGowan, J., Steward, L., Hartling, L., Aldcroft, Al., Wilson, M.G., Garrity, C., Lewin, S., Godfrey, C.M., MacDonald, M.T., Langlois, E.V., Soares-Weiser, K. Moriarty, J., Clifford, T., Tuncalp, O., Straus, S.E., *PRISMA Extension for Scoping Reviews (PRISMA-ScR): Checklist and Explanation.* Annals of Internal Medicine, 2018. **169**(7): p. 467-473.

28. Higgins, J., Thomas, J., Chandler, J., Cumpston, M., Li, T., Page, M.J., Welch, V.A. (Eds). *Cochrane Handbook for Systematic Reviews of Interventions, Version 6.2*. 2021, Cochrane.

29. Page, M.J., McKenzie, J.E., Bossuyt, P.M., Boutron, I., Hoffmann, T.C., Mulrow, C.D., et al., *The PRISMA 2020 statement: an updated guideline for reporting systematic reviews.* BMJ, 2021. **372**.

30. Centre for Reviews and Dissemination (CRD), U.o.Y., . *Systematic Reviews: CRD's Guidance for Undertaking Reviews in Health Care*. 2009, CRD, University of York,. Layerthorpe, York, UK.

31. Joanna Briggs Institute, *Joanna Briggs Institute Reviewers' Manual: 2015 edition/Additional*. 2015, The Joanna Briggs Institute: South Australia, AU.

32. Bidwell, S., Jensen, M.F.,. *Etext on Health Technology Assessment (HTA) Information Resources. - Chapter 3: Using a search protocol to identify sources of information: the COSI model.* 2000 [cited 2019 July 2]; Available from: <http://www.nlm.nih.gov/archive/20060905/nichsr/ehta/hta.html>.

33. Sampson, M., *Should we change how we do our searches? Objectively Derived Search Strategies or 'Exhaustive Search method' as performed by Bramer.* 2016, Childrens Hospital of Eastern Ontario (CHEO) Research Institute, University of Ottawa: Ottawa. p. 1-34.

34. Mrklas, K.J., et al.,. *Open Science Framework File: Ovid MEDLINE Search Strategy (Scoping review of the globally avaialble tools to assess the outcomes and impacts of health research partnerships).* 2018 31 October 2018 [cited 2021 23 November]; Available from: <https://osf.io/4aehv/?view_only=dbac88b1d0324305b131186040dba863>.

35. Bramer, W.M., Giustini, D., de Jonge, G.B., Holland, L., Bekhuis, T., *De-duplication of database search results for systematic reviews in Endnote.* Journal of the Medical Library Association (JMLA), 2016. **104**(3): p. 240-243.

36. Microsoft Corporation., *Microsoft Excel for Mac 2021*, V. (21101001), Editor. 2021, 2021 Microsoft Corporation.

37. Armstrong, R., Hall, B.J., Doyle, J., Waters, E.,, *‘Scoping the scope’ of a cochrane review.* J Public Health, 2011. **33**(1): p. 147-150.

38. Valaitis, R., Martin-Misenter, R., Wong, S.T., et al., , *Methods, strategies and technologies used to conduct a scoping literature review of collaboration between proimary care and public health.* Prim Health Care Res Dev, 2012. **13**(3): p. 219-36.

39. Altman, D.G., *Practical Statistics for Medical Research: Measuring Agreement*. 1991, London, UK: Chapman and Hall.

40. Polanin, J.R., Pigott, T.D., Espelage, D.L., Grotpeter, J.K., *Best practice guidelines for abstract screening large-evidence systematic reviews and meta-analyses.* Research Synthesis Methods, 2019. **10**(3): p. 330-342.

41. O’Blenis, P., *Data Extraction: Weighing your options.*, in *Evidence Partners*. 2016, Evidence Partners Inc.

42. Terwee, C.B., de Vet, H.C.W., Prinsen, C.A.C., Mokkink, L.B. *Protocol for Systematic Reviews of Measurement Properties*. 2011.

43. Scherer, R.W., Saldanha, I.J., *How should systematic reviewers handle conference abstracts? A view from the trenches.* Systematic Reviews, 2019. **8**(264).

44. McHugh, M.L., *Interrater reliability: the kappa statistic.* Biochemia Medica, 2012. **22**(3): p. 276-282.

45. Mrklas, K.J., et al.,. *Open Science Framework File: Towards the development of a valid, reliable and acceptable tool for assessing the impact of health research partnerships (Protocols).* 2021 19 April 2021 23 November 2021]; Available from: <https://mfr.ca-1.osf.io/render?url=https://osf.io/j7cxd/?direct%26mode=render%26action=download%26mode=render>.

46. Mokkink, L.B., Terwee, C.B., Patrick, D.L., Alonso, J., Stratford, P.W., Knol, D.L., Bouter, L.M., de Vet, H.C.W., *COSMIN Checklist Manual*. 2012.

47. American Educational Research Association., A.P.A., and National Council on Measurement in Education (Eds). *Standards for educational and psychological testing.* 2014, American Educational Research Association: Washington DC.

48. Bowen, D.J., Hyams, T., Goodman, M., West, K.M., Harris-Wai, J., Yu, J.H.,, *Systematic review of quantitative measures of stakeholder engagement.* Cilin Transl Sci, 2017. **10**: p. 314-336.

49. Dixon, N., Pearce, M., *Guide to ensuring data quality in clinical audits.* 2011, Healthcare Quality Improvement Partnership: United Kingdom. p. 50pp.

50. National Institute for Clinical Excellence, *Principles for best practice in clinical audit.* 2002: Oxon, UK. p. 206pp.

51. Research and Evaluation Unit., W.R.H.A. *Random sample calculator.* 2014 [cited 2021 03 Nov]; Available from: <https://www.wrha.mb.ca/extranet/eipt/files/Sampcalcaudits.xls>.

52. Heyland, D.K., Rocker, G.M., Dodek, P.M., Kutsogiannis, D.J., Konopad, E., Cook, D.J., Peters, S., Tranmer, J.E., O'Callaghan, C.J., *Family satisfaction with care in the intensive care unit: results of a multiple centre study.* Critical Care Medicine, 2002. **30**: p. 1413-1418.

53. Joanna Briggs Institute, *The Joanna Briggs Institute Reviewers’ Manual 2015*. 2015, Joanna Briggs Institute: South Australia. p. 24pp.
