# Supplementary material for: A scoping review of the globally available tools for assessing health research partnership outcomes and impacts
Source: Health Res Policy Syst. 2023 Dec 22;21:139. doi: 10.1186/s12961-023-00958-y (PMC10740226; doi:10.1186/s12961-023-00958-y)
Supplement: Supplementary file 2 — Additional file 2: Table S1. Characteristics of included studies, Table S2. Tool characteristics [file 12961_2023_958_MOESM2_ESM.docx]

# **Table S1** Characteristics of included studies

(n=166 studies with 3 companion reports)

**Notes** 1 companion report 2 Latin America: Argentina, Bolivia, Brazil, Chile, Colombia, Mexico, Peru; Caribbean: Saint Lucia

a CBPR Community-based participatory research; CEnR Community Engagement Research; PBRN Practice Based Research Network; PAR Participatory Action Research; IKT Integrated Knowledge Translation; PPI patient and public involvement; PPE Patient or public engagement; PAG Patient advocacy or advisory group; PCOR Patient Centred Outcomes Research

b Met (Methods) Qu Qualitative study, Qn quantitative study, MM mixed methods study

c Study activities D (developed tool), M (modified tool), U (used tool), E (evaluated tool), V (validated tool) to assess health research partnership outcomes, impacts.

d Validated (any type, conceptual/theoretical and/or empirical psychometric validation)

| **First author, Year** | **Country** | **Health**  **Sub-domain** | **Focus^a^** | **Study Design** | **Method^b^** | **Instrumentation** | **Scope of Study Activity^c^** | | | | |
| --- | --- | --- | --- | --- | --- | --- | --- | --- | --- | --- | --- |
|  |  |  |  |  |  |  | **D** | **M** | **U** | **E** | **V^d^** |
| Butterfoss, 1996 | USA | Substance Use Prevention | coalition evaluation | mixed methods | MM | cross-sectional survey, modelling | ✓ | ✓ | ✓ |  | ✓ |
| Goodman, 1996 | USA | Substance Use Prevention | coalition evaluation | mixed methods | MM | pre-post survey, needs assessment checklist, plan quality index, telephone surveys, policy case study |  |  | ✓ |  |  |
| Goldstein, 1997 | USA | Health and Wellness Promotion | coalition evaluation | mixed methods | MM | consultation, pilot survey, feedback | ✓ |  | ✓ |  |  |
| Kegler, 1998 | USA | Cancer Prevention | coalition evaluation | cross-sectional study | Qn | Survey |  | ✓ | ✓ |  | ✓ |
| Armbruster, 1999 | USA | Health Promotion | elder care | cross-sectional study | MM | Survey |  | ✓ | ✓ |  |  |
| Chan, 2000 | USA | Community Development | partnership evaluation | mixed methods | MM | survey, interviews, document review |  | ✓ | ✓ |  | ✓ |
| Lantz, 2001 | USA | Public health, disease prevention | coalition evaluation | mixed methods | MM | longitudinal cross-sectional survey, qualitative interviews |  |  | ✓ |  |  |
| Shortell, 2002 | USA | Community Health Improvement | partnership assessment | mixed methods | MM | survey, semi-structured telephone interviews, focus groups, document analysis | ✓ |  | ✓ |  | ✓ |
| Weiss, 2002 | USA | Health Services Research | partnership evaluation and effectiveness | cross-sectional study | Qn | Survey | ✓ |  | ✓ |  | ✓ |
| Schulz, 2003 | USA | CBPR | partnership evaluation | multiple case study | MM | longitudinal survey, field notes, interviews, process questionnaire, focus groups, semi-structured interviews, document review |  | ✓ | ✓ |  |  |
| Cotter, 2003 | USA | Alzheimer's Disease | research partnership evaluation | mixed methods | MM | monthly semi-structured logs | ✓ |  | ✓ |  |  |
| Butterfoss, 2004 | USA | Perinatal health | community coalition evaluation | mixed methods | MM | Coalition Needs Assessment (CNA), Meeting Effectiveness Inventory (MEI), Coalition Effectiveness Inventory (CEI), semi-structured interviews |  |  | ✓ |  |  |
| El Ansari, 2004 | UK, South Africa | Community Development | partnership assessment | cross-sectional study | Qn | Survey |  | ✓ | ✓ |  | ✓ |
| El Ansari, 2004 | UK, South Africa | Community Development | partnership assessment | cross-sectional study | Qn | Survey |  | ✓ | ✓ |  |  |
| Metzger, 2005 | USA | Community Health | coalition evaluation | cross-sectional study | Qn | Survey | ✓ | ✓ | ✓ | ✓ | ✓ |
| Kramer, 2005 | USA | Adolescent pregnancy prevention | coalition evaluation | mixed methods | MM | longitudinal survey, qualitative interviews, and post-site visit survey | ✓ | ✓ | ✓ |  |  |
| Kegler, 2005 | USA | Adolescent pregnancy prevention | coalition evaluation | cross-sectional study | Qn | Survey |  | ✓ | ✓ |  | ✓ |
| Cramer, 2006 | USA | Substance Use Prevention | community coalition evaluation | cross-sectional study | Qn | Survey | ✓ |  | ✓ |  | ✓ |
| Savitz, 2007 | USA | Health Research Quality | partnership research evaluation | cross-sectional study | MM | Survey |  | ✓ | ✓ |  |  |
| Barber, 2007 | UK | Health  Research | partnership research evaluation | cross-sectional study | MM | Survey | ✓ |  | ✓ |  |  |
| Feinberg, 2008 | USA | Public Health and Prevention | community coalition assessment | cross-sectional study | Qn | longitudinal survey |  |  | ✓ |  | ✓ |
| Feinberg, 2008b | USA | Public Health and Prevention | community coalition assessment | cross-sectional study | Qn | longitudinal survey |  |  |  |  | ✓ |
| Wyatt, 2008 | UK | Primary Care | consumer engagement evaluation | mixed methods | MM | survey, interviews, observation, document review | ✓ |  | ✓ |  |  |
| Lovell, 2008 | Canada | Health Geography | CBPR assessment | multiple case study | MM | interviews, focus groups, surveys, observation |  | ✓ | ✓ |  |  |
| Garner, 2008 | USA | Substance Use Prevention | coalition evaluation | case study | MM | interviews, focus groups, surveys, document analysis, coalition tracking, participant observation |  | ✓ | ✓ |  |  |
| Orr Brawer, 2008 | USA | Community Health | coalition evaluation | mixed methods | MM | surveys, interviews, focus groups | ✓ | ✓ | ✓ |  | ✓ |
| Adily, 2009 | Australia | Knowledge Transfer & Exchange | research partnership evaluation | cross-sectional study | Qn | Survey | ✓ |  |  | ✓ |  |
| King, 2009 | Canada | Health & Social Services | research partnership impact evaluation | mixed methods | MM | focus groups, survey | ✓ |  |  | ✓ | ✓ |
| Van Olphen, 2009 | USA | Cancer Prevention | CBPR evaluation | mixed methods | MM | survey, interviews, focus group |  | ✓ | ✓ |  |  |
| Sunderland, 2009 | Australia | Chronic Disease Prevention and Management | partnership evaluation | mixed methods | MM | semi-structured interviews, partnership tool survey |  | ✓ | ✓ | ✓ |  |
| Tolma, 2009 | USA | Public Health | partnership evaluation | mixed methods | MM | surveys, checklists, interviews, observations | ✓ | ✓ | ✓ |  |  |
| Barnidge, 2010 | USA | Chronic Disease Prevention and Control | partnership evaluation | descriptive study | MM | framework development, literature review, interviews, focus groups, self-assessment checklists | ✓ |  | ✓ |  |  |
| Blevins, 2010 | USA | Mental Health and Addictions | partnership research evaluation | mixed methods | MM | survey, focus groups, archival data, interviews |  | ✓ | ✓ |  |  |
| Wagemakers, 2010 | Netherlands | Community Health Promotion | partnership evaluation | multiple case study | MM | checklist | ✓ |  |  | ✓ | ✓ |
| King, 2010 | Canada | Health and Social Services Research | partnership evaluation | cross-sectional study | Qn | checklist, impact questionnaire |  |  | ✓ |  |  |
| Wright, 2010 | USA | Cancer  Research | PPI impact, quality evaluation | descriptive study | Qu | nominal group technique, prioritization, guidelines for appraising user involvement | ✓ |  |  |  |  |
| Ziff, 2010 | USA | HIV and AIDS Prevention Research | coalition evaluation | cross-sectional study | Qn | longitudinal survey |  |  | ✓ |  | ✓ |
| Raine, 2010 | Canada | Obesity and Chronic Disease Prevention | partnership assessment | mixed methods | MM | survey tool |  | ✓ | ✓ |  |  |
| Jones, 2011 | Ireland | Health Promotion | partnership evaluation | mixed methods | MM | focus groups, survey | ✓ |  |  | ✓ | ✓ |
| Jones, 2011b | Ireland | Health Promotion | partnership evaluation | mixed methods | MM | focus groups, survey | ✓ |  | ✓ |  | ✓ |
| Payne, 2011 | Australia | Harm Reduction | community participation evaluation | cross-sectional study | MM | questionnaire | ✓ |  | ✓ |  |  |
| Perkins, 2011 | USA | Harm reduction, mental health | team function evaluation | nested longitudinal study | MM | web-based survey, semi-structured survey interviews, team dynamics ratings |  |  | ✓ |  | ✓ |
| Sanchez, 2011 | USA | Public Health and Health Promotion | community health improvement | descriptive study | MM | survey, model development, document review, co-design meetings | ✓ | ✓ | ✓ | ✓ |  |
| VanDevanter, 2011 | USA | Hepatitis B, Equity | CBPR evaluation | mixed methods | MM | survey, key informant interviews |  | ✓ | ✓ |  |  |
| Bilodeau, 2011; Bilodeau, 2019^1^ | Canada | Public Health | Partnership assessment and tool development | mixed methods | MM | cognitive interviews, questionnaire development, group interviews; expert committee and focus group pre-test | ✓ |  |  | ✓ | ✓ |
| Allen, 2011 | USA | Immigrant Health and Wellness | CBPR partnership assessment | mixed methods | MM | Interviews, discussion notes, facilitation meetings, survey comments, longitudinal survey |  |  | ✓ |  |  |
| Curro, 2012 | USA | Dentistry | PBRN evaluation | cross-sectional study | Qn | survey | ✓ |  | ✓ |  | ✓ |
| El Ansari, 2012 | UK, South Africa | Community Partnerships | partnership assessment | mixed methods | MM | survey, semi-structured interviews | ✓ | ✓ |  |  | ✓ |
| CIHR, 2013;  McLean, 2012^1^ | Canada | Knowledge Translation Research Evaluation | health research fund program performance | mixed methods | MM | survey, interviews, document review, scan of health research funding agencies, case studies, expert panel discussion | ✓ |  | ✓ |  |  |
| Vale, 2012 | UK | Clinical Trials (HIV, cancer, infectious disease) | PPI evaluation | cross-sectional study | MM | survey | ✓ |  | ✓ |  |  |
| Martinez, 2012 | USA | Occupational Health and Safety | partnership research evaluation | mixed methods | MM | longitudinal surveys, interviews |  | ✓ | ✓ |  |  |
| Woodland, 2012 | USA | Health and Social Services | collaboration evaluation | descriptive study | MM | measures, tools, and framework (LOIR, TCAR, CEIF) | ✓ |  |  |  |  |
| Tataw, 2012 | USA | Community Health Planning | stakeholder participation evaluation | multiple case study | MM | assessment tool and framework, participant observation, review of experiences and program reports | ✓ |  | ✓ |  |  |
| Kagan, 2012 | USA | HIV/AIDS | community-researcher engagement evaluation | cross-sectional study | Qn | Survey | ✓ |  | ✓ |  |  |
| Brown, 2012 | USA | Substance Use and Violence Prevention | coalition evaluation | cross-sectional study | Qn | Survey | ✓ |  |  |  | ✓ |
| Braun, 2012 | USA | Cancer Disparities Research | academic-community partnerships evaluation | cross-sectional study | Qn | Survey | ✓ |  | ✓ |  |  |
| Stedman-Smith, 2012 | USA | Environmental and Public Health | partnership evaluation | mixed methods | MM | survey, interviews | ✓ |  | ✓ |  |  |
| Nargiso, 2013 | USA | Substance Use Prevention | coalition evaluation | cross-sectional study | Qn | Scale, rubrics | ✓ |  | ✓ |  | ✓ |
| Watson-Thompson, 2013 | USA | Substance Use | coalition capacity-building evaluation | pre-post study | Qn | community toolbox survey | ✓ |  | ✓ |  |  |
| Khodyakov, 2013 | USA | Mental Health and Addictions | partnership evaluation | mixed methods | MM | interviews, survey, and index | ✓ |  | ✓ |  |  |
| Patterson, 2014 | UK | Mental Health | service user involvement evaluation | cross-sectional study | MM | Survey | ✓ |  | ✓ |  |  |
| Rosella, 2018; Rosella, 2014^1^ | Canada | Chronic Disease Management | diabetes care | multiple case study | MM | observer notes, surveys, interviews | ✓ |  | ✓ |  |  |
| Perkins, 2014 | USA | Nursing academic-practice partnerships | partnership assessment | cross-sectional study | Qn | Survey |  | ✓ | ✓ | ✓ | ✓ |
| Chang, 2014 | Taiwan | School Health Promotion | implementation, efficacy, impact assessment | post-test study | Qn | Survey | ✓ |  | ✓ |  | ✓ |
| Arroyo-Johnson, 2015 | USA | Disparities in Cancer | CBPR outcomes assessment | cross-sectional study | MM | case study | ✓ | ✓ | ✓ |  |  |
| Brown, 2015 | USA | Substance Use and Violence Prevention | coalition evaluation | cross-sectional study | Qn | longitudinal self-report and observation-report surveys |  |  | ✓ | ✓ | ✓ |
| Gamble, 2015 | UK | Clinical Trials Research | PPI evaluation | mixed methods | MM | structured telephone interviews, surveys | ✓ |  | ✓ |  |  |
| Murphy, 2015 | Canada | Global Health Research Partnerships | Ethics | descriptive study | Qu | facilitative discussion, consultations, pre-engagement, toolkit questions | ✓ |  | ✓ |  |  |
| Soper, 2015 | UK | Health Services Research | partnership evaluation | multiple case study | MM | mapping exercise, document analysis, workshops, interviews (in-depth case study, validation), literature review, stakeholder survey | ✓ |  | ✓ |  |  |
| Truiett-Theodorson, 2015 | USA | Public Health and Disease Prevention | coalition evaluation | mixed methods | MM | focus groups, survey |  |  | ✓ |  |  |
| Wilson, 2015 | UK | Public Health | PPI evaluation | multiple case study | MM | scoping exercise, survey, interviews, document review | ✓ |  | ✓ |  |  |
| Bornstein, 2015 | USA | Public Health and Disease Prevention | coalition function | cross-sectional study | Qn | Survey |  |  | ✓ |  |  |
| Oetzel, 2015 | USA | CEnR | partnership assessment | nested cross-sectional study | Qn | Surveys | ✓ | ✓ | ✓ |  | ✓ |
| Oetzel, 2015b | USA | Health Equity | CBPR evaluation | cross-sectional study | Qn | survey, conceptual model validation |  |  |  |  | ✓ |
| Arora, 2015 | USA | CBPR | partnership assessment tool development | descriptive study | MM | brainstorming, item sorting, tool pre-testing, cognitive and qualitative interviews | ✓ |  |  |  |  |
| Stocks, 2015 | UK | Patient and Public Involvement | PPI evaluation | pre-post study | Qn | survey, tool validation |  | ✓ | ✓ |  | ✓ |
| Brown, 2016 | Mexico, USA | Substance Use and Violence Prevention | coalition evaluation | cross-sectional study | Qn | quantitative survey |  |  | ✓ |  | ✓ |
| Gibbons, 2016 | USA | CBPR | partnership evaluation | mixed methods | MM | interviews, focus groups, survey evaluation | ✓ |  | ✓ |  |  |
| Larkan, 2016 | Ireland | Global Health Research | partnership guidelines | mixed methods | MM | open-ended questionnaire, discussion notes, and facilitated discussion of framework development | ✓ |  | ✓ |  |  |
| Merkel, 2015 | USA | Rare Diseases | PAG evaluation | cross-sectional study | Qn | Survey | ✓ |  | ✓ |  |  |
| Puyalto, 2016 | Spain | Education | disabilities studies | mixed methods | MM | questionnaire, focus groups | ✓ |  | ✓ |  |  |
| Robbins, 2015 | USA | Health Services Research | primary care redesign, research co-production | cross-sectional study | Qu | Survey | ✓ |  | ✓ |  |  |
| Finch, 2016 | Australia | Sport Injury Prevention | partnership evaluation | cross-sectional study | Qn | Survey |  |  | ✓ |  |  |
| Goold, 2016 | USA | Health Disparities | partnership assessment | mixed methods | MM | longitudinal survey, semi-structured focus groups, document review, direct observations |  | ✓ | ✓ |  |  |
| Abelson, 2016 | Canada | PPE in Health Organizations | PPE evaluation | mixed methods | MM | literature review, unstructured exchanges, Delphi process, tool development, usability questionnaire | ✓ |  |  | ✓ |  |
| Dugan, 2016 | USA | Occupational Health and Safety | PAR evaluation | mixed methods | MM | rating sheet, scorecard, meeting minutes, team, and study metrics |  |  | ✓ |  |  |
| Brutt, 2017 | Germany | Evidence Synthesis in Psychology | PPI evaluation | mixed methods | MM | focus groups, survey |  | ✓ | ✓ |  |  |
| Goodman, 2017 | USA | CEnR | cancer disparities | cross-sectional study | Qn | survey | ✓ |  |  | ✓ | ✓ |
| Littlecott, 2017 | UK | Health Promotion | partnership evaluation | mixed methods | MM | survey, semi-structured interviews | ✓ |  | ✓ |  |  |
| Scarinci, 2017 | USA | Minority Health and Health Disparities Research | partnership, implementation, participatory evaluation assessment | mixed methods | MM | study documents, activity logs, partnership engagement survey, utilization/satisfaction questionnaire | ✓ |  |  | ✓ |  |
| Okazaki, 2017 | USA | Immigrant Community Health | partnership evaluation | case study | MM | survey, observations, document review, interviews | ✓ |  | ✓ |  | ✓ |
| Ray, 2017 | USA | Pediatrics | partnership evaluation | mixed methods | MM | logbook analysis, survey | ✓ |  | ✓ | ✓ |  |
| Weeks, 2017 | Canada, UK | Health Technology Assessment | PPI evaluation | cross-sectional study | MM | survey | ✓ |  | ✓ |  |  |
| Jose, 2017 | Australia | Health Promotion in Occupational Settings | partnership evaluation | case study | MM | document review, survey, interviews | ✓ |  | ✓ |  |  |
| Carroll, 2017 | Canada | Cardiovascular Research | patient engagement evaluation | mixed methods | MM | semi-structured interviews, survey |  | ✓ | ✓ |  |  |
| Blackburn, 2018 | UK | Primary Care Research | PPI evaluation | mixed methods | MM | survey, document review, review workshop | ✓ |  | ✓ |  |  |
| Burrows, 2018 | UK | Digital health | PPI evaluation | cross-sectional study | MM | two surveys | ✓ |  | ✓ |  |  |
| Forsythe, 2018 | USA | Patient Centred Outcomes Research | partnership evaluation | cross-sectional study | MM | longitudinal survey |  |  | ✓ |  |  |
| Jones, 2018 | Ireland | Health Promotion | partnership evaluation | cross-sectional study | Qn | survey | ✓ |  |  | ✓ | ✓ |
| Kazmerski, 2018 | USA | Pediatric cystic fibrosis, sexual and reproductive health | adolescent and youth stakeholder engagement | mixed methods | Qu | stakeholder process outcomes/recommendations, survey |  |  | ✓ |  |  |
| Korn, 2018 | USA | Pediatric Obesity Prevention | coalition evaluation | mixed methods | MM | systematic review, survey, interviews | ✓ |  |  | ✓ |  |
| Mann, 2018 | UK | Primary Care Chronic Disease Research | PPI evaluation | nested cross-sectional study | Qu | survey within a randomized controlled trial | ✓ |  | ✓ |  |  |
| McIsaac, 2018 | Canada | Population Health Intervention Research | IKT evaluation | case study | MM | interviews, document review, survey | ✓ |  | ✓ |  |  |
| West, 2018 | USA | Genomic Research | partnership evaluation | mixed methods | MM | interviews, scale development, cognitive interviews, survey pilot test, discourse analysis | ✓ | ✓ | ✓ |  | ✓ |
| Lobo, 2018 | Australia | Sexual Health, STBBI | partnership evaluation | case study | Qu | survey | ✓ |  | ✓ |  |  |
| Mathie, 2018 | UK | Patient and Public Involvement | evaluation of reciprocality, feedback | mixed methods | MM | questionnaire, interviews | ✓ |  | ✓ |  |  |
| Oetzel, 2018 | USA | Health Equity | CBPR evaluation | multiple case study | MM | longitudinal and brief survey, document review; on-site visits, interviews, focus groups, meeting observations, historical timelines |  | ✓ |  | ✓ | ✓ |
| Nowell, 2018 | USA | Arthritis Research | patient engagement evaluation | pre-post study | MM | survey, interviews | ✓ |  | ✓ |  |  |
| Kendall, 2018 | Canada | Chronic disease prevention | partnership evaluation | cross-sectional study | MM | survey |  | ✓ | ✓ |  |  |
| Walton, 2018 | Canada | Obesity Prevention | participant engagement evaluation | nested mixed methods study | MM | survey, focus groups within a cohort study | ✓ |  | ✓ |  |  |
| Hamilton, 2018 | Canada | Health Research | patient engagement evaluation | mixed methods | MM | interviews, literature review, e-Delphi, cognitive interviews | ✓ |  |  |  | ✓ |
| Haynes, 2019 | Australia | Rheumatic Heart Disease, Australian Aboriginals | CBPR evaluation | mixed methods | MM | focus groups, interviews, observation, emoticon survey | ✓ |  | ✓ |  |  |
| Goodman, 2019 | USA | Stakeholder engagement | tool development | delphi study | Qn | Delphi survey rounds | ✓ |  |  |  | ✓ |
| Warner, 2019 | Sweden | Refugee Mental Health | PPI evaluation | mixed methods | MM | observation, focus group, questionnaire | ✓ |  | ✓ |  |  |
| Ken-Opurum, 2019 | USA | Community Health | coalition evaluation | mixed methods | MM | survey, social network analysis, population-level statistics |  | ✓ | ✓ |  |  |
| Tabriz, 2019 | USA | Colon Cancer Research | partnership evaluation | case study | MM | document review, reflections, semi-structured questionnaire, framework analysis (inputs, processes, outcomes of partnership) | ✓ |  | ✓ |  |  |
| Duran, 2019 | USA | CEnR | partnership evaluation | mixed methods | MM | surveys, interviews |  |  | ✓ |  | ✓ |
| Lessard, 2019 | Canada | HIV Research | patient engagement evaluation | mixed methods | MM | observations, survey, document review |  | ✓ | ✓ |  |  |
| Pavarini, 2019 | UK | Youth Mental Health Research | PPI evaluation | mixed methods | MM | reflections, anonymous survey | ✓ |  | ✓ |  |  |
| Rarere, 2019 | New Zealand | Community Health | partnership evaluation | case study | MM | survey (online and telephone/video conference or face to face modality) | ✓ | ✓ | ✓ |  |  |
| Barger, 2019 | USA | Cancer Care Delivery Research | stakeholder engagement evaluation | nested cross-sectional study | MM | survey within pragmatic trial | ✓ |  | ✓ |  |  |
| Abelson, 2019 | Canada | Patient and Public Engagement | PPI evaluation | mixed methods | MM | survey, interview |  |  | ✓ | ✓ |  |
| Roberge-Dao, 2019 | Canada | Rehab | IKT evaluation | mixed methods | MM | document review, survey, focus groups, interviews | ✓ |  | ✓ |  |  |
| Crocker, 2019 | UK | Surgical Trials | PPI evaluation | mixed methods | MM | survey, focus group | ✓ | ✓ | ✓ |  |  |
| Hemphill, 2019 | USA | Health | PCOR evaluation | qualitative survey study | Qu | survey |  |  | ✓ |  |  |
| Langlois, 2019 | Latin America Caribbean^2^ | Health Systems Research | Implementation co-production evaluation | mixed methods | MM | survey, semi-structured interviews, document review | ✓ |  | ✓ |  |  |
| Faulkner, 2019 | UK | Mental Health Research | user lead research | mixed methods | MM | survey, researcher reflections | ✓ |  | ✓ |  |  |
| Soobiah, 2019 | Canada | Geriatrics | knowledge user engagement | cross-sectional study | Qn | survey |  | ✓ | ✓ |  | ✓ |
| Blank, 2019 | USA | Community Health | community collaboration evaluation | mixed methods | MM | survey, interview | ✓ |  | ✓ |  |  |
| Dickson, 2020 | USA | Health and Social Equity Research | partnership evaluation | cross-sectional study | Qn | survey |  | ✓ | ✓ |  | ✓ |
| Rodriguez Espinosa, 2020 | USA | Health | personal outcomes in CBPR | mixed methods | MM | survey, semi-structured interviews, focus groups, document review |  | ✓ | ✓ |  | ✓ |
| Coombe, 2020 | USA | Community Health, Equity | partnership synergy | mixed methods | MM | key informant interviews, Delphi study, cognitive interviews, survey development | ✓ |  |  |  | ✓ |
| Snijder, 2010 | Australia | Harm Reduction in Aboriginal Communities | CBPR evaluation | mixed methods | MM | document review, interviews, focus groups, survey | ✓ | ✓ | ✓ |  |  |
| O’Donovan, 2020 | UK, Uganda, Ghana | Water, Sanitation and Hygiene Research | CBPR evaluation | mixed methods | MM | workshop survey and follow-up assessment, workshop notes | ✓ |  | ✓ |  |  |
| Lucero, 2020 | USA | Health Equity and Promotion | CBPR evaluation | cross-sectional study | Qn | two surveys | ✓ |  | ✓ |  | ✓ |
| Spitzer-Shohat, 2020 | Israel | Health Inequity | health services in community organizations | mixed methods | MM | survey, telephone interviews |  |  | ✓ |  |  |
| Knudsen, 2020 | USA | Opioid Use and Harm Reduction | coalition assessment | nested mixed methods study | MM | longitudinal survey, interviews, fidelity measures within a cluster randomized trial | ✓ |  | ✓ |  |  |
| Aguirre, 2020 | USA | Diabetes Prevention | PPI evaluation | cross-sectional study | MM | survey | ✓ |  | ✓ |  |  |
| Hughes, 2020 | Ireland | Antimicrob. Stewardship | PPI evaluation | mixed methods | MM | survey, reflective session |  |  | ✓ |  |  |
| Hinrichsen, 2020 | Denmark | Mental Health Promotion | stakeholder impact and perceptions | mixed methods | MM | longitudinal questionnaire, interviews, focus groups | ✓ |  | ✓ | ✓ |  |
| Toledo-Chávarri, 2020 | Spain | Health Technology Assessment | PPI evaluation | mixed methods | MM | survey |  | ✓ |  | ✓ |  |
| Luchtenberg, 2020 | Netherlands | Pediatric Medical Research | PPI evaluation | mixed methods | MM | post-session feedback, unstructured oral evaluation | ✓ |  | ✓ |  |  |
| Gafos, 2020 | UK | HIV Prevention Research | PPI evaluation | nested cross-sectional study | MM | survey within a randomized controlled trial |  | ✓ | ✓ |  |  |
| Drebit, 2020 | Canada | Medicine | patient engagement evaluation | cross-sectional study | MM | survey |  |  | ✓ |  |  |
| Bhati, 2020 | Canada | Primary Care | patient engagement evaluation | cross-sectional study | MM | survey |  | ✓ | ✓ |  |  |
| van Schelven, 2020 | Netherlands | Chronic Disease | PPI evaluation | multiple case study | MM | Involvement Matrices, questionnaires, project reports | ✓ |  | ✓ |  |  |
| Haesebaert, 2020 | Canada | Primary Care Research | patient engagement evaluation | mixed methods | MM | logbooks, meeting materials and transcripts, observations, initial and final questionnaires | ✓ |  | ✓ |  |  |
| Vat, 2020 | Canada | Patient Engagement in Health Research | PPI evaluation | cross-sectional study | MM | survey |  | ✓ | ✓ | ✓ |  |
| Birch, 2020 | UK | Arthritis Research | PPI evaluation | cross-sectional study | MM | surveys, telephone meeting | ✓ |  |  | ✓ |  |
| Seeralan, 2021 | Germany | Mental Health in Primary Care | PPI evaluation | mixed methods | MM | focus groups, prioritization and group discussions, cognitive debriefing, survey |  |  | ✓ |  |  |
| Alexander, 2021 | USA | Pediatric Obesity Research | CBPR evaluation | mixed methods | MM | survey, interviews, document review (Parental Advisory Team outputs, meeting minutes) | ✓ |  | ✓ |  |  |
| den Houting, 2021 | Australia | Autism Research | community engagement evaluation | cross-sectional study | Qn | survey | ✓ | ✓ | ✓ |  |  |
| Scholz, 2021 | Australia, New Zealand | Mental Health | researcher partnership assessment | cross-sectional study | MM | survey | ✓ |  | ✓ |  |  |
| van Schelven, 2021 | Netherlands | Pediatric Chronic Disease | PPI evaluation | mixed methods | MM | survey, report extracts | ✓ |  | ✓ |  | ✓ |
| Hamilton, 2021 | Canada | Patient Engagement Research | patient engagement evaluation | cross-sectional study | Qn | survey |  | ✓ |  | ✓ | ✓ |
| Boursaw, 2021 | USA | CEnR | partnership assessment | cross-sectional study | Qn | survey |  | ✓ | ✓ | ✓ | ✓ |
| Loban, 2021 | Canada, Australia | Primary Care | partnership assessment | cross-sectional study | Qn | survey | ✓ | ✓ | ✓ | ✓ | ✓ |
| Martinez, 2021 | USA | Dementia Research | stakeholder engagement evaluation | cross-sectional study | MM | survey within a pragmatic comparative effectiveness trial using the Stakeholder-Centric Instrumentation Process (SCIP) |  |  | ✓ |  | ✓ |
| Moore de Peralta, 2021 | USA | Perinatal Health Disparities | partnership assessment | mixed methods | MM | survey, interviews, tool development |  | ✓ |  | ✓ | ✓ |
| Livingstone, 2021 | UK | Health Technology Assessment | PPI evaluation | cross-sectional study | MM | Survey | ✓ |  | ✓ |  |  |
| March, 2021 | USA | Pediatric Diabetes | stakeholder engagement evaluation | mixed methods | MM | Survey | ✓ |  | ✓ |  |  |
| Enard, 2021 | USA | Cancer Care Disparities | partnership assessment | mixed methods | MM | interviews, survey |  | ✓ | ✓ |  |  |
| Rasburn, 2021 | UK | Health Technology Assessment | PPI evaluation | mixed methods | MM | focused discussions, survey | ✓ |  | ✓ |  |  |
| Knowles, 2021 | UK | Participatory  Co-Design | PPI evaluation | mixed methods | MM | survey, documentary analysis, focus group, reflections | ✓ |  | ✓ |  |  |
| Nunn, 2021 | Australia | Geriatric Research | PAR evaluation | mixed methods | MM | meetings, telephone interviews, in-person event, surveys, documents within a cohort study | ✓ |  | ✓ |  |  |
| Chung, 2021 | USA | Cognitive Disability Research | stakeholder engagement evaluation | cross-sectional study | Qn | Post-trial survey |  |  | ✓ |  |  |

# **Table S2** Tool Characteristics

(n=205)

Notes: a Not available; NR not reported; NA not applicable; 1 companion report

| **First author,**  **Year** | **Tool Name** | **# Items/tool** | **Tool Purpose** | **# Tools or Tool kit (TK)** | **Assesses Outcomes (1)**  **Impacts (2)**  **Both**  **(3)** | **Partner Group(s) Targeted** |
| --- | --- | --- | --- | --- | --- | --- |
| Butterfoss, 1996 | - The Committee Member Survey (CMS) - The Plan Quality Instrument (PQI) | - Community Member survey (129)   Plan Quality Instrument (18) | CMS: to measure committees’ work and effectiveness; PQI: to measure quality of the committee plan | TK | 1 | organizational committee members |
| Goodman, 1996 | Phase 1 Formation:   - Forecast^a^ - Meeting effectiveness inventory (MEI) - Project Insight Form^a^ - Committee Survey Needs Assessment Checklist^a^ - Plan Quality Index (PQI)   Phase 2: Plan implementation (bespoke tools): Monitoring of actions, Worksheets for planning goals, processes, outcomes, impacts, Policy analysis  Phase 3: Impact:   - Key Leader Survey^a^ - Community Survey^a^ - Trend Data^a^ (bespoke) - Level of institutionalization Scale (LOIN) | - Meeting effectiveness inventory (MEI) (11) - Committee Survey (baseline) (127) - Needs Assessment Checklist^a^ (17) - Plan Quality Index (PQI) (18)   **Phase 2: Implementation (bespoke tools):** Monitoring of actions, worksheets for planning goals, processes, outcomes and impacts, policy analysis  **Phase 3: Impact:**   - Key Leader Survey^a^ (NR) - Community Survey^a^ (NR) - Trend data^a^ (bespoke) (NR)   Level of Institutionalization Scales (LOIN) (15) | To ascertain committee climate, member satisfaction, task orientation, leadership characteristics, staff support, costs and benefits of membership, committee member communication, linkages established with community organizations, and conflict resolution for three study phases. | TK | 3 | coalition members |
| Goldstein, 1997 | - Coalition Self-Assessment Tool | 63 | To aid in early coalition planning stages, trouble-shoot and to conduct self-assessment to focus on specific areas to strengthen. | 1 | 1 | coalition members committee members organization officers/staff |
| Kegler, 1998 | - Questionnaire | 55 | To address coalition factors specified in the conceptual model and two outcomes: member satisfaction and member participation. | 1 | 1 | coalition members |
| Armbruster, 1999 | - ElderCARE Community Ownership Scale (modified from the Community Ownership Scale, Flynn 1995) | 15 | To measure perception of ownership in community coalition members. | 1 | 1 | coalition partners (researchers, community members, health care organizations, government health department (state), community-based organizations) |
| Chan, 2000 | - Social Capital Index  (scale adapted from the Partnership Self-Assessment Survey, 1997) | 8 | To assess Institutional Social Capital as a functional relationship based on trust, involvement, and reciprocity, among partners in a network. | 1 | 1 | partnership members |
| Lantz, 2001 | - Detroit Community-Academic Urban Research Center (URC), Board Evaluation (Lantz et al, 2001, Schulz et al, 2003, Israel et al, 2012) v1997 and v1999 surveys (v1998 Not Available) | NR | To assess board members’ perceptions, experiences, and views about: (1) Board activities, processes, and progress; (2) principal accomplishments; (3) adherence to the principles of the project for CBPR; (4) facilitating factors; (5) barriers and challenges; and (6) hopes and recommendations for future work; and to discuss and inform changes in board activities, policies, or foci. | 2 | 3 | Coalition board members (PI, researchers, non-academic partners and institutions, civic health department; health care, non-profit, community-based organizations; community coalitions; federal government) |
| Shortell, 2002 | - Capability Index (Partnership Self-Assessment Survey (PSAS)) | 27 | To assess the perceived effectiveness of the partnership. | 1 | 1 | Coalitions |
| Weiss, 2002 | - Partnership Self-Assessment Tool | 67 | To measure partnership synergy and dimensions of functioning and gather descriptive information about the partnerships and partners. | 1 | 1 | partnership coordinators partners |
| Schulz, 2003 | - Questionnaire | NR | C1: To assess Board activities, members’ perceptions of and satisfaction with activities, achievements and process, and members’ perceptions of the challenges to and benefits of the research partnership C2: To assess overall group functioning C3: To assess perceptions of group dynamics and working relationships among members | 3 | 3 | consortium members (public, CBOs, academic institutions, health practice organizations) URC board (researchers, partner organizations) |
| Cotter, 2003 | - Log | 7 | To explore the collaborative research partnership through written logs at monthly intervals. | 1 | 3 | researchers HCP |
| Butterfoss, 2004 | - Coalition Needs Assessment (CNA) - Coalition Effectiveness Inventory (CEI) - Meeting Effectiveness Inventory (MEI) - CTAT survey | - Coalition Needs Assessment (CNA) (12) - Coalition Effectiveness Inventory (CEI) (76) - Meeting Effectiveness Inventory (MEI) (11)   CTAT Survey (10) | CNA: To identify structural elements, accomplishments, barriers to coalition success. MEI: To measure facilitation, effectiveness, meeting productivity  CEI: To identify the degree to which the elements found in effective coalitions are present. CTAT Survey: To measure perceptions of CTAT framework utility, satisfaction with the process/training, suggestions for improvement. | TK | 1 | coalition coordinators |
| El Ansari, 2004 | - Survey | 29 | To examine partnership involvement in the operational aspects of the partnerships to allow comparisons in terms of perceived benefits and costs, satisfaction, commitment, and ownership of the partnership. | 1 | 3 | partnership members (academics, health services partners (nurses, nurse managers of all levels), community members (civic volunteer organizations, youth, and women's groups, creche and church assemblies, teacher’s organizations), partnership staff (directors, coordinators, facilitators, community development workers, administrative staff) |
| El Ansari, 2004b | - Partnership Member Survey | 180 | To assess nurses' perceptions of 17 aspects of partnership functioning, in the formation and implementation of community partnerships. | 1 | 3 | health services staff (nurses) |
| Metzger, 2005 | - Partnership self-assessment survey (PSAS)-derived scales | 60 | The partnership self-assessment survey assesses coalition participants' perceptions about coalition decision making, conflict management, leadership, culture, and the effectiveness of their coalition in attaining its goals, their level of participation, and perceived participation costs and benefits. | 1 | 1 | coalition members |
| Kramer, 2005 | - Coalition Member Survey - Post Site Visit Survey | - Coalition Member Survey (59)   Post site visit survey (56) | Coalition Member Survey: to understand length of participation and perceptions of the coalition, capacity and activities of the hub organization, coalition roles, decision-making influence, coalition accomplishments, perception of benefits and challenges of participation.  Post site visit survey: (a)  coalition status (1995), (b) coalition status over time, and (c) coalition status when survey was administered. | 2 | 1 | researchers coalition staff coalition members |
| Kegler, 2005 | - Coalition Member Survey | 18 | Coalition member survey: to assess participants’ perceptions about the coalition and the entire project; describe the relationship between partnership dimensions and both interim and community-wide outcomes as perceived by coalition members. | 1 | 3 | coalition members (members from 13 CDC funded communities) |
| Cramer, 2006 | - Internal Coalition Effectiveness (ICE) Instrument | 30 | To measure outcomes and identify organizational strengths and areas for improvement. | 1 | 1 | coalition partners (leaders, members of public and private agencies) |
| Savitz, 2007 | - Partnership Strength Survey | 45 | To formally monitor and manage research partnership efforts, partner perceptions and expectations. | 1 | 3 | research partners (health system partners external consultant organisation) |
| Barber, 2007 | - Postal Survey | NR | To investigate: (i) the types of health research most associated with consumer involvement, (ii) the nature of consumer involvement, (iii) the number of recently completed NHS-research projects that meet the indicators of principles of successful consumer involvement in NHS research, (iv) the reasons for not involving consumers and (v)respondents' suggestions for improving consumer involvement in health research. | 1 | 3 | researchers patients, carers consumers and consumer organizations public |
| Feinberg, 2008 | - CTC Coalition Web-Based Self-Report Questionnaire | NR | To determine whether coalition functioning, or community characteristics predict either sustained CTC activity or new funding. | 1 | 3 | coalition members coalition staff coalition facilitators |
| Feinberg, 2008b | - CTC Coalition Web-Based Self-Report Questionnaire | wave 1 survey version (60)  wave 2 survey version (66) | To assess coalition functioning as a research tool and as part of a system for providing timely feedback to technical assistance providers and coalition members. | 2 | 3 | coalition participants technical facilitation staff |
| Wyatt, 2008 | - Evaluation of User and Carer Involvement in Primary Care Research Projects Questionnaire | 44 | To assess how and why participants came to be involved in the project, their role, whether their involvement met their expectations, and perceptions about what was or was not working well. | 1 | 3 | consumers researchers service providers academics |
| Lovell, 2008 | - Participatory Research Evaluation Questionnaire (modified from the Institute of Health Research, 1995) | 8 | To assess community participation in all aspects of the research process, from identification of the research problem, through to issues surrounding authorship. | 1 | 3 | able and disabled senior residents |
| Garner, 2008 | - Community Key Leader Survey (Goodman & Wandersman, 1996) - Coalition Group Member Survey (University of Wisconsin- Cooperative Extension, 1998) | - Community Key Leader Survey (42)   Coalition Group Member Survey (14) | Community Key Leader Survey: To measure level of concern or commitment to the coalition’s work and to determine the extent of awareness, action and concern among community leaders regarding the coalition, at intrapersonal, policy and organizational levels. Coalition Group Member Survey: To assess coalition participation, factors influencing effectiveness, coalition experiences, functioning, impact of the coalition, assessment of factors of greatest impact, and suggestions for improving group effectiveness. | 2 | 3 | community leaders coalition group members |
| Orr Brawer, 2008 | - Partnership Self-Assessment Tool (PSAT) (Lasker & Weiss, 2002) - Social Capital Survey  (Provan et al, 2013; Israel et al, 1994; Bullen & Onyx, 1995) | - Partnership Self-Assessment Tool (PSAT) (67)   Social Capital Survey (49) | PSAT: To assess synergy and partnership function Social Capital Survey: To assess trust between members and organizations, elements of reciprocity and perceptions of the value of collaboration membership, social capital at an individual- and at an aggregated community level, and individual, organizational and community empowerment and control. | 2 | 3 | coalition members |
| Adily, 2009 | - Survey | 28 | To explore the extent of and potential benefits from research collaborations. | 1 | 1 | researcher |
| King, 2009 | - Community Impacts of Research Oriented Partnerships (CIROP) | 33 | To measure community members’ perceptions of the impact of research partnerships addressing health or social issues. | 1 | 2 | researchers research partnership members (HCP, health care managers, community-based researchers, teachers, principals, academics, trainees, community leaders) |
| Van Olphen, 2009 | - Quantitative Rating Form: Adapted Community-Based Participatory Research Guidelines | 24 | To assess key stakeholders’ perceptions of alignment between a planned CBPR effort and the Guidelines. | 1 | 1 | coalition/partnership staff researchers community members |
| Sunderland, 2009 | - Partnership Self-Assessment Tool (modified from the Victorian Partnership Assessment Tool - VicHealth, 2003) | 25 | Partnership Self-Assessment Tool: To help partnership groups understand how partnership works, what a successful partnership process is; to assess partnership process functionality and identify specific areas for ongoing, sustainable partnership process improvement. | 1 | 1 | partnership members (primary care organizational representatives) |
| Tolma, 2009 | - Profile of Collaboration Survey (Chrislip & Larson, 1994) - Interactive group evaluation form - Meeting observation form - Facilitator check-off list - Random Electronic Survey | - Profile of Collaboration Survey (40) - Interactive Group evaluation Form (2) - Meeting Observation Form (33) - Facilitator Check-Off list (bespoke)   Random electronic survey (3) | Profile of Collaboration Survey: to assess stakeholder satisfaction with facets and results of collaboration. Interactive group evaluation form: to assess meeting quality using 2 open-ended questions (stakeholders) Meeting Observation Form: to rate meeting quality of (evaluators) Facilitator Check-Off List: to assess degree to which facilitator covered meeting topics or objectives.  Random Electronic Survey: to assess whether meeting objectives were met, consensus reached (if that was the intent), objectives achieved through consensus, and content was relevant and moved the group closer to project goals. | TK | 1 | stakeholders, evaluators |
| Barnidge, 2010 | - Partnership Attributes Checklist - Organizational Capacity Checklist - Intermediate-level Outcome Checklist - Taking Action - Making Improvements Tool | - Partnership Attributes Checklist (29) - Organizational Capacity Checklist (16) - Intermediate-level Outcome Checklist (41)   Taking Action - Making Improve-ments Tool | To improve the efficiency and effectiveness of a partnership (for planning, orientation, sequentially in partnership phases or periodically to assess change), to track partnership work progression, facilitate discussion and the identification of improvement areas so the partnership can progress toward long-term goals. | TK | 3 | grant recipients, grant partners |
| Blevins, 2010 | - Collaboration Scale  (based on Naylor et al, 2002) | 6 | To evaluate the collaborative research process across six dimensions (identification of need, research activities, use of resources, evaluation methods, indicators of success, and sustainability) based on a 4-point rating scale reflecting the balance of control exerted by each partner. | 1 | 3 | researchers, clinician researchers |
| Wagemakers, 2010 | - Coordinated Action Checklist | 25 | To evaluate and facilitate coordinated action in community health promotion partnerships. | 1 | 1 | partnership members |
| King, 2010 | - Community Impacts of Research Oriented Partnerships (CIROP) Questionnaire - Background Information Form for Research Partnerships - Research Contact Checklist - CIROP Respondent Form | - Community Impacts of Research Oriented Partnerships (CIROP) Question-naire (33) - Background Information Form for Research Partnerships (35) - Research Contact Checklist (4)   CIROP Respondent Form (27) | CIROP questionnaire: To measure of the mid-term impacts of partnerships addressing health or social issues by assessing community members’ perceptions of impacts. Background Information Form for Research Partnerships: To assess the partnership purpose, structural and functional features, outputs, people and organisations. Research Contact Checklist: To capture information about requests received from community members CIROP Respondent Form: To capture respondent’s awareness of the partnership’s purpose, products and information sharing, their relationship with the partnership and characteristics. | TK | 3 | community members |
| Wright, 2010 | - Critical appraisal criteria for assessing the quality and impact of user involvement on health research | 17 | To assess the quality and impact of user involvement in published research and funding applications. | 1 | 3 | researchers research readers funders and reviewers |
| Ziff, 2010 | - Wilder Collaboration Factors Inventory (Mattessich, Murray-Close & Monsey, 2001) | 44 | To assess the presence of successful collaboration factors in a partnership, organized by six partnership assessment domains. | 1 | 1 | coalition staff community members |
| Raine, 2010 | - Community Capacity Building Tool (CCBT) (Maclellan-Wright et al, 2007) | 26 | To plan, build, and reflect on community capacity in community-based health projects by reflecting on the successes and challenges of implementation and its evaluation. | 1 | 3 | partnership coordinators researchers |
| Jones, 2011 | - Jones Synergy Scale | 8 | To measure synergy in health promotion partnerships. | 1 | 1 | partnership members |
| Jones, 2011b | - Jones Trust Scale | 14 | To measure partnership trust and partnership functioning in health promotion partnerships. | 1 | 1 | health promotion partners |
| Payne, 2011 | - Questionnaire | 22 (with one additional question for Aboriginal members) | To assess compliance of CCRs' Terms of Reference with six (seven for Aboriginal CCRs) statements, to address context using Telford’s principles of successful involvement. To assess impact regarding the difference the consumer and community participation made to the project, what did/did not work well, and what changes should be made for future projects. | 1 | 3 | Aboriginal Community Reference Group HCP researchers |
| Perkins, 2011 | - CTC Websurvey for Agency Directors, Team Members - Web-Based Survey for Technical Assistants | NR | CTC Websurvey: To assess perceptions about team functioning, individual, workplace and community characteristics. Web-based Survey: to assess team dynamics perceived by technical assistants. | 2 | 3 | community members organizational administrators  technical staff |
| Sanchez, 2011 | - Coalition Self-Assessment Survey (CSAS) (modified from Kenney & Sofaer, 2000) | 52 | To assess relationship quality between the councils and the DOH; to gather data on policies and programs directly related to councils’ work, and to document the existence of internal reflection (self-evaluation) at the council level during council development. | 1 | 1 | government department members health councils (coordinators, health council members, contract monitors, health promotion staff, others) |
| VanDevanter, 2011 | - Partnership Evaluation Survey Community–Academic Partnership Functioning (modified from Israel et al, 2005) | Baseline survey (29) Follow-up survey (34) | Baseline survey: to assess general satisfaction, effectiveness, impact, trust, partnership decision making, adherence to CBPR principles, and organization and structure of meetings. The follow-up survey contained 5 additional items to assess changes in partners’ willingness to speak and express opinions at partnership meetings, trust between partnership members, and capacity of partners to work well together. | 2 | 3 | community organization staff HCP Program staff Researchers research staff |
| Bilodeau, 2011; Bilodeau, 2019^1^ | - Self-Evaluation Tool for Action in Partnership (Bilodeau et al, 2008, 2014, 2017) French and English Versions | 18 | To assess partnership function­ing by identifying difficulties and aspects that work well using six effectiveness requirements. | 1 | 1 | partnership members |
| Allen, 2011 | - online survey (modified from Israel et al, 2005) | 37 | To evaluate the research and partnership program processes between community scholars and researchers. | 1 | 3 | researchers community members |
| Curro, 2012 | - Survey | 39 | To assess the benefits and challenges of participation in the PEARL practice-based research network. | 1 | 3 | clinician researchers |
| El Ansari, 2012 | - Survey | 40 | To explore partnership members' perceived internal, external, organizational, personnel features and outcomes. | 1 | 1 | partnership members (partnership staff, health and social services staff and clinicians, academics) |
| CIHR, 2013 McLean, 2012^1^ | - KT-K2A questionnaire - KT-PHSI questionnaire - KT-synthesis questionnaire | - KT-K2A questionnaire (28) - KT-PHSI questionnaire (26)   KT-synthesis questionnaire (27) | To gather generalizable information related to funding program performance. | 3 | 3 | researchers knowledge users |
| Vale, 2012 | - MRC CTU Consumer Involvement Survey | 23 | To capture quantitative and qualitative information on whether or not studies involved consumers; how consumers had been identified and supported; the types of activities undertaken; researchers’ perspectives on the benefits and challenges of involvement and the perceived impact of consumer involvement on the research. | 1 | 2 | research staff |
| Martinez, 2012 | - Protección en Construcción (PenC) Partner Survey (based on Parker et al, 1998) | 71 | To explore group dynamics in the partnership, including questions about working relationships, satisfaction with decision making, mutual respect, and power over the decision-making process. | 1 | 1 | partnership members (researchers, staff, civic government representatives, project staff) |
| Woodland, 2012 | - Levels of Organizational Integration Rubric (LOIR) (Adapted from Gajda, 2004 and Hogue, 1993 & 1995) - Team - Collaboration Assessment Rubric (TCAR) | - LOIR (4 levels)   TCAR (26) | LOIR: To quantitatively and qualitatively gauge levels of integration over time TCAR: To assess the quality of team functioning by characteristics of dialogue, decision-making, action, and evaluation on three levels of quality. | 2 | 1 | collaborative partners |
| Tataw, 2012 | - 17-point assessment tool and framework (Narayan, 1993) | 17 | To determine the degree to which stakeholders (program designers, implementers, and beneficiaries) participated in the program, and understand cultural and structural relationships between program designers and implementers and among other stake holders (e.g., program beneficiaries) assessed to determine the degree to which they are horizontal or hierarchical, and to assess program partnership synergy. | 1 | 1 | Alliance partners (academic medical institution, community primary care provider, children, families, city government, school district, faith-based organizations, businesses, institutions of higher learning, CBOs, chamber of commerce, youth) |
| Kagan, 2012 | - Site Community Advisory Board (CAB) Survey | 37 | To assess: 1) activity frequency indicative of community involvement in protocol selection and implementation, efforts to assure research relevance, and communication and collaboration; 2) the means for identifying, prioritizing, and supporting community needs; 3) address mission-related and operational challenges faced in community-researcher collaborations. | 1 | 1 | community advisory board research staff |
| Brown, 2012 | - CTC Coalition Web-Based Survey | 41 | To assess internal coalition functioning including leadership, interpersonal relationships, task focus, participation benefits/costs, and sustainability planning and external coalition functioning (community support). | 1 | 1 | coalition members technical assistance providers |
| Braun, 2012 | - Community Involvement Measure | 27 | To measure adherence to CBPR principles. | 1 | 1 | researchers, research Staff |
| Stedman-Smith, 2012 | - Researcher survey - Community Member survey | Researchers (14) Community Members (7) | To assess demographics, objectives, satisfaction, and how the project changed behaviour and experiences of most value (community); along with demographics, objectives, and satisfaction. | 2 | 1 | researchers community members |
| Nargiso, 2013 | - General Coalition Capacities Scale - General Coalition Capacity Rubric - Environmental strategy (ES) specific capacity rubric | General Coalition Capacities Scale (10) General Coalition Capacity Rubric (19) ES specific capacity Rubric (11 capacities) | General Coalition Capacities Scale: to quantitatively measure general coalition functioning.  2. General Coalition Capacity Rubric: to measure coalition leadership, membership and staff turnover rates, quality of meetings, level of community visibility, technological capacity.  3. ES specific capacity scale: to measure specific capacity related to environmental strategy implementation. | 3 | 1 | coalition members (grant coordinator or similar designee from each community site; experts state level prevention professionals (HCP) |
| Watson-Thompson, 2013 | - Coalition Process Assessment (Community Toolbox, University of Kansas, 2021) | 255 | To assess the implementation of core tasks and activities related to each of the 12 collaborative processes outlined by the Institute of Medicine framework for collaborative public health action. | 1 | 3 | coalition staff coalition members |
| Khodyakov, 2013 | - Community Engagement in Research Index (CERI) - 3-model approach  (based on Baker et al, 1999) | 12 1 (with open-ended interview) | To understand the extent of community participation in partnered research activities and to evaluate the impact of community participation on perceived project and individual outcomes. | 2 | 1 | researchers community members |
| Patterson, 2014 | - On Becoming a Service User Researcher Survey | 32 | To describe place(s) of work, employment status, (b) research activity and experience(s) of involvement, (c) factors influencing involvement, and (d) the relationship between service use and research and the impact of involvement on mental health. | 1 | 3 | PPI |
| Rosella, 2018 Rosella, 2014^1^ | - Partnership Self-Assessment Tool (PSAT) (Weiss et al, 2002) | 67 | To assess the strengths, weaknesses and steps that can be taken to improve the effectiveness of the partnership. | 1 | 3 | health organizational members |
| Perkins, 2014 | - adapted survey  (based on PSAT(S) Cramm et al, 2011; Slaghuis et al, 2011 and Cramm et al, 2013) | 52 | To assess individual demographic, institutional, partnership and sustainability factors of academic practice partnerships | 1 | 3 | Advanced practice partners (affiliated with nursing, corporations, government, foundations, researchers, meeting attendees, nursing leaders, academic practice partnership participants) |
| Chang, 2014 | - Taiwan Health Promotion in Schools (HPS) Support Network Evaluation Study Survey | NR | To measure Health Promotion in Schools (HPS) implementation and impact, and efficacy of implementation. | 1 | 3 | teachers, director, section chief, school nurse |
| Arroyo-Johnson, 2015 | - Program for the Elimination of Cancer Disparities (PECaD) Collaborative Survey - Padres Informados, Jovenes Preparados (PI/PJ) CBPR Survey | - PECaD Collaborative Survey (60)   PI/JP CBPR Survey (45) | PECaD: To formally evaluate its adherence to and implementation of CBPR and CE principles. PI/JP: To formally measure CBPR group processes and organizational capacity to understand areas to improve participatory processes and strengthen the partnership and to understand whether perceptions of the partnership varied across sites and if this related to research implementation or outcomes. | 2 | 1 | coalition partners (researchers, community partners) |
| Brown, 2015 | - CTC Member Coalition Function Survey - CTC Functioning Survey (PCCD technical assistance providers) - Coalition function survey supplement K - Coalition function survey supplement L | - Coalition Function Survey (86) - CTC Functioning Survey (PCCD technical assistance providers) (49) - Supplement Section K (4)   Supplement Section L (65) | To assess coalition function in three domains: collaborative processes, coalition capacities, and coalition activities | 4 | 1 | coalition partners  coalition technical assistance providers  mobilizers, voluntary chairs |
| Gamble, 2015 | - Chief Investigator (CI) Survey - Patient and Public Involvement (PPI) Contributor Survey | - Chief Investigator (29)   PPI Contributor (30) | To assess opinions and motivations about Patient and Public Involvement, methods of engagement, areas of contribution and level of impact within the cohort of trials. | 2 | 3 | Researchers, research staff patient and public involvement contributors |
| Murphy, 2015 | - The Partnership Assessment Toolkit (PAT) | 30 | To enable partner discussion of the ethics of their partnership and create structures for ethical accountability. | TK | 1 | partnership members (researchers decision makers, students, civil society organization members, funding agency representatives) |
| Soper, 2015 | - RAND Europe/HERG National CLAHRC Evaluation - online survey | 45 | To understand NHS-CLAHRCs inter-relations, the perceived effectiveness of multistakeholder and multidisciplinary research for service improvement, and how research knowledge and evidence can be used to inform commissioning and clinical behaviour for patient benefit. To assess respondents’ views about CLAHRC interventions, working practices and the effectiveness and impact of CLAHRC activities. | 1 | 3 | partnership members (academics, Health care organizations, partnership leaders, health care organization administrators, funder representatives) |
| Truiett-Theodorson, 2015 | - The Wilder Collaboration Factors Inventory (modified from Mattessich, Murray-Close & Monsey, 2001) | 46 | To measure coalition efficacy on six domains of effectiveness including environment, membership characteristics, process and structure, communication, purpose, and resources. | 1 | 1 | policymakers service providers community members organizational partners |
| Wilson, 2015 | - Online Survey (based on Boote et al, 2006) | 18 | To assess how Public and Patient Involvement (PPI) is operationalised (e.g., roles, resources, training/support, recruitment, perceived impact, and to understand researchers’ experiences and perceptions of positive and negative outcomes and benefits and challenges of PPI for researchers. | 1 | 3 | Researchers |
| Bornstein, 2015 | - Member Involvement in Physical Activity Coalitions (MIPAC) Survey | 44 | To measure the perspectives of organizational representatives regarding the characteristics of physical activity (PA) coalitions, the characteristics of organizational members of PA coalitions, factors related to organizational member involvement in PA coalitions, and perceived PA coalition success. | 1 | 2 | coalition members (non-profit, for profit and government agency representatives) |
| Oetzel, 2015 | - Key Informant Survey (KIS) - Community Engagement Survey (CES) | - Key Informant Survey (KIS) (67)   Community Engagement Survey (CES) (31) | KIS: To gather relevant information about projects and identify academic and community partners.  CES: To assess perceptions of context, processes, and outcomes using a community-based participatory research conceptual model. | 2 | 3 | Researchers  Researchers academic partners community partners |
| Oetzel, 2015b | Validation of 22 scales in the:   - Community Engagement Survey (CES) | Community Engagement Survey (CES) (31) | To assess perceptions of context, processes, and outcomes using a community-based participatory research conceptual model. | 1 | 3 | academics HCP community members |
| Arora, 2015 | - Partnership Assessment in Community Based Research (PAIR) | 32 | To evaluate essential dimensions of relationships between researchers and community leaders engaged in community–academic collaborative research efforts and to create a culturally sensitive and ecologically valid measure of key characteristics of community-academic partnerships. | 1 | 3 | researchers community members |
| Stocks, 2015 | - Questionnaire (adapted from Morrow et al, 2010) | 22 | To quantitatively evaluate the quality of the public and patient involvement (PPI) within the Research User Group (RUG) that may be generalised to other settings. | 1 | 3 | research user group |
| Brown, 2016 | - Coalition Context and Capacity Assessment Survey | 18 | To assess coalition context and capacity constructs and gain a better understanding of how to improve the way community coalitions address substance abuse prevention. | 1 | 1 | coalition partners |
| Gibbons, 2016 | - Brief Online survey | 13 | To evaluate the attitudes, perceptions, beliefs, impact of, and satisfaction with the engagement process used to conduct the work of the collaborative. | 1 | 3 | community members |
| Larkan, 2016 | - Towards Successful Global Health Research Partnerships Questionnaire | 6 | To identify important features of successful partnerships. | 1 | 1 | research partners (academics, researchers, NGO members, international organization members) partnership staff research team members |
| Merkel, 2015 | - Rare Diseases Clinical Research Network Survey (Principal Investigator Version) - Rare Diseases Clinical Research Network Survey  (Patient Advocacy Group Member Version) | - Rare Diseases Clinical Research Network Survey (Principal Investigator Version) (7)   Rare Diseases Clinical Research Network Survey (Patient Advocacy Group Member Version) (7) | To assess investigators’ and Patient Advocacy Group members' perceptions of the impact of Consortium/Patient Advocacy Group collaboration and impact on the Rare Diseases Clinical Research Network (RDCRN). | 2 | 3 | researchers consortium patient advisory group members |
| Puyalto, 2016 | - Researcher questionnaire - Advisor questionnaire | - Researcher questionnaire (8)   Advisor questionnaire (6) | To gather first impressions of participants (researchers and advisors) regarding the experience of participating jointly in a research project. | 2 | 3 | Researchers, advisors |
| Robbins, 2015 | - Quarterly Survey Questions | 7 | To give patient co-investigators a safe way to share experiences, concerns, and suggestions, and to ensure team leaders receive feedback that helps improve patient co-investigator experience. | 1 | 1 | patient co-researchers |
| Finch, 2016 | - Victorian Health Promotion Foundation Partnership Analysis Tool (VPAT) - Checklist (VicHealth, 2016) | 35 | To help groups reflect on established partnerships and monitor their effectiveness in terms of how community partnerships can positively influence health. | 1 | 3 | partnership members (government promotion/safety agencies, sports professional and advocacy agencies, health insurers) |
| Goold, 2016 | - DECIDERS Steering Committee Evaluation Questionnaire (adapted from Israel et al, 2013) | 30 | To evaluate a statewide partnership process and activities and to understand what worked and why, to strengthen the partnership. | 1 | 3 | partnership members partnership staff |
| Abelson, 2016 | Public and Patient Engagement Evaluation Tool (PEET) v1.0 (2015)   - Participant Questionnaire (v1.0 June 2015) - Project Questionnaire (v1.0 June 2015) - Organization Questionnaire (v1.0 June 2015) | - Participant Questionnaire (v1.0 June 2015) (49) - Project Questionnaire (v1.0 June 2015) (39)   Organization Questionnaire (v1.0 June 2015) (30) | To assess the quality and impact of episodic and on-going Patient and Public Engagement (PPE) activities in health system organizations from three perspectives: PPE participants, PPE project sponsors and managers, and PPE leadership/capacity providers in organizations. | 3 | 3 | PPE participants  Individuals who plan, execute or sponsor PPE activities within organizations |
| Dugan, 2016 | - Program Evaluation Rating Sheet (PERS) | 36 | To document and assess process execution for implementation of a participatory intervention. | 1 | 1 | corrections officers program facilitator (research staff) |
| Brutt, 2017 | - Perceived Participation  (modified from Pollock et al, 2015) | 9 | To evaluate the perceived degree of involvement by partners. | 1 | 3 | patient partners |
| Goodman, 2017 | - Community Engagement Measure | 96 | To assess how engagement principles were adhered to by partnership members. | 1 | 1 | community member co-research trainees |
| Littlecott, 2017 | - Survey | 27 | To assess partners’ perceptions of the public health collaborative, including the degree of success (or lack of) in achieving its aims and objectives, and the degree of success of its structure, interactions (agency), and sustainability for achieving its objectives. | 1 | 3 | academics HCP partner members (HCP, local authority service providers, charitable trust staff, volunteers) |
| Scarinci, 2017 | - Evaluation Worksheet^a^ - Activity Log^a^ - Partnership Engagement Survey - Utilization and Satisfaction Questionnaire - annual progress reports^a^ - Impact evaluation (mid-term reporting)^a^ | - Evaluation Worksheet^a^ - Activity Log^a^ - Partnership Engagement Survey (90) - Utilization and Satisfaction Question-naire (50) - annual progress reports^a^   Impact evaluation (mid-term reporting)^a^ | Partnership Engagement Survey: To monitor engagement by asking partners about partnership members, structure, processes, formation, maintenance, and institutionalization, and partners' perceptions of the presence of partnership features. Utilization and Satisfaction Questionnaire: **T**o determine core utilization and satisfaction and obtain input on improvements needed for better partnership functioning. | TK | 3 | researchers PPI staff |
| Okazaki, 2017 | - Coordinating Council Member Survey | 10 | To document members’ perception of the effectiveness of- and satisfaction with adapting and implementing evidence-based health programs based on the principles of effective partnerships. | 1 | 3 | coalition staff |
| Ray, 2017 | - Stakeholder Survey | 8 | To evaluate contexts, processes, and outcomes of the stakeholder engagement process. | 1 | 1 | stakeholders (patients, caregivers physicians payers administrators) |
| Weeks, 2017 | - Environmental Scan of Patient and Public Involvement Evaluations in Health Technology Assessment Internationally | 25 | To understand HTA organizations’' organizational structure; how patients and members of the public are involved in HTA; whether and how PPI initiatives have been evaluated, and, if so, which facilitators and challenges to evaluation were found and how were results used and disseminated. | 1 | 3 | partnership members (secretariat members, 11-member organization contacts, personal contacts) |
| Jose, 2017 | - Levels of collaboration Survey (Frey et al, 2006) - VicHealth Partnership Analysis Tool and Checklist  (VicHealth, 2003) | - Levels of collaboration Survey (4)   VicHealth Partnership Analysis Tool and Checklist (7) | Levels of Collaboration Survey: To assess levels of collaboration among grant partners VicHealth Partnership Analysis (tool and checklist): To assist organizations working in partnership to assess and monitor the effectiveness of their partnerships | 2 | 3 | researchers research trainees |
| Carroll, 2017 | - Survey | 11 | Survey: To solicited participants’ understanding of patient engagement, perceptions of its value, and facilitators and challenges | 1 | 3 | network members |
| Blackburn, 2018 | - Researcher Survey with Quality-Impact Index Score - PPI Contributor Survey   with Quality-Impact Index Score | - Researcher Survey (43) with Quality Impact Index Score   PPI Contributor Survey (39) with Quality Impact Index Score | To assess the costs and consequences of PPI, to the type of PPI activities and the perceived impact of these activities on the research study and the respondent, from both researcher and PPI contributor perspectives. | 2 | 3 | researchers patient/public contributors |
| Burrows, 2018 | - Evaluation of SPHERE Advisory Group (Public Advisory Group) - Evaluation of SPHERE Public Engagement (Researchers) | - Evaluation of SPHERE Advisory Group (Public Advisory Group) (14)   Evaluation of SPHERE Public Engagement (Researchers) (12) | Advisory Group Questionnaire: To explore Public Advisory Group members' experiences of partnership and public involvement by focusing on the normative and process value systems. Researcher Questionnaire: To explore researchers' experiences of translation of public involvement activities into the research. | 2 | 3 | researchers public advisory groups |
| Forsythe, 2018 | - Annual Report Survey (researchers) - PCORI (WE-ENACT) survey (stakeholder partners) | - Annual Report Survey (researchers) (28)   PCORI (WE-ENACT) survey (stakeholder partners) (28) | To assess the short- and long-term impacts of engagement; describes engagement in PCORI projects (types of stakeholders engaged, when in the research process they are engaged and how they are engaged, contributions of their engagement); and identifies the effects of engagement on study design, processes, and outcomes selection, as reported by both PCORI-funded investigators and patient and other stakeholder research partners. | 2 | 1 | researchers stakeholder partners |
| Jones, 2018 | - Partnership survey | 27 | Partnership Survey: To assess the contribution of factors that influence partnership trust and mistrust. | 1 | 1 | partnership members |
| Kazmerski, 2018 | - Stakeholder Survey  (modified from Ray & Miller, 2017) | 8 | To examine and measure the process of stakeholder engagement, stakeholders’ attitudes toward their understanding of their roles/expectations, frequency of engagement, co-learning, and transparency. | 1 | 1 | patients/parents HCP research staff |
| Korn, 2018 | - Online Survey  (based on Wallerstein et al, 2008 & 2010, University of New Mexico Center for Participatory Research - CBPR Model, 2017) | 28 | To assess history and context, partnership dynamics, intervention and research processes, and impact and sustainability outcomes of community coalitions | 1 | 3 | researchers |
| Mann, 2018 | - Survey | - PPI contributor questionnaire (7)   Researcher questionnaire (9) | To prompt reflection on context, process and impact and enable all involved to relate their experiences in general terms, whilst providing specific examples where they wished. | 2 | 3 | researchers patient/public contributors |
| McIsaac, 2018 | - short online survey (adapted from Skinner et al, 2007) | 42 | To identify conceptual thinking and instrumental knowledge use; participants’ awareness, reception and thoughts related to the research evidence and its use for the adoption and implementation of school practices. | 1 | 3 | education leaders (principals) |
| West, 2018 | - Scale of Perceived Trustworthiness | 26 | To assess each respondent's perception of their research partner’s trustworthiness. | 1 | 1 | research partnership members (community members, including leaders, Community-based organization members, advisory boards, individuals in partnership roles; community partners, academic partner with dual roles, conference attendees, researchers) |
| Lobo, 2018 | - written response survey | 3 | To capture short, value-based statements from key stakeholders. | 1 | 3 | service providers policy makers researchers |
| Mathie, 2018 | - Completing the Feedback Cycle: Survey of PPI Representatives | 28 | To explore the frequency, mode, type, timing, importance, satisfaction of feedback, what constitutes good feedback and barriers to feedback provision. | 1 | 3 | PPI group members researchers |
| Oetzel, 2018 | Selected scales from:   - Key Informant Survey (KIS) - Community Engagement Survey (CES) | NR | KIS: to gather relevant information about projects and identify academic and community partners.  CES: to explore perceptions of context, processes, and outcomes corresponding to a community-based participatory research conceptual model | 2 | 3 | researchers  researchers, community members |
| Nowell, 2018 | - Patient Governor Engagement evaluation (based on PCORI Engagement Rubric, 2014 and PCORI Patient and Family Engagement Rubric, 2014) | 12 | To assess governance activities and data about Patient Governor Group perceptions of engagement at 3 and 6m. | 1 | 1 | patient body |
| Kendall, 2018 | - PCORI Data Collection Tool (modified from Forsythe et al, 2016) | 24 | To assess patient and stakeholder engagement including stakeholders engaged, level of engagement; the nature of stakeholder relationships; stages of engagement, facilitators of- and challenges for engagement, and engagement lessons learned | 1 | 3 | researchers |
| Walton, 2018 | - GFHS Family Council Meeting Evaluation | 14 | To assess the successes and impact of our Family Advisory Council, asking Council members whether the range and depth of discussion met their expectations and if the timing and structure of the meetings were appropriate. | 1 | 3 | family advisory council |
| Hamilton, 2018 | - Patient Engagement in Research Scale (PEIRS) | 37 | To test the effectiveness of patient engagement interventions, such as training workshops, from the patients’ perspective, using valid, reliable tools | 1 | 3 | patients, family, caregiver research partners |
| Haynes, 2019 | - Learning Matrix (bespoke to project) - Emoticon survey (based on NHMRC Guidelines for Aboriginal and Torres Strait Islander Health Research, 2003) | - Learning Matrix (bespoke to project)   Process evaluation emoticon survey (12) | **‘Most Important Learnings’ voting matrix:** to identify through brainstorming and vote preferentially on co-researcher-identified 'most important learnings' from the research.  **Emoticon survey:** to solicit from CRs the degree to which they felt the project processes adhered to six National Health and Medical Research Council (NHMRC)-based principles and engage in open-ended discussion | TK | 3 | researchers, trainers community member co-researchers |
| Goodman, 2019 | - Quantitative measure of stakeholder engagement | 32 | To assess community engagement in health research | 1 | 1 | stakeholders (patients, caregivers advocacy groups, clinicians, researchers) |
| Warner, 2019 | - Active Involvement of Users in Research Observation Schedule and Assessment Questionnaire | 12 | To pilot and methodologically appraise the Active Involvement of Users in Research Observation Schedule and Questionnaire using a case study. | 1 | 3 | researchers |
| Ken-Opurum, 2019 | - modified Coalition Self-Assessment Survey (CSAS) (modified from Kenney and Sofaer, 2000) | 43 | modified CSAS: To measure members’ perceived effectiveness at the organization/ participant-level. | 1 | 3 | health coalition members |
| Tabriz, 2019 | - Semi-structured questionnaire | 6 | To solicit information regarding individuals’ goals and priorities for the development of the programme, their perceptions of the processes used during the partnership, challenges faced, how participation may have enhanced knowledge and skills and how participants perceived the e-assist programme fit (or did not fit) with clinic workflows and processes. | 1 | 3 | healthcare organization leaders and staff researchers research staff |
| Duran, 2019 | - Key Informant Survey (KIS) - Community Engagement Survey (CES) | NR | To assess research context, process, and outcome measures, including measures of power/resource sharing and structural characteristics of projects. | 2 | 3 | researchers academics community partners |
| Lessard, 2019 | - Patient Engagement Satisfaction Survey - Attributed Level of Patient Engagement  (International Association of Public Participation’s spectrum of engagement (IAP2), Alberta Health Services, 2012; Kirwan et al, 2017; Bellows et al, 2015) | - Patient Engagement Satisfaction Survey (14)   Attributed Level of Patient Engagement (1) | To describe the nature of PE, the level of PE, the impacts of PE from the patient perspective. | TK | 3 | researchers community-based researchers CBOs research participants |
| Pavarini, 2019 | - anonymous assessment questionnaire | 13 | To assess participants and researchers as part of running the YPAG. To understand YPAG members' experiences, including perceptions about the right level of information, training, support, and compensation, and to reflect upon the learning and skills they might have gained from participating and any impact on academic and personal development. To continuously assess and document how the project has changed the research. | 1 | 3 | researchers youth researchers |
| Rarere, 2019 | - Evaluation Tool | 62 | To measure how well community and academic research partners and the partnership as a whole are following the framework, while in the process of developing and implementing an intervention. | 1 | 1 | research partnership members |
| Barger, 2019 | - TrACER Quality of Engagement Survey (External Stakeholder Advisory Group (ESAG) Feedback Survey) | 21 | To evaluate stakeholder engagement, satisfaction, and impact on the trial by addressing communication, meeting structure, involvement and input, respect and value and ESAG areas of interest. | 1 | 3 | stakeholders |
| Abelson, 2019 | Public and Patient Engagement Evaluation Tool (PPEET) (2018 version)   - Participant Questionnaire (Modules A-B) - Project Questionnaire (Modules A-C) - Organization Questionnaire | - Participant Questionnaire  (Module A: 21, Module B: 22, Demographic Questions (optional) (6) - Project Questionnaire (Module A: 15, Module B:16, Module C: 8)   Organization Questionnaire (30) | PPEET: to provide a generic evaluation tool for health system organizations to evaluate and continuously improve the quality of public and patient engagement (PPE) activities. Participant survey: to assess processes, outputs, and perceived impacts of engagement activities  Project survey: to evaluate planning and execution of engagement activity from engagement implementers (e.g., engagement project leads, staff members) perspectives.  Organization survey: to assess culture and practices supporting public and patient engagement from senior management and leadership perspective Feedback survey: Quantitative (survey) data provided insights into the implementation of the PPEET and its appropriateness and feasibility | 3 | 3 | patients, public, family organizational staff |
| Roberge-Dao, 2019 | - Survey questionnaire | 20 | To gather descriptive information on the nature of the projects, the outcomes, and the partnerships. | 1 | 3 | researchers clinicians |
| Crocker, 2019 | - Survey | 38 | Survey: The primary objective of this survey was to ascertain current PPI practice in UK surgical trials and respondents’ attitudes towards PPI. | 1 | 3 | researchers PPI HCP research staff |
| Hemphill, 2019 | - PCORI Ways of Engaging‐Engagement Activity Tool (WE‐ENACT) | 28 | The survey included closed‐ and open‐ended questions about partners’ characteristics and experiences engaging in the PCORI project. | 1 | 2 | patients, family caregivers, representatives of patient/caregiver advocacy and CBOs |
| Langlois, 2019 | - self-administered questionnaire | 28 | To assess (1) the level of engagement in different stages of the embedded research; (2) the extent to which their capacities to conduct and use research was strengthened; and (3) the level of confidence in undertaking implementation research activities. | 1 | 3 | decision makers |
| Faulkner, 2019 | - Post-Interview Brief Survey | 5 | To invite participants to respond anonymously and reflect on their experiences of being involved in the study. | 1 | 1 | service users (user led research) |
| Soobiah, 2019 | - modified Patient Engagement Evaluation Tool (PEET) (Moore et al, 2015) | 17 | To quantify engagement using evidence-informed criteria from the stakeholder engagement in comparative effectiveness research framework. | 1 | 1 | patients caregivers geriatricians |
| Blank, 2019 | - Partnership Evaluation Survey (based on Weiss et al, 2002; Schulz et al, 2003; El Ansari, 2003) | 45 | To assess the 1) partnership, composition, structure, and function; 2) group process measures; and 3) intermediate indicators of partnership effectiveness over time. | 1 | 1 | clinical and academic researchers HCP and administrators |
| Dickson, 2020 | - Key Informant Survey (KIS) (English and Spanish translation versions) | 70 | To capture key “factual” information about the project and partnership that could be identified by a PI or designate. | 1 | 1 | researchers community members |
| Rodriguez Espinosa, 2020 | CBPR Processes and Practices, and outcomes scales (from E2 Key Informant (KIS) and Community-engagement Surveys (CES)) | NR | KIS: To gather collected project-related information (e.g., funding dates, financial resource sharing, and use of formal agreements).  CES: To obtain perceptions of CBPR model constructs including partnership processes (relational and structural dynamics), and individual and project outcomes. | 2 | 1 | community partners academic partners |
| Coombe, 2020 | - Partnership Synergy Questionnaire | 7 | To measure partnership synergy in the context of success in long-standing CBPR partnerships that promote social and health equity. | 1 | 1 | researchers academic experts community experts |
| Snijder, 2010 | - 7-point participation scale  (based on Pretty, 1995; Wagemakers et al, 2008) | 4 | To measure participant’s preferred and actual levels of participation across four project phases: needs assessment, development, implementation, and evaluation. | 1 | 3 | Aboriginal community members Aboriginal HCP Aboriginal community partners |
| O’Donovan, 2020 | - Post-Workshop Survey - Community Follow-up Assessment | - Post-workshop survey (11)   Community Follow-up Assessment (10) | Post-workshop Survey: To establish whether participatory methods were valued, whether the approaches could be further improved and any other comments. Community Follow-up assessment: To assess longer term impact and establish whether the visit was remembered, advice would be recalled, there was positive behaviour change and whether improvements could be made. | 2 | 3 | village health team, community health workers, Ghanaian community water/sanitation agency, researchers, research trainees, manager, NGO facilitators, members of the NGO forum, Deputy District Health Officer (DDHO), Village Elders |
| Lucero, 2020 | CBPR Process Scales (synergy, trust, CBPR principles, participation, influence) and Trust Typology (from E2 Community Engagement Survey (CES)) | NR | To test the quantitative structural elements of the trust typology, identify variability in trust correlates, and create an empirical foundation for the trust types. | 2 | 1 | researchers academic partners community partners |
| Spitzer-Shohat, 2020 | - Community Impact of Research Oriented Partnerships (CIROP) questionnaire | 33 | To measure the extent to which the partnership improved knowledge and research skill development, organisational development, and access/use of information. | 1 | 3 | community partners |
| Knudsen, 2020 | - coalition member and key stakeholder survey | NR | To measure internal and external context, and other coalition factors. | 1 | 1 | coalition members key stakeholders |
| Aguirre, 2020 | - Community Advisory Board Evaluation | 6 | To evaluate member perspectives on Community Advisory Board members' functioning and to help the facilitators improve Community Advisory Board functioning. | 1 | 3 | community advisory board members |
| Hughes, 2020 | - Patient and Public Education Evaluation Tool (PPEET v1.0) - Participant Questionnaire | 19 | To understand the successes and positive experiences of being involved and to provide an opinion on areas that could be improved. | 1 | 3 | patient representative group |
| Hinrichsen, 2020 | - Expanded Evaluation Questionnaire (perceived impact) | Expanded Evaluation Questionnaire (perceived impact) (14) | To explore partners' motivation for joining the network, history of participation, local organisation, and dissemination; collaboration with other partners and local collaborations, development of new materials and activities; facilitators and positive experiences using the ABC-framework, obstacles and barriers, and recommendations to other partners. | 2 | 2 | coordinators partner organization leaders student organization union |
| Toledo-Chávarri, 2020 | - Questionnaire (Spanish language) (adapted from Popay, Collins, and the PiiAF Study Group, 2014) | 9 | To undertake a deeper evaluation of the PI initiatives’' impacts upon presentation of PI experiences. | 1 | 3 | researchers |
| Luchtenberg, 2020 | - Feedback Form and Unstructured Oral Evaluation | 9 | To receive feedback from co-researchers after the analysis was complete and further evaluate the process orally. | TK | 3 | child co-researchers PPI researchers |
| Gafos, 2020 | - PROUD Researcher Survey - PROUD Community Member Survey | PROUD Researcher Survey (7)  Community Member Survey (15) | To capture information on the process and impact of the involvement. | 2 | 3 | community representatives researchers |
| Drebit, 2020 | Public and Patient Engagement Evaluation Tool (PPEET) V2.0   - Organizational Questionnaire - Project Questionnaire - Patient Partners Survey | Public and Patient Engagement Evaluation Tool (PPEET) V2.0   - Organizational Questionnaire (30)   Project Questionnaire - Patient Partners Survey (22) | To measure patient engagement in patient and non-patient partners. Patient partners were asked to provide feedback on their participation in the Network; non-patient partners were asked to reflect on how the organizational requirements for PI influenced their involvement with the Network and on how engagement exists within the organization. | 2 | 3 | Emergency Medicine network partners  patient partners |
| Bhati, 2020 | - Web-based evaluation survey (based on Ableson et al, PPEET; PCORI We-ENACT tools) | 44 | To assess four dimensions of Patient Partners’ experience: (i) experience during meetings; (ii) views on collaboration; (iii) appropriateness of support provided to engage; and (iv) roles and involvement in research; and to explore Patient Partners’ motivation to engage, key strengths of the collaboration, suggestions for improvement, training required, and contributions made to the research study. | 1 | 3 | researchers research staff patient partners |
| van Schelven, 2020 | Involvement Matrix   - project report - questionnaire | Involvement Matrix   - project report (4)   questionnaire (8) | To gain insight into how young people were involved in projects, from the perspective of young people. | TK | 3 | youth partners |
| Haesebaert, 2020 | - Logbooks^a^ - Meeting audiorecordings^a^ - EQUIPPS - GMF Initial Questionnaire for Quality and Patient-Oriented Research (Patient, Caregiver) (French language) - EQUIPPS - GMF Initial Questionnaire for Quality and Patient-Oriented Research (Clinicians, Administrators) (French language) | - Logbooks^a^ (NA) - Meeting audio-recordings^a^ (NA) - Patient, Caregiver Questionnaire (16)   Clinicians, Administrators Questionnaire (15) | To assess the acceptability and feasibility of the councils using self-administered questionnaires with other methods. | TK | 2 | Patients, caregivers HCP partnership manager |
| Vat, 2020 | - Patients as Partners in Research (patient partner survey) - Patients as Partners in Research (researcher survey) - Student Survey (adapted from Patient Canada Evaluation Tools and Wilson et al, 2015) | - Patients as Partners in Research (patient partner survey) (28) - Patients as Partners in Research (researcher survey) (25)   Student survey (22) | To compare the perspectives of researchers and patients with a focus on and experience with involvement in research projects over time. | 3 | 3 | researchers patient partners |
| Birch, 2020 | - Survey for researchers - Survey for Patient Research Partners | - Survey for researchers (11)   Survey for Patient Research Partners (19) | To assess the impact of PPI on individual work packages and on EuroTEAM overall. | 2 | 3 | patient research partners researchers |
| Seeralan, 2021 | Public Patient Engagement Evaluation Tool (PPEET) - (German Version)   - Patient Questionnaire Module A and B - Project Questionnaire Modules A-C | Public Patient Engagement Evaluation Tool (PPEET) - (German Version)  Patient Questionnaire Module A and B (21,22) Project Questionnaire Modules A-C (15, 16, 8) | To assess process (e.g., communication and support for participation) and outcome evaluation (e.g., impacts and influence of PPI) of the engagement components (workshops and Participatory Research Team). | 2 | 3 | participants  researchers patient partners |
| Alexander, 2021 | - Community Capacity Evaluation Survey | Community Capacity Evaluation Survey (42) | To assess group dynamic dimensions members determined were most relevant to their year 1 success including communication, problem assessment, participation and personal influence, leadership, community power, collective efficacy, and overall satisfaction. | 1 | 1 | parent advisory team |
| den Houting, 2021 | - Academic and Community partner survey | 52 | To investigate when, how, and how often community partners were involved in Autism CRC research projects and how academic and community partners perceived such engagement. | 1 | 3 | researchers community members |
| Scholz, 2021 | - Consumers as Researchers in Mental Health (CaRiMH) survey | 138 | To measure the views of non-consumer researchers about consumer research and experiences of collaboration. | 1 | 3 | researchers |
| van Schelven, 2021 | - Project Outcome Scale | 15 | To assess the self-perceived contribution of projects to the social position of Young Persons with Chronic Conditions. | 1 | 3 | patients |
| Hamilton, 2021 | - Patient Engagement in Research Scale (PEIRS-22 shortened version)  (modified from Hamilton et al, 2018) | 22 | To assess the degree of meaningful engagement of patients and family caregivers as partners in research projects. | 1 | 3 | patients, family caregivers |
| Boursaw, 2021 | - Community Engagement Survey (scales (7) with subscales (23)) | NR | To assess researcher and community member perceptions of CBPR contexts, mechanisms, and outcomes. | 1 | 3 | researchers, community members |
| Loban, 2021 | - IMPACT Partnership Questionnaire (Weiss et al, 2002; Jones & Berry, 2011] | 42 | To assess partnership functioning and synergy. | 1 | 3 | partnership stakeholders |
| Martinez, 2021 | - Stakeholder-Centric Engagement Evaluation | 110 | To develop a quantitative measure of stakeholder engagement aligned with the research team and advisory committee approach, and to identify and establish clear procedures for stakeholder engagement throughout the study. | 1 | 1 | researchers advisory committee |
| Moore de Peralta, 2021 | - revised CBPR-PTS instrument (English and Spanish Language Versions) | 195 | To better understand trust of people and organizations, and trust in the programs and activities conducted by PASOs | 2 | 1 | stakeholders (community health workers, community participants, volunteer community health workers, organizational partners) |
| Livingstone, 2021 | - Highly Specialized Technologies (HST) Impact Form (v1, v2) - Interventional Procedures (IP) Impact Form (v1, v2) | - Highly Specialized Technologies (HST) Impact Form (v1, v2: 3,4)   Interventional Procedures (IP) Impact Form (v1, v2: 2, 7) | HST Questionnaire: To capture impact of Patient Advisory Group on HST guidance and provide feedback and direction to Patient Advisory Groups for future engagement. IP Questionnaire: To capture impact of Patient Advisory Group on IP guidance and provide feedback and direction to Patient Advisory Groups for future engagement. | 4 | 2 | HST and IP committee members including HCP, NHS staff, researchers, public, funder, industry |
| March, 2021 | - Stakeholder Survey (adapted from Ray & Miller, 2017 and Kazmerski et al, 2019) | 8 | To assess the research context, processes for engagement and evaluation of processes, stakeholder contributions, and impact on research. | 1 | 1 | stakeholders included parents nurses clinicians |
| Enard, 2021 | - Survey | 6 | To examine group dynamics, and their influence on achieving shared goals, within a CBPR-guided partnership among underserved African American communities. | 1 | 2 | academics community partners community collaborators |
| Rasburn, 2021 | - Early engagement survey | 127 | To anonymously capture patient stakeholders’ perceptions and processes related to working with NICE, and to gather impressions of impact and feedback, in 11 domains. | 1 | 3 | patients |
| Knowles, 2021 | - Survey (based on the Generic Learning Outcomes Framework, Arts Council UK) | 5 | To capture potential patient engagement impacts under five headings: Knowledge & Understanding, Skills, Attitudes & Values, Enjoyment, Inspiration & Creativity, and Behaviour & Progression. | 1 | 3 | co-researchers |
| Nunn, 2021 | - Survey | 11 | Survey: To assess and integrate views and perspectives of those co-designing and delivering the process, and report how participant involvement activities positively impacted the study design. | 1 | 3 | researchers participants participant advisors team (researchers, assessors) |
| Chung, 2021 | - Patient Engagement in Research Scale (PEIRS) instrument | 39 | To assess meaningful patient engagement throughout the research process from the patient perspective. | 1 | 1 | researchers, trainees, academics parents health care providers non-profit organizational leaders |
